# Expansion and Diversification of BTL Ring-H2 Ubiquitin Ligases in Angiosperms: Putative Rabring7/BCA2 Orthologs

**DOI:** 10.1371/journal.pone.0072729

**Published:** 2013-08-08

**Authors:** Victor Aguilar-Hernández, Juliana Medina, Laura Aguilar-Henonin, Plinio Guzmán

**Affiliations:** Departamento de Ingeniería Genética de Plantas, Centro de Investigación y de Estudios Avanzados, Unidad Irapuato, Irapuato, Guanajuato, México; Kyushu Institute of Technology, Japan

## Abstract

RING finger E3 ligases are components of the ubiquitin proteasome system (UPS) that mediate the transfer of ubiquitin to substrates. Single-subunit RING finger E3s binds the E2 ubiquitin-conjugating enzyme and contains recognition sequences for the substrate within the same polypeptide. Here we describe the characterization of a class of RING finger E3 ligases that is conserved among eukaryotes. This class encodes a RING-H2 domain related in sequence to the ATL RING-H2 domain, another class of E3 ligases, and a C2/C2 zing finger at the amino-terminus, formerly described as BZF. In viridiplantae (green algae and land plants), we designed this family as BTL for *B*ZF A*TL*s. BTLs are putative orthologs of the mammalian Rabring7/BCA2 RING-H2 E3s that have expanded in angiosperms. They are found in numbers ranging from three to thirty-one, which is in contrast to the one to three members normally found in animals, fungi, and protists. Furthermore, the number of sequence LOGOs generated in angiosperms is four times greater than that in other eukaryotes. In contrast to *ATL*s, which show expansion by tandem duplication, tandemly duplicated BTLs are scarce. The mode of action of Rabring7/BCA2 and BTLs may be similar since both the Rabring7/BCA2 BZF and the ath|BTL4 BZF are likely to mediate the binding of ubiquitin. This study introduces valuable information on the evolution and domain structure of the Rabring7/BCA2/BTL class of E3 ligases which may be important for core eukaryotic genes.

## Introduction

The dynamic assembly and size difference of gene families among species has played an essential role in the evolution of eukaryotic genomes. Gene families consist of homology-related genes that have evolved from a common ancestor by gene duplication events, which usually preserves similar functions as well as a similar protein domain architecture [1]. Ubiquitin ligases, or E3s, are enzymes of the ubiquitin proteasome system (UPS) that have evolved in plants as distinct types of multigene families. There are also plant-specific E3 ligases as well as E3s that display common features among plants and other eukaryotic organisms [2,3].

Most aspects of the life of an organism are controlled by the regulated synthesis of novel polypeptides and the precise degradation of existing proteins; the UPS is a main mechanistic route for the regulated control of protein levels, which relies on ubiquitin, a 76 amino acid-long protein tag, covalently attached to the substrate [4]. The E3 ligases coordinate the transfer of ubiquitin to a substrate by recognizing the target protein and the ubiquitin-conjugating enzyme (E2), which is a component of the UPS that carries the activated ubiquitin [3]. Approximately 1400 E3 ligase genes are predicted in the *Arabidopsis thaliana* genome, and a little more than 600 have been predicted in the human genome [2,5]. One important protein domain in E3 ligases is the Really Interesting New Gene (RING) finger, which was initially identified in the human RING1 protein and subsequently in a myriad of proteins that are implicated in assorted and specific cellular phenomena [6]. The projected number of RING finger E3 ligases is 477 in *A. thaliana* and 300 in human [2,3].

The overall domain architecture and structural features of the RING domain have been used to classify RING-finger E3 ligases [7]. For instance, the RING-H2 Arabidopsis Tóxicos en Levadura (ATL) E3 ligase family is a plant-specific multi-gene family that includes the RING-H2 variant of the canonical RING finger domain and a putative transmembrane domain located at the N-terminus. This family is widespread in plants, possessing 20 to 162 members in different species [8]. The structure of the RING finger is conserved in ATLs. The canonical domain consists of eight spaced residues that coordinate the binding of two Zn^2+^ ions: seven cysteines and one histidine with the consensus sequence Cys-X(2)-Cys-X(n)-Cys-X(1)-His-X(2)-Cys-X(2)-Cys-X(n)-Cys-X(2)-Cys. The RING-H2 variation contains a histidine residue in place of the fifth cysteine [9]. An analysis of 1815 ATLs indicated that the spacing between the residues involved in zinc ligation is preserved in all of them, with the consensus sequence Cys-X(2)-Cys-X(15)-Cys-X(1)-His-X(2)-His-X(2)-Cys-X(10)-Cys-X(2)-Cys [10]. During our early studies of the ATL family, we identified several proteins harboring closely related RING-H2 domains. One group of them consisted of four *A. thaliana* proteins that contained a four cysteine residue motif located toward the N-terminus in a pattern similar to that of a C2/C2 zinc finger [11]. This class of RING-H2 ligases has been described in humans, and to date a few of them have been analyzed in *A. thaliana*.

Rab7-interacting RING finger protein (Rabring7), which was isolated as a Rab7 interacting protein, belongs to this C2/C2 RING-H2 class of E3 ligases. Rab7 is a member of the Rab family of small G proteins that have a role in intracellular vesicle traffic regulation [12]. Rabring7 has also been named Breast Cancer Associated gene 2 (BCA2) because it was identified as a differentially-expressed gene using normal and cancerous mammary epithelial cell lines [13]. Rabring7/BCA2 is an unstable protein that has E3 ligase activity dependent on the RING-H2. The amino-terminal C2/C2 zinc-finger, termed BCA2 Zinc-Finger (BZF), interacts with ubiquitin. Importantly, lysine residue mutations in the BZF domain abolish the E3 ligase activity (autoubiquitination) [14]. Analysis of interacting proteins revealed that various degradation processes control Rabring7/BCA2 stability. The interaction with the human homolog of RADIATION SENSITIVE23a (hHR23a), a conserved adaptor protein among eukaryotic organisms that possesses Ubiquitin-like/Ubiquitin-associated (UBL/UBA) domains, significantly reduces the autoubiquitination activity, resulting in Rabring7/BCA2 stabilization. Similarly, the interaction with 14-3-3sigma protein also stabilizes this E3 ligase, but through a different mechanism [15]. Besides its role in breast cancer, Rabring7/BCA2 exhibits other functions in the cell. It interacts with Tetherin, a membrane-anchored protein that retains HIV-1 particles during the final phase of viral replication, and promotes internalization and degradation of these particles [16]. It also has a role in the regulation of Epidermal Growth Factor Receptor (EGFR) trafficking for lysosomal degradation [17] and assists in the degradation of the proto-oncogene c-Myc through a complex with MM-1, which is a tumor suppressor that binds to the myc box II [18]. A Rabring7/BCA2-like protein is present in vertebrates. RNF126 contains BZF and RING-H2 domains that are highly similar to Rabring7/BCA2, and thus also displays E3 ligase activity. It is likely that Rabring7/BCA2 and RNF126 may have related functions [19].

Four putative *Rabring7/BCA2* orthologs have been studied in *A. thaliana*. The COP1 Interacting Protein 8 (CIP8) was identified as a COP1-binding protein [20]. COP1, a repressor of photomorphogenesis in darkness, is an RING E3 ligase that mediates degradation of the transcription factors HY5, HYH, and LAF1, among other proteins. CIP8 promotes ubiquitination of HY5 *in vitro* and interacts weakly with HY5, but strongly interacts with COP1 and AtUBC8, which is an E2 ubiquitin conjugase. The RING-H2 domain of CIP8 is essential for the E3 activity. It is possible that CIP8 may assist in the ubiquitination of substrates recruited by the E3 COP1 [21]. AtRDUF1 and AtRDUF2 are two related proteins identified by *in silico* analysis that play a role in the response to dehydration mediated by abscisic acid (ABA). They also possess autoubiquitination activity depending on the RING-H2 domain. AtRDUF1 and AtRDUF2 are induced by drought as well as in response to ABA treatment. The *atrduf* mutants are hyposensitive to ABA and susceptible to dehydration. Although these genes have functional redundancy, each one has a distinct role mediating the stress response [22]. *RHC1*, another putative *Rabring7/BCA2* ortholog gene, was identified in *Zea mays* through a transcriptional analysis of the stem cell niche in roots and exhibits reduced expression in the quiescent center (QC). *A. thaliana* lines overexpressing *RHC1* have alterations in the apical architecture of the root. The root meristem becomes exhausted with abnormally large QC cells and an extremely disorganized root cap (RC) [23].

To extend our study on the evolution and functional analysis of RING finger E3 ligases, we retrieved putative *Rabring7/BCA2* ortholog sequences from genome databases of plants, animals, fungi, and protozoa. We named this gene family in plants as *B*ZF A*TL* (BTL). We identified 403 members of this family from 33 plant genomes and 99 members from 74 genomes of other eukaryotes. We also uncovered conserved motifs among groups of Rabring7/BCA2/BTL proteins by generating 73 position-specific probability matrix (PSPM) sequence LOGOs. Using a yeast two-hybrid system, we uncovered potential protein–protein interactions mediated by members of this family. Our study revealed meaningful results on the evolution of *Rabring7*/*BCA2*/*BTL* genes, in addition to information concerning domain structure of this class of RING-H2 zinc finger genes.

## Results

### Functional constraint on the RING-H2 domain in BZF ATLs (BTL) E3 ligases

**Figure 1:**
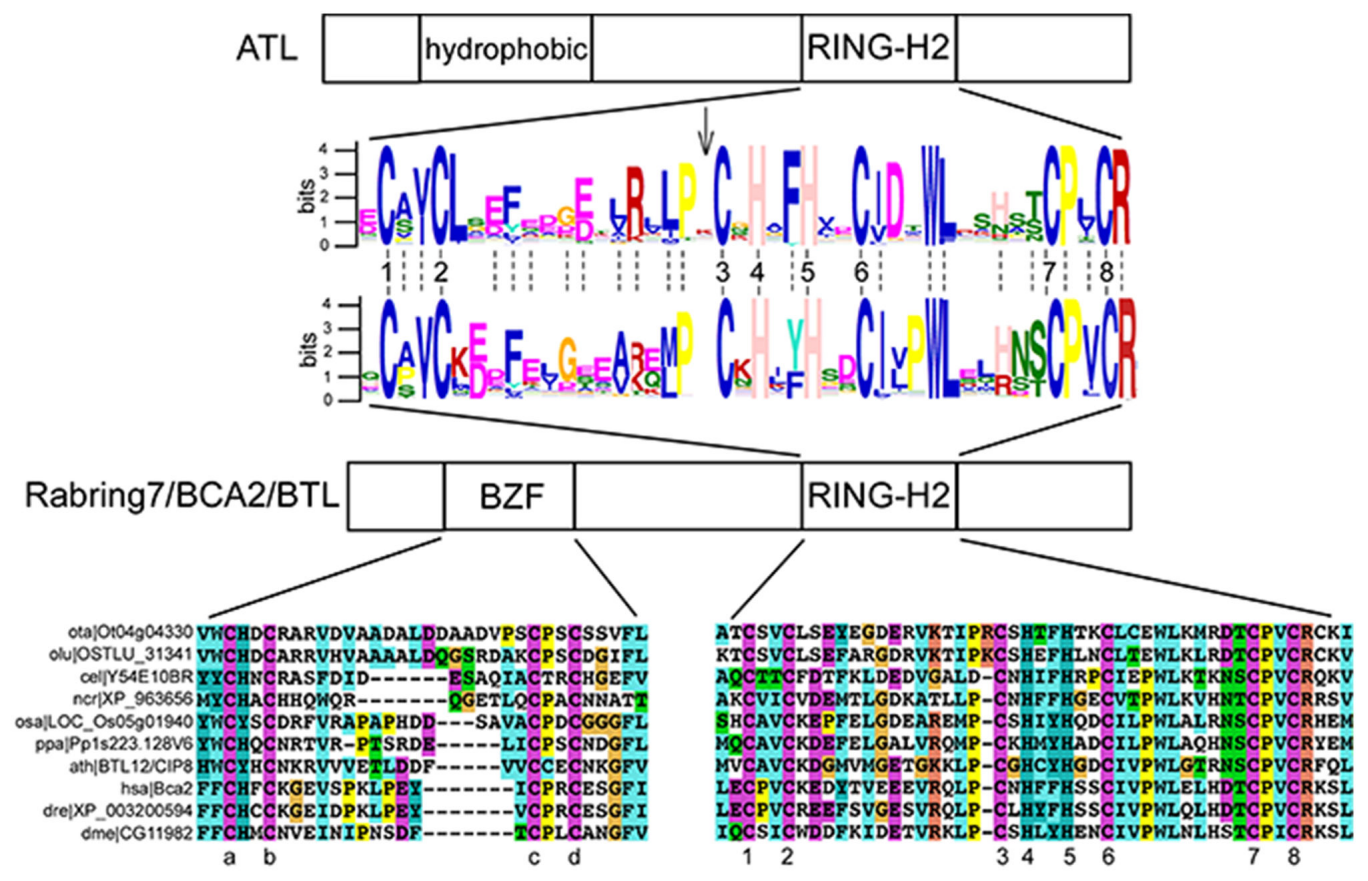
Overall domain comparison between ATL and Rabring7/BCA2/BTL RING-H2 ubiquitin-ligases. A schematic representation of canonical ATL and Rabring7/BCA2/BTL E3 ligases indicating the position of the two relevant domains on each class: hydrophobic and RING-H2 in ATLs and BZF and RING-H2 in Rabring7/BCA2/BTLs. A sequence LOGO comparison between the two RING-H2 domains is shown; LOGOs were generated from the collected Rabring7/BCA2/BTLs sequences and from a previous analysis of ATLs. The numbers indicate the residues involved in zinc ligation. The arrowhead indicates an absent amino acid residue in Rabring7/BCA2/BTLs and broken lines indicate conserved residues between the two RING-H2 domains. An alignment of representative proteins displaying the BZF and the RING-H2 regions of Rabring7/BCA2/BTLs is displayed below. ClustalX was used for sequence alignment and a default color code was applied. The numbers indicate the residues involved in zinc ligation in the RING-H2 domain and letters the conserved cysteines in BZF.

Our earlier search for ATL-related genes led us to the identification of a group of RING-H2 coding genes that encoded a RING finger highly related to the ATL-RING-H2 domain [11]. We formerly identified four *A. thaliana* proteins that contained four cysteine residues in a pattern similar to that of a C2/C2 zinc finger motif at the amino-terminus and in an analogous location where a hydrophobic region is present in the ATL E3s (Figure 1). A related motif was identified in the BCA2 protein and named the BCA2 Zinc-Finger (BZF) domain [14]. We named these genes “*BTL*s” for *B*ZF A*TL*s, which are the putative plant orthologs of these RING-H2 finger genes that encode a BZF. We will refer to *Rabring7/BCA2* for the animal, fungal, and protist genes and *Rabring7*/*BCA2*/*BTL*s for the eukaryotic gene.

A comparison of the sequence LOGOs generated from the RING-H2 domains from Rabring7/BCA2/BTLs and ATLs showed high similarity between both domains (broken lines between the alignment in Figure 1). The same spacing between the cysteines and histidines residues defining the domain was also observed (numbered 1 to 8 in Figure 1), except for the spacing between the second and third cysteines, which is one residue shorter (indicated by the arrow in Figure 1). This missing residue may correspond to the residue between a conserved proline and the third cysteine. This proline is highly conserved in ATLs and Rabring7/BCA2/BTLs, and is adjacent to the third cysteine in Rabring7/BCA2/BTLs. Thus, there may be functional constraints on the placement of the residues that structure the RING-H2 domain in these two classes of E3 ligases.

*Rabring7*/*BCA2*/*BTL*s were readily identified across several eukaryotic proteomes and contained a BZF and RING-H2 domain (see alignment in Figure 1). Few deviations from the spacing of the canonical RING-H2 were detected. A noteworthy case was the BTL ortholog from the green algae species *Ostreococcus* *lucimarinus* and *Ostreococcus* *tauri* , which had spacing between the residues that defined the domain resembling ATLs, as there was a residue located between a conserved proline residue and the third cysteine (see ota|Ot04g04330 and olu|OSTLU_31341 in the alignment in Figure 1). However, the evolutionary implication of this observation was not further explored.

### Identification of RING finger Rabring7/BCA2/BTLs across eukaryotes

We previously identified four *A. thaliana* members of the BTL gene family and latter linked them as putative *Rabring7/BCA2* orthologs. To assess the evolutionary history and gain insights on the domain structure of this class of RING-H2 proteins, we first surveyed members of the *Rabring7*/*BCA2*/*BTL*s gene family across genomes from plants, animals, fungi, and protists. We searched for proteins using BLASTP and the Hidden Markov Model (HMM) based on the four formerly identified *A. thaliana* proteins and from the human BCA2, first using a 41 amino acid long sequence that included the Rabring7/BCA2/BTLs RING-H2 domain, and then using the BZF-like domain (see Material and Methods). First, a group of 2356 hits consisting of RING-H2 sequences were obtained. Then, 502 proteins were retrieved that showed a BZF-like domain at the amino-terminal end. The retrieved proteins that contained the BZF-like domain also included the canonical BTL RING-H2 domain, suggesting that this distinct domain architecture came from a common ancestor. In two Basidiomycetes, a group of Apicomplexans protists, and a few plant species, the retrieved proteins displayed insertions that increased the spacing between two of the residues coordinating zinc ligation within the RING-H2 domain; these proteins were included in the analysis (see Materials and Methods).

**Figure 2:**
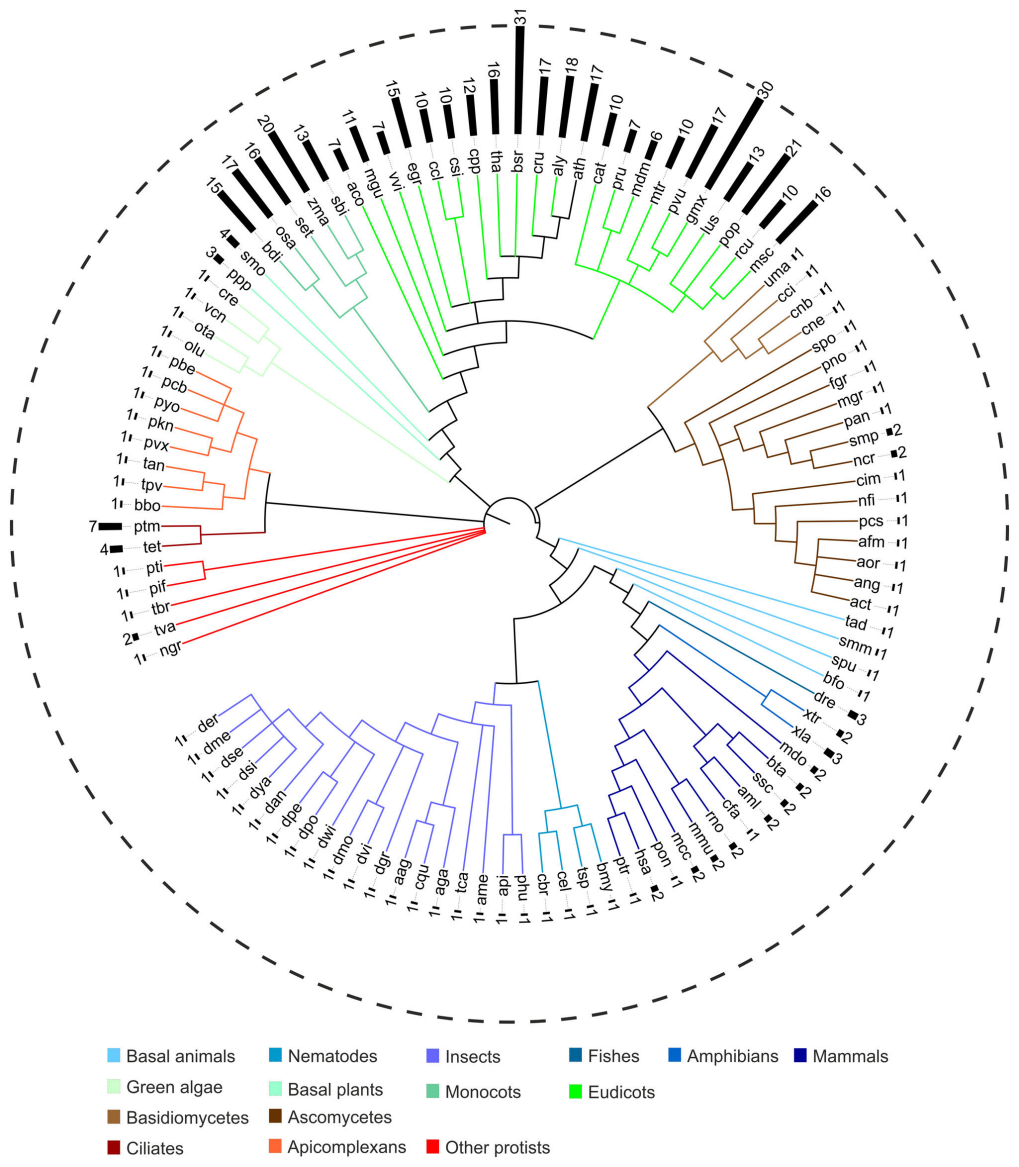
Number of retrieved Rabring7/BCA2/BTLs in eukaryotes. The phylogenetic relationship between thirty-three viridiplantae, forty-one animal, eighteen fungal, and fifteen protist genomes is displayed in a circle. Relationships were adapted from the National Center of Biotechnology Information (NCBI) taxonomy server (http://www.ncbi.nlm.nih.gov/Taxonomy). The color code for major group f organisms is shown at the bottom. The species abbreviations are listed in Table S1 and the genes are listed in Tables S2 and S4.

The number of retrieved *Rabring7*/*BCA2*/*BTL*s from animal, fungi, and protists was much lower than the number of genes retrieved from angiosperms, indicating that this family experienced expansion in the plant lineage (Figure 2 and Table S1). With few exceptions, the genomes of animals, fungi, and protists encoded 1-3 *Rabring7/BCA2* genes. A single gene was detected in genomes from basal forms of animals, including lancelets ( *Branchiostoma* *floridae* ), echinoderms (*Strongylocentrotus purpuratus*), flatworms (*Schistosoma mansoni*), and placozoans ( *Trichoplax* *adhaerens* ). Within mammals, a survey on eleven genomes identified two *Rabring7/BCA2* genes within eight of them (human, rhesus monkey, mouse, rat, giant panda, cow, opossum, and pig), suggesting that two Rabring7/BCA2 genes are encoded in this group. In amphibians, three were annotated in *Xenopus laevis* and two in *Xenopus* *tropicalis* . Similarly, zebrafish encoded three *Rabring7/BCA2* genes. Thus, vertebrates may encode between two and three genes. The nineteen species of insects and four nematodes surveyed contained a single *Rabring7/BCA2*, indicating that invertebrates encode a single gene.

In a survey of fungi species, *Rabring7*/*BCA2* were identified in the two phyla, Ascomycota and Basidiomycota. Of note, within the three Ascomycota subphyla, *Rabring7*/*BCA2* were readily identified in Pezizomycotina and Taphrinomycotina, but not in Saccharomycotina, which included a large number of sequenced genomes (20 in the KEGG database). The observation that *Rabring7*/*BCA2* were not identified in Saccharomycotina was not surprising. A comparison of several Pezizomycotina and Saccharomycotina species revealed that a large number of genes were absent in Saccharomycotina [24]. Rabring7/BCA2 were identified in three major classes of Pezizomycotina (Dothideomycetes, Sordariomycetes, and Eurotiomycetes). A single Rabring7/BCA2 was identified in sixteen out of eighteen fungi species, and two Rabring7/BCA2 genes were present in *Sordaria macrospora* and *Neurospora crassa* (Figure 2), suggesting that two genes are usually present in Sordariomycetes.

A single Rabring7/BCA2 was detected in twelve protist genomes, with the exception of *Trichomonas vaginalis* and the two ciliated genomes surveyed, *Tetrahymena thermophila* and *Paramecium* *tetraurelia* . Two genes were identified in *T*. *vaginales*, seven in *T*. *thermophile*, and four in *P*. *tetraurelia* (Figure 2). A larger number of Rabring7/BCA2 genes in ciliates is not unexpected, since ciliates arose after several whole-genome duplications, and many genes and gene families are found expanded in their genomes [25,26].

The plant genomes included four green algae ( *O* *. lucimarinus* , *O* *. tauri* , *Chlamydomonas reinhardtii*, and *Volvox carteri*), two basal angiosperms (the moss *Physcomitrella* *patens* and the lycopod *Selaginella* *moellendorffii* ), five monocots, and twenty-two eudicot plants (see list of species in Table S1). One BTL member was identified in each of the green algae assessed, three in the moss and four in the lycopod. The number of genes present in the five monocots did not show much variation and ranged from fifteen to twenty members (Figure 2). In eudicots, except for *Brassica* *rapa* and *Glycine max*, which underwent additional whole genome duplications events where each contained at least thirty-one members, the number ranged from six in *Malus* *domestica* to eighteen in *Arabidopsis* *lyrata* . A similar number was found in some related species. For instance, *Malus* *domestica* , *Prunus persica*, and *Cucumis sativus* contained an average of eight members, whereas *Arabidopsis* spp, *Capsella* *rubella* , and *Thellungiella* *halophila* contained an average of seventeen. The larger number of Rabring7/BCA2/BTLs in angiosperm compared to other eukaryotes suggests that this family experienced expansion in the angiosperm lineage.

### Phylogenetic distribution of RING-H2 finger Rabring7/BCA2/BTLs

We performed various types of analyses to generate consistent phylogenies with Rabring7/BCA2/BTLs. We also evaluated phylogenies based on complete protein sequences and on conserved domains, eliminating divergent regions. Trees based on the RING-H2 domain, or trees obtained by concatenating the RING-H2 and the BZF domains, or trees built with complete sequences resulted in similar topology when all species were included. Plants grouped together, separated from the rest of the species; however, the support for this branch classification was not strong (data not shown). Hence, for our phylogenetic analysis, we chose to work with two separated trees, one obtained with 403 plant sequences (the BTL tree) and the other with 99 animal, fungal, and protist sequences (the Rabring7/BCA2 tree). We compared phylogenies generated with the neighbor-joining (NJ), the maximum-parsimony (MP) and the maximum-likelihood (ML) methods that were based on complete sequences or on concatenated RING-H2 and BZF domains (see Materials and Methods).

**Figure 3:**
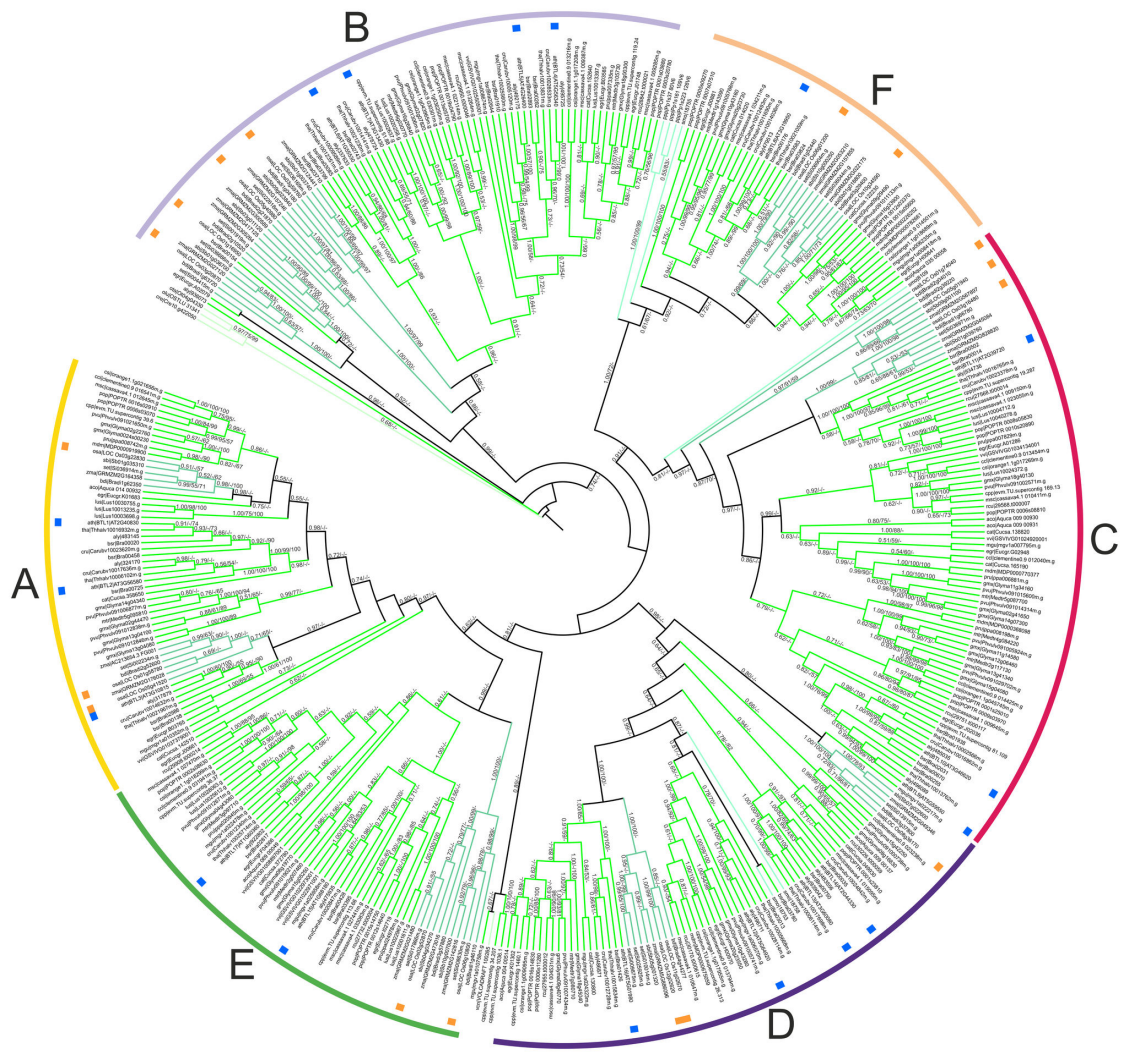
Phylogeny of 403 BTLs proteins from plants. Complete protein sequences and concatenated RING-H2 and the BZF domains were used to obtain the tree; the tree obtained by concatenating domains is displayed in Figure S1. The topology was generated by the ML method; statistical significance in percentages above 50% for NJ, and MP, and posterior probability above 0.5 for ML methods is indicated on the nodes (ML/NJ/MP). The branches from the thirty-three viridiplantae genomes were classified in six groups, A to F.

**Figure 4:**
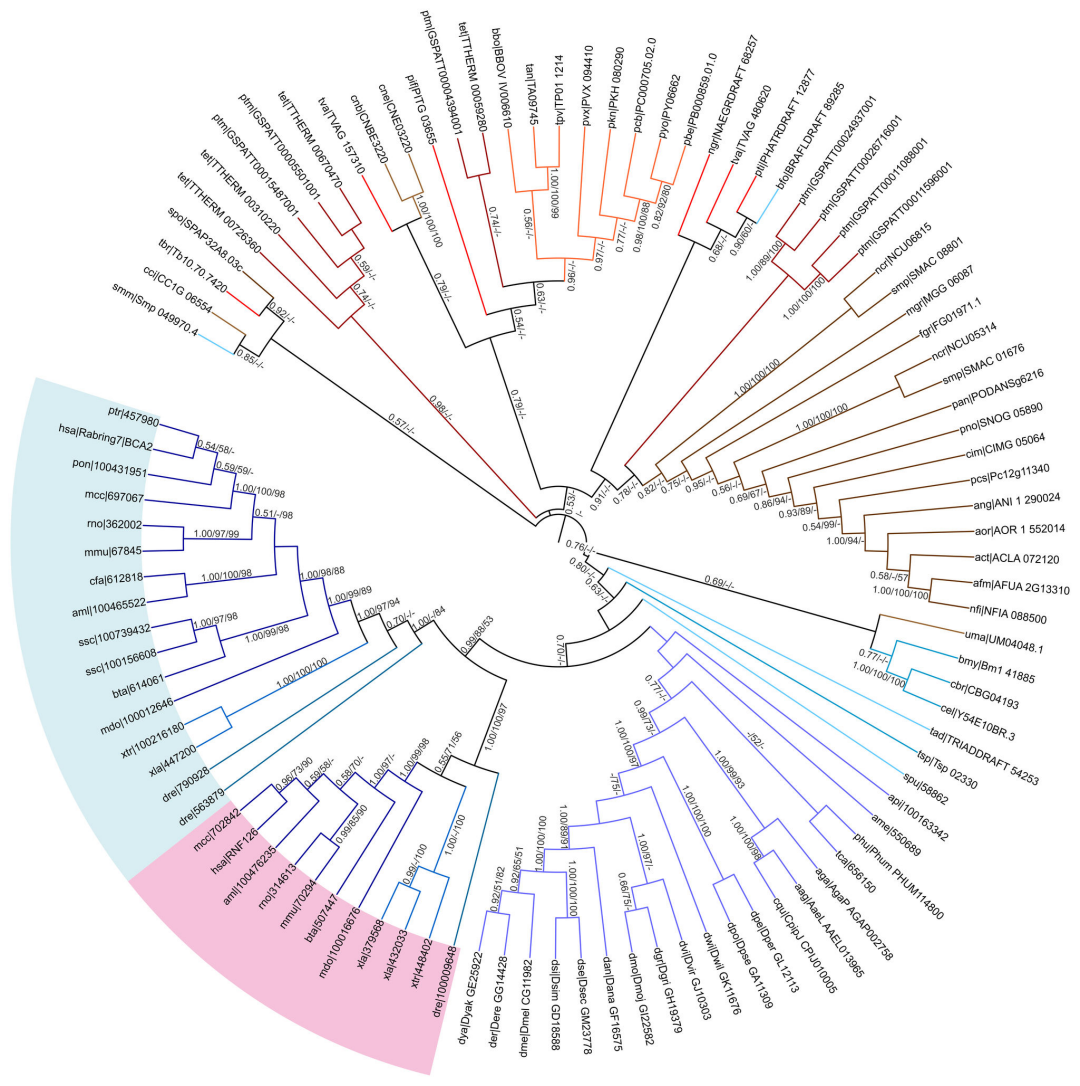
Phylogeny of 99 Rabring7/BCA2 proteins from animals, fungi and protists. The tree was generated as described in Figure 3; the tree obtained by concatenating domains is displayed in Figure S1. The color code on the branches for groups of organisms is that same as in Figure 2.

The BTL and Rabring7/BCA2 phylogenetic trees showed resolution of the species with minor inconsistencies concerning branch distribution; the trees based on complete sequences are shown in Figures 3 and 4, and the trees generated with concatenated RING-H2 and BZF domains in Figure S1. In the BTL trees, chlorophytes were placed in an external basal clade ( *Ostreococcus* *lucimarinus* olu|OSTLU_31341, *Ostreococcus* *tauri* ota|Ot04g04330, *Chlamydomonas reinhardtii* cre|Cre10.g422050, and *Volvox carteri* vcn|VOLCADRAFT100265), though in the tree based on complete sequences one chlorophyte was separated (see vcn|VOLCADRAFT 100265, Figure 3 and Figure S1). Six BTL groups (A to F) were formed on a collapsed branch tree with local support less than 80% (Figure 3). The six groups had clustered members of monocot and eudicot plants, indicating that these members of the family arose before the split of the two major groups of flowering plants. Moreover, all groups included members from the five monocot species. A more detailed description of the groups is shown further on. Few sequences lacked taxonomic congruency. For instance, three BTLs sequences clustered with chlorophytes (see *Arabidopsis* *lyrata* aly|936073, Mimulus guttatus mgu|mgv1a010758m.g and Setaria italica set|Si004415m.g; Figure 3 and Figure S1).

Minor lack of taxonomic congruency was also observed in Rabring7/BCA2 trees (Figure 4 and Figure S1). Basal animal species showed misplacements in both trees. *Strongylocentrotus purpuratus* spu|58862 which is basal for vertebrates came out as basal to all animals in the tree based on complete sequences but was basal for vertebrates in the tree based on concatenated domains. Conversely, *Trichoplax* *adhaerens* tad|TRIADDRAFT 54253 which is basal to animals, came out as basal to all animals in the tree based on complete sequences but basal for vertebrates in the tree based on concatenated domains. Likewise, *Branchiostoma* *floridae* bfo|BRAFLDRAFT 89285, clustered with protist clades and *Schistosoma mansoni* smm|Smp 049970.4 clustered with Basidiomycota species. Among fungi, Basidiomycota species were grouped in the tree based on concatenated domains but were separated in the tree based on complete sequences ( *Coprinopsis* *cinerea* cci|*CC1G* 06554, *Ustilagomaydis* uma|UM04048.1, *Cryptococcus neoformans* JEC21 cne|CNE03220 and *Cryptococcus neoformans* B-3501A cnb|CNBE3220). Also among fungi, *Schizosaccharomyces pombe* spo|SPAP32A8.03c grouped with fungi in the tree based on concatenated domains, but was separated in the complete sequences tree (Figure 4 and Figure S1).

Vertebrates were grouped in a clade consisting of eleven mammalian species, two amphibians, and one fish (Figure 4). They were separated into two branches, one containing putative *Homo sapiens* hsa|Rabring7/BCA2 orthologs, and the other containing putative hsa|RNF126 orthologs (highlighted in blue and pink, respectively, in Figure 4). In seven mammalian species, one member was found in each one of the two branches. An exception was *Sus scrofa* (pig), where the two Rabring7/BCA2 proteins were included in the hsa|Rabring7/BCA2 branch (ssc|100156608, ssc|100156180). It is likely that most mammalians encode two Rabring7/BCA2 orthologs, one related to hsa|Rabring7/BCA2 and the other to hsa|RNF126 (Figure 4). In the two amphibians and the single fish species analyzed (*Xenopus laevis*, *Xenopus* *tropicalis* and *Danio rerio*), two or three Rabring7/BCA2 proteins were identified, respectively. Likewise, they were separated in each one of the branches defined by the mammalian orthologs.

Distinctive clades were resolved for invertebrates. A single clade contained the nineteen insect species and a single clade three nematodes species (*Caenorhabditis elegans*, *Caenorhabditis* *briggsae* and *Brugia malayi*); this particular clade showed different location within both phylogenies (Figure 4 and Figure S1). The two fungal phyla represented by four Basidiomycota species ( *Coprinopsis* *cinerea* , *Ustilagomaydis* , *Cryptococcus neoformans* JEC21 and *Cryptococcus neoformans* B-3501A) and fourteen Ascomycota species were in separated clades (Figure 4 and Figure S1). As mentioned before, in the tree generated with complete sequences the Basidiomycota species were separated (Figure S1). Within Ascomycota, the three Pezizomycotina classes, Dothideomycetes, Sordariomycetes, and Eurotiomycetes, were resolved in sister branches, in the tree based on concatenated domains (Figure S1). The two Rabring7/BCA2 genes that were present in the Sordariomycetes Neurospora *crassa* and *Sordaria macrospora* were also separated (ncr|NCU06815, smp|SMAC08801 and ncr|NCU05314, smp|SMAC01676, respectively). Among protists, several clades were common in both types of phylogenies, suggesting genuine relationships. For instance, apicomplexan and two ciliate proteins defined a branch, and ciliate copies outlined distinct branches as well (Figure 4 and Figure S1).

### PSPM LOGOs to assemble the domain architecture of Rabring7/BCA2/BTL proteins

**Figure 5:**
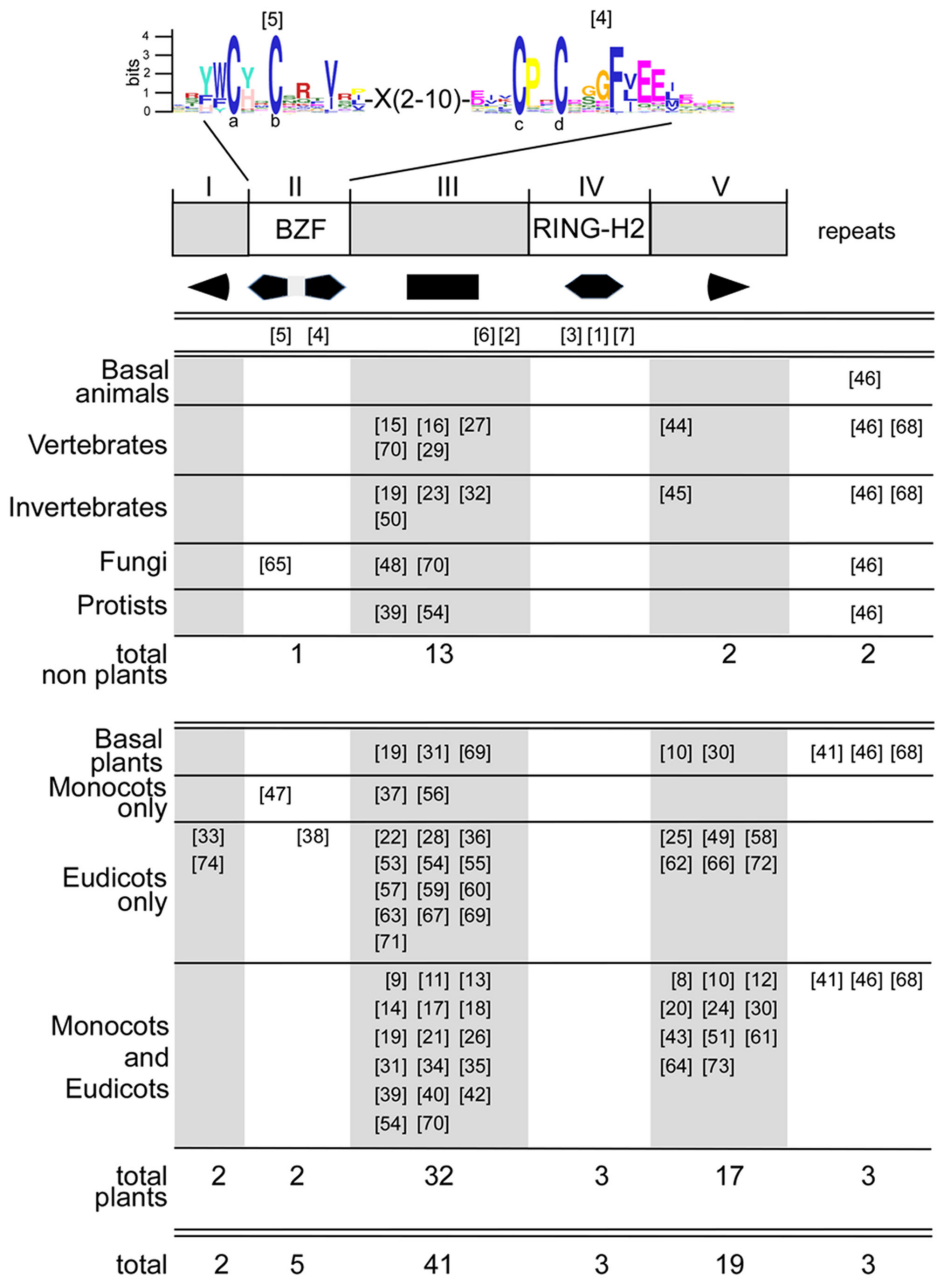
Sequence LOGOs mapped to Rabring7/BCA2/BTLs regions. Below the canonical Rabring7/BCA2/BTLs diagram, geometric figures represent the five modules and the sequence LOGOs mapped to each region for major groups of organisms. Species are arranged into two groups: animals, fungi and protists in one group, and viridiplantae in the other group. The sum of logos in each group is indicated, and the total number of logos is shown at the bottom. The prominent sequence LOGOs for the two zinc fingers is displayed above the diagram; the distance between the pairs of cysteines in 97% of Rabring7/BCA2/BTLs ranges between 11 and 19 amino acids. The catalog of the 73 sequence LOGOs is shown in Table S3.

To obtain a comprehensive domain architecture view of Rabring7/BCA2/BTL proteins for characterization of this family, we divided canonical Rabring7/BCA2/BTLs into five modules using the BZF and RING-H2 zinc fingers as a position reference. These modules are as follows: (I) from the amino-terminal end to the BZF, (II) the BZF (III) between the BZF and RING-H2 domain, (IV) the RING-H2 domain, and (V) from the RING-H2 domain to the carboxy-terminal end (Figure 5). We conducted MEME searches in order to unravel additional motifs and help with the analysis and classification of Rabring7/BCA2/BTLs. Searches were performed using 487 sequences. Seventeen Rabring7/BCA2/BTLs that showed a deviation in the distance between the amino acid residues that characterize the canonical RING-H2 described for this family were not included in the analysis (see Materials and Methods). Based on the PSPM sequence, 73 non-redundant sequence LOGOs that ranged from 10 to 50 residues long were identified. To generate sequence LOGOs, the MAST software package was used (Protein Data Bank, http://www.rcsb.org/pdb/home/home.do). The Interactive Tree Of Life (iTOL) software was instrumental for generating the image of the protein domain architecture using shape and color codes (see Materials and Methods). Each one of the five Rabring7/BCA2/BTL regions was denoted with a single geometric shape, and a different color was used for each sequence LOGO that mapped to a region (Figure 5; the catalog of sequence LOGOs is displayed in Table S3, and they are displayed together with the phylogenetic tree in Figure S1).

Three sequence LOGOs extending along the RING-H2 domain were displayed as a single geometric shape (LOGOs 3, 1, and 7; Figure 5). Two sequence LOGOs that mapped to the BZF were found in most of the species. They were mapped to each cysteine pair, with LOGO 5 of 14 residues long and LOGO 4 of 19 amino acids, respectively long. Taking into account the length of these two LOGOs, the total distance between the two cysteine pairs that was determined for 97% of the Rabring7/BCA2/BTLs ranged from 11 to 19 residues. The sequence LOGO generation revealed conservation and distinctiveness among Rabring7/BCA2/BTLs across eukaryotes. Incidentally, the Lancelets *Branchiostoma* *floridae* (bfo|BRAFLDRAFT_89285) had 6 residues with a minor distance detected. Moreover, distances of up to 26 residues were found in a few eudicots that showed tracks of serine residues between the two pairs of cysteines, as in the *Cucumis sativus* cat|Cucsa.049930 protein (Figure S1). Three additional LOGOS generated within BZF were detected to a lesser extent. LOGO 65 was specifically found in Ascomycetes, LOGO 47 was found in eight proteins from a branch of monocots, and LOGO 38 occurred in five proteins from a branch of Brassicales.

Sixty sequence LOGOs were mapped to regions III and V, 41 to region III, and 19 to region V (Figure 4). In region I, two LOGOs were mapped to closely related proteins from eudicot plants that were grouped in sister branches of the phylogenetic tree (LOGOs 33 and 74, respectively). In addition, three LOGOs were composed mostly of tracks of repeated amino acid sequences consisting of aspartate/glutamate, serines, or alanines (LOGOs 41, 46, and 68, respectively). LOGOs 46 and 68 were found across all eukaryotes scattered along different regions, whereas LOGO 41 was specifically present in plants (Figure 5).

In addition to the sequence LOGOs that mapped to the two zinc finger domains and were conserved across eukaryotes, two LOGOs were also found conserved in almost all species as well: LOGOS 2 and 6 mapped to region III that was adjacent to the RING-H2 domain. Few exceptions were identified, including LOGO 6 in the fission yeast *Schizosaccharomyces pombe* (spo|SPAP32A8.03c), which was not detected under the chosen threshold. Sequence inspection revealed that LOGO 6 is closely related to the sequence of LOGO 3 previously described in ATL E3 ligases (Figure S2A). This LOGO 3 also mapped adjacently to the ATL RING-H2 domain, and defined GLD, which is a motif that is conserved and distinctive of the ATL family. The sequence LOGOs mainly mapped to plant sequences. From 41 LOGOs mapped to region III and 19 to region V, 32 and 17 were found in plants and 13 and 2 in other eukaryotes, respectively (Figure 5), suggesting that diversification of plant BTLs occurred as the family expanded.

Conversely, only two sequence LOGOs were generated on each fungi and protists. Among fungi, Ascomycetes presented distinct domain architecture. Besides LOGO 65, which is specific to this group and includes the first cysteine pair of the BZF (region II, see above), LOGO 48 mapped adjacent to the RING-H2 domain and close to LOGO 2 (see domain architectures on Figure S4). Two sequence LOGOs were identified in protists, suggesting weak sequence conservation among Rabring7/BCA2 genes in this group. Indeed, no common sequence LOGO was detected in the duplicated Rabring7/BCA2 copies of ciliates (see domain architectures on Figure S4).

### Diversity of Rabring7/BCA2-generated sequence LOGOs in animals

**Figure 6:**
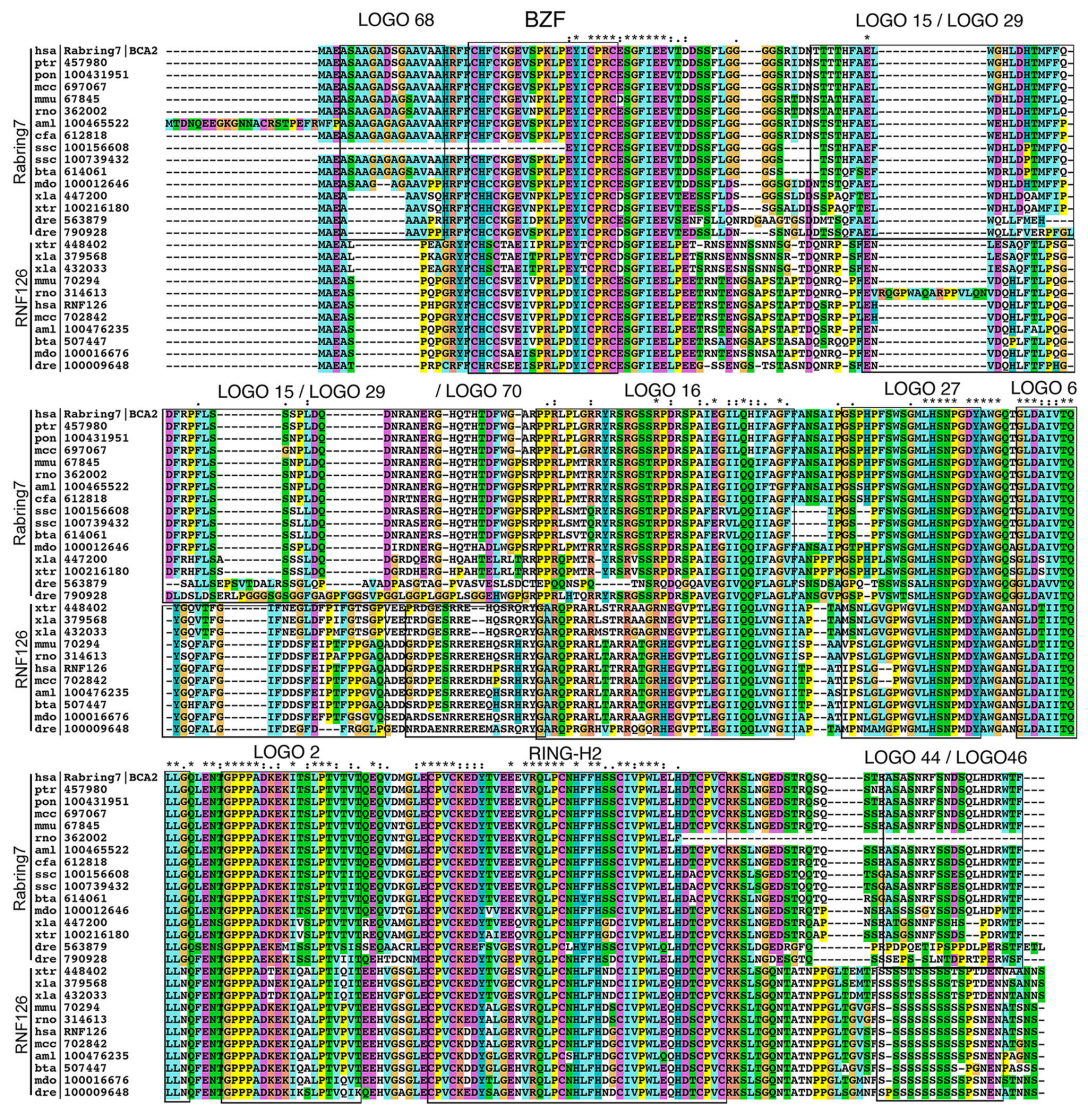
Alignment of the Rabring7/BCA2 proteins from vertebrates. ClustalX 2.0.12 was used for sequence alignment and a default color code was applied. The locations of regions encompassing sequence LOGOs are enclosed by rectangles.

Sequence LOGOs were not generated in basal animal species, but novel sequence LOGOs restricted to distinct groups of animals were readily detected. Among vertebrates, LOGOs 16 and 27 were present in almost all of the species (Figure 6), likewise, LOGOs 6 and 2 which were present in almost all Rabring7/BCA2/BTLs (see above). A sequence comparison revealed that the AKT phosphorylation domain formerly described in the human Rabring7/BCA2 was included within LOGO 16 (Figure 6 and Figure S2B). AKT is a serine/threonine kinase that regulates a diverse array of cellular functions. It has been demonstrated that AKT phosphorylates Rabring7/BCA2 within the AKT phosphorylation site located between the BZF and the RING-H2 zinc fingers [27]. Two Rabring7/BCA2 copies are usually detected in vertebrates [19]. The genes separated in a phylogenetic tree into two sister branches, each one of them grouping as hsa|Rabring7/BCA2 orthologs or hsa|RNF126 orthologs (Figure 6). A distinct domain architecture based on sequence LOGOs was inferred as well. The hsa|Rabring7/BCA2 orthologs exclusively included LOGO 15, LOGO 44 and the sequence repeated LOGO 68, and the hsa|RNF126 orthologs included LOGO 29, LOGO 70 and the sequence repeated LOGO 68, suggesting that they had experienced domain specialization. LOGOs 15 and 44 were not detected under the chosen threshold in the fish copies, suggesting that in those groups the motifs have diverged (dre|790928, and dre|563879) (Figure 6).

**Figure 7:**
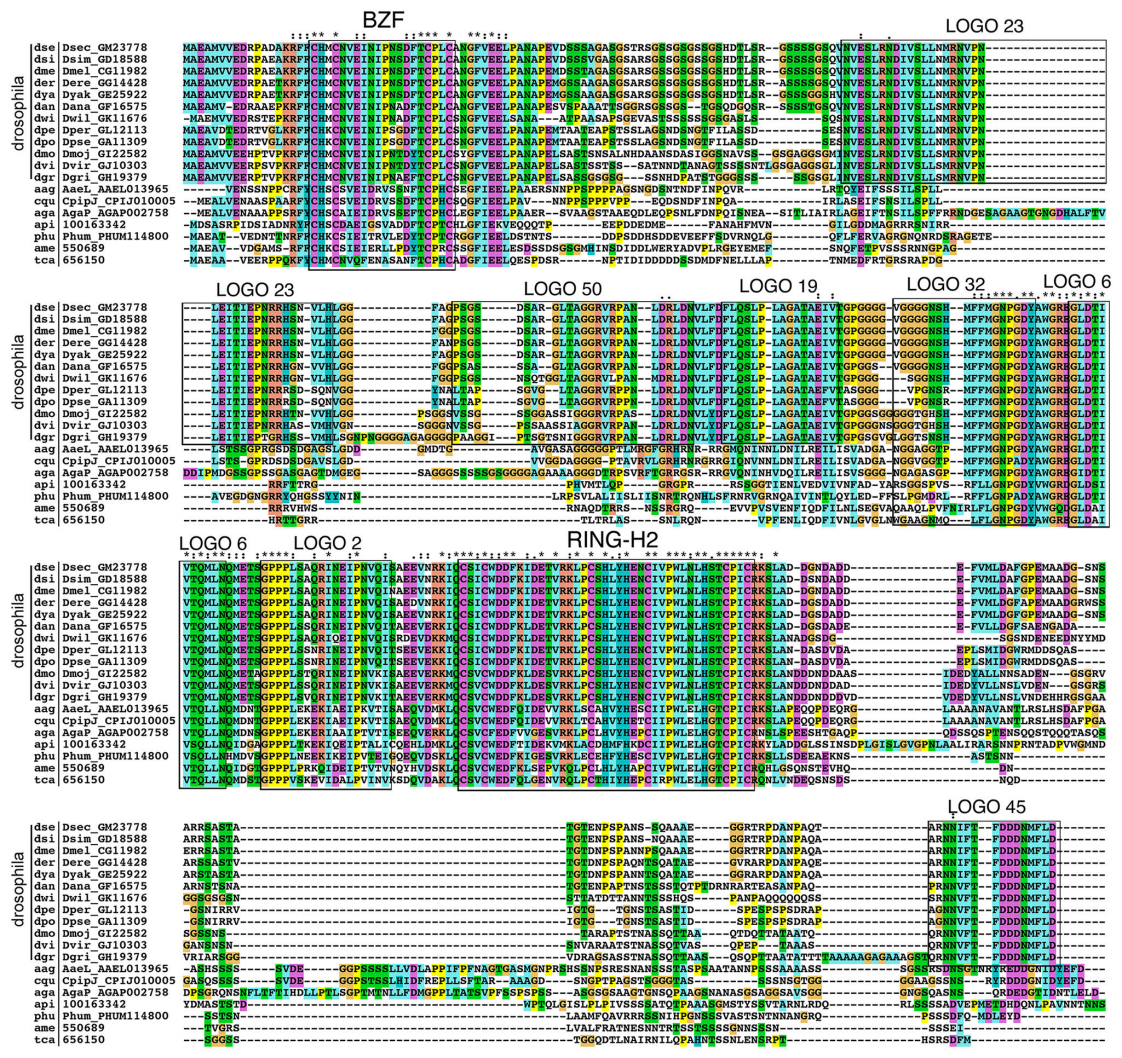
Alignment of the Rabring7/BCA2 proteins from insects. The sequence alignments were performed as described in Figure 6.

Despite that fact that among insects, Drosophila species were overrepresented, they contained unique domain architecture. LOGOs 23, 50, 19, and 45 were present in all twelve Drosophila species and absent or diverge in other insects (Figure 7). Nevertheless, they all shared the common sequence LOGOs present in Rabring7/BCA2/BTLs (LOGOs, 6 and 2) and in invertebrates (LOGO 32).

### Classification and diversification of BTLs in embryophytes

To estimate the diversity in BTL, and to catalog them for future analysis, we inspected the distribution of non-scattered sequence LOGOs in angiosperms. Phylogenetic analysis and BTL-generated pHMM LOGOs were used in order to classify BTLs (Figure 3, Figure S5 and Table S3). This classification was arranged from the tree based on complete sequences that showed coherence with the tree based on concatenated domains in most of the groups; sequences from chlorophytes and basal plants, and sequences that lacked taxonomic congruency were not included in this classification (see Materials and Methods). The distribution of BTLs from 29 angiosperm species in 6 groups is displayed in Table S4 and the domain architecture based on sequence LOGOs is depicted in Figure S5. *A. thaliana* is the prime model and represents a reference plant species. As a reference tool that may help with the family analysis, we numbered the *A. thaliana BTL* paralogs from *BTL1* to *BTL17* (highlighted in yellow, Table S4).

**Figure 8:**
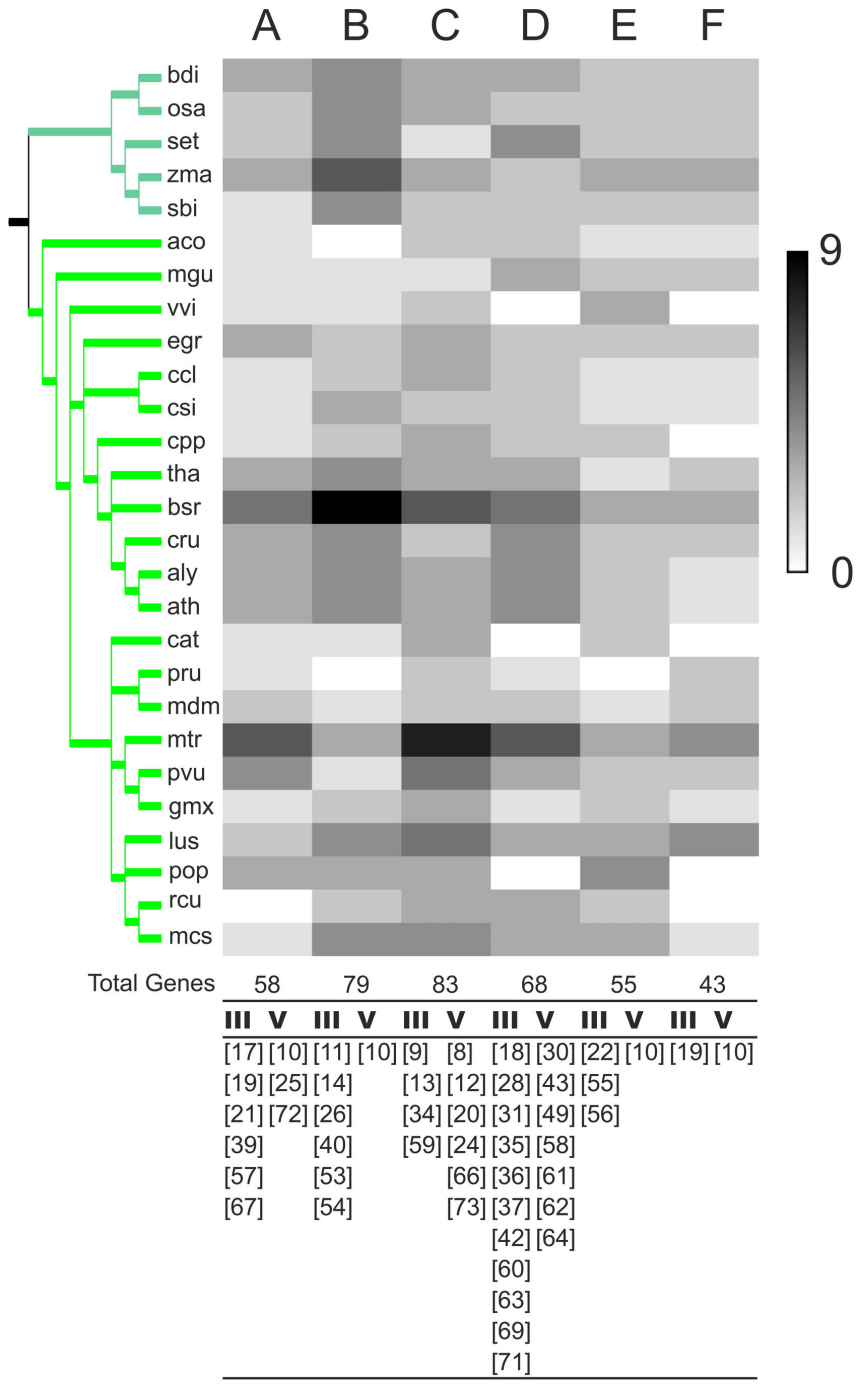
Distribution of BTLs from embryophites in six groups. Heat map representation of the number of BTLs from the 29 species in each one of the six groups by a gray scale. The species tree displayed to the left is adapted from the National Center of Biotechnology Information (NCBI) taxonomy server (http://www.ncbi.nlm.nih.gov/Taxonomy). The total number of genes in each group is shown at the bottom (the catalog of the 396 BTLs in 6 groups in displayed in Table S4). The species tree is the same as in Figure 2. Novel sequence LOGOs mapped to regions III and V on each group are displayed below the heat map; the occurrence for each of these LOGOs is more than 5%.

Approximately 80% of the sequence LOGOs generated on Rabring7/BCA2/BTLs corresponded to embryophites, and most of them mapped to regions III and V: thirty-two LOGOs to region III and 17 to region V (Figure 8). LOGOs 6 and 2 which were common to Rabring7/BCA2 proteins were also present in most BTLs (Figures S3 and S5). Five LOGOs were present in basal embryophytes, and four of them were identified in both monocots and eudicots plants and one only in eudicots, suggesting that they are part of the most ancestral motifs of the plant lineage (LOGOs 19, 31, 10, 30). Similarly, the three LOGOs composed of repeats were found among all embryophytes (LOGOs 41, 46, 68).

Distinct domain architecture based on sequence LOGOs was predicted for each group; an alignment of the members of each group showing the location of sequence LOGOs is displayed on Figure S3. In general, novel and specific LOGOs were found in the six groups and generated prevalent domain architectures in each of them (Figure 8 and Figure S3). However, few redundant LOGOs were identified; for instance, LOGO 10, which mapped to region V, was present in members of group A, B, E, and F (Figure 8 and Figure S3). A motif that was previously identified was also detected among the sequence LOGOs. The domain-of-unknown-function (DUF) 1117 located at the carboxy terminal end of ath|BTL9/AtRDUF2 and ath|BTL10/AtRDUF1 was found encompassed by LOGOs 8, 12, 20, and 24 [22] (Figure S2C). These LOGOs were present across angiosperms in members of group C (Figure 8 and Figure S3).

### Tandemly Arrayed BTL genes

Tandem gene duplication plays an important role during the expansion and diversification of vascular plants [1,28]. Moreover, clusters of tandemly arrayed genes frequently occur in members of the ATL family [8]. Arrays of two to five genes are commonly found, and arrays up to nine genes have been identified. Similarly, the percentage of tandemly arrayed ATLs per genome also varies, and is usually between 2 and 6%, but can be greater than 20% in some species [8]. To estimate the importance of tandem gene duplication on the BTL family structure and evolution, we searched for tandem clusters in twenty-nine embryophite genomes. The tandem arrays were recognized based on the locus name and by examination of the assembly of the genes on the chromosomes and/or scaffolds in the Gbrowse of the Phytozome database. Four tandemly arrayed genes were identified, each consisting of two genes (genes are shadowed in gray in Table S4). Two arrays occurred in the family Fabaceae (*Glycine max* and *Phaseolus vulgaris*), which may correspond to a pair of orthologous genes, since they are found in the same BTL group (Table S4). The other two arrays were identified in *Aquilegia* *coerulea* and *Vitis vinifera*, which are two species that contain a reduced number of BTL genes (Figure 2). The fact that tandem gene arrays were infrequently found suggests that tandemly duplicated BTLs are not usually fixed in vascular plant genomes and may not play an important role in the expansion of this family in plants.

### Potential Protein-Protein Interactions taking place on BTLs

**Figure 9:**
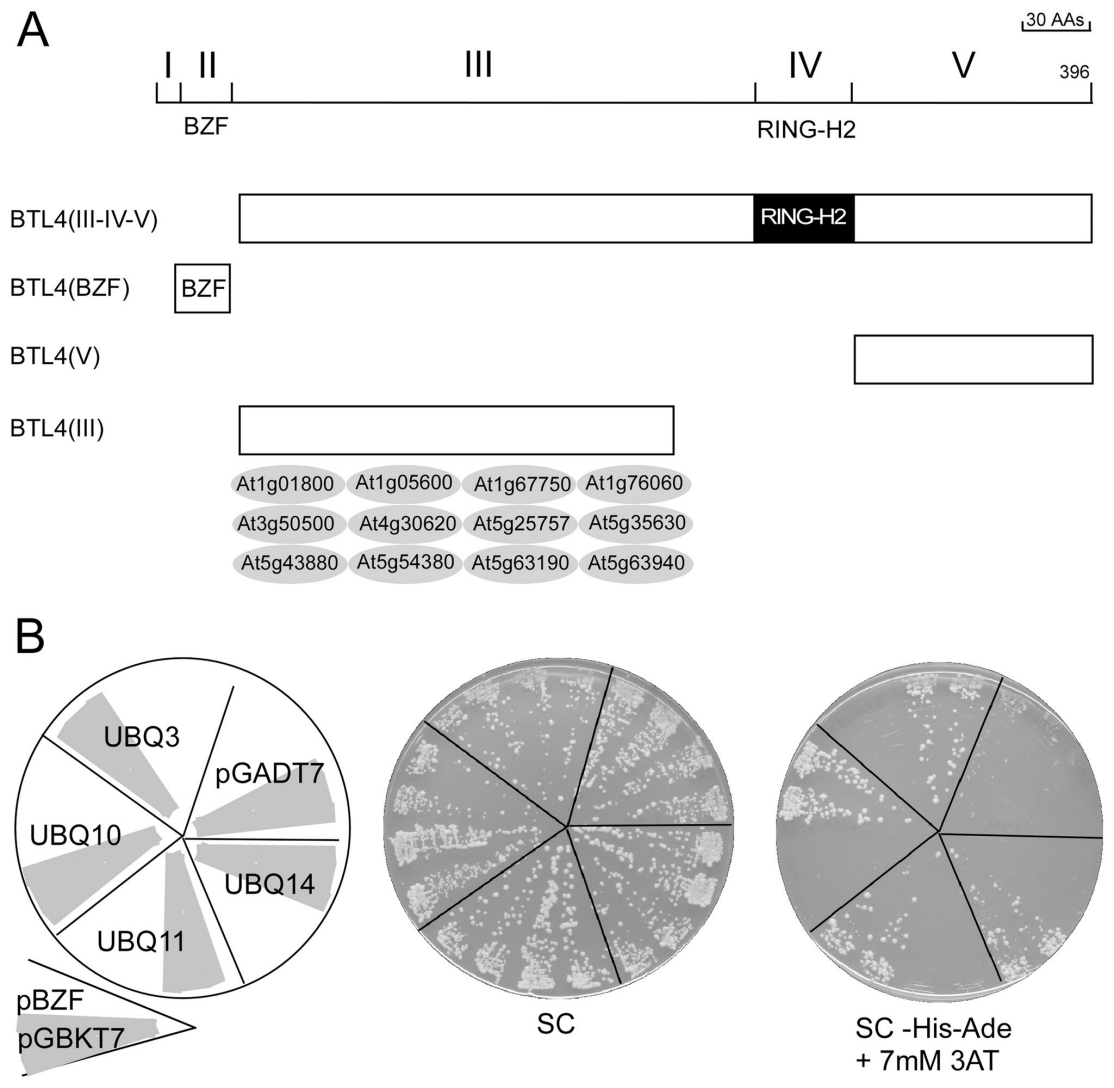
Protein–protein interaction domains in BTLs. (A) The upper diagram depicts the modular organization of ath|BTL4 based on sequence LOGOs. The lower diagram is a schematic representation of four clones encompassing different regions of ath|BTL4 that were used for the yeast two-hybrid screening and assays. Twelve clones depicted below the clone BTL(III) within a gray circle were retrieved from a yeast two-hybrid screening using the BTL4(III-IV–V). The segment in BTL4 that mediates the interaction with all of them was further mapped to region III by a yeast two-hybrid assay; the description of the interactors is provided in Table S5. (B) Interaction between BZF and ubiquitin. Representative plates showing yeast two-hybrid interactions between BTL4(BZF) and four *A. thaliana* ubiquitin clones. The left panel shows the template of the plates. The BTL4 BZF-containing fragment was ligated into the DNA-binding domain of pGBKT7, and the ubiquitin clones are in pGAD10, which is a pGADT7-related vector. The yeast strain AH109 was cotransformed with pGBKT7 and pGADT7 derivatives, selecting for transformants on SC lacking Trp and Leu. Two representative transformants were then streaked onto SC medium lacking Trp and Leu (SC) and onto SC medium with 7 mM 3-AT and lacking Trp, Leu, His, and Ade. The plates were incubated at 30^o^C for four days; growth is observed as dense streaks of yeast over background. The interaction with pGBKT7 was included as a negative control (depicted as a gray sector in the template plate).

Defining regions that mediate protein–protein interactions is essential for analyzing E3 ligases [3]. Rabring7/BCA2/BTLs may be modular RING finger E3 ligases that encode domains for recognition of the substrate and the E2 ubiquitin-conjugating enzyme. They may also encode domains, such as the BZF, that may support the function of the ubiquitin ligase. To identify possible interacting domains in BTLs, we conducted yeast two-hybrid screens. We selected ath|BTL4 as a representative member of the family, since it is conserved across all angiosperms tested. According to our classification, ath|BTL4 belongs in group B (Table S4). We obtained twelve positive clones from our screen using BTL4(III-IV–V) (Figure 9A). We then approximated the location of the region on BTL4 involved in the interaction. All twelve clones showed interaction with region III (Figure 9A), suggesting that the region between the two zinc fingers harbor domains that mediate protein–protein interactions.

BZF, the Rabring7/BCA2 zinc finger domain located at the NH2 terminus, interacts with ubiquitin and ubiquitinated proteins [14]. The interaction between Rabring7/BCA2 and ubiquitin was previously discovered through a bacterial two-hybrid screen, and the domain mediating the interaction within Rabring7/BCA2 was subsequently mapped to the BZF [14]. To explore whether the BZF-like domain present in BTL will bind ubiquitin, we employed a yeast two-hybrid system. The interaction between a fragment containing the BZF domain from ath|BTL4 and four ubiquitin clones was assayed. We found that the BZF-like domain can mediate the interaction with AtUBQ3, AtUBQ10, AtUBQ11, and AtUBQ14 (Figure 9B).

### Prevalence of spliceosomal introns in Rabring7/BCA2/BTL genes

An analysis of gene architecture indicated that spliceosomal introns within the coding DNA sequence (CDS) are absent in *A. thaliana* and *O. sativa BTLs*. This feature is shared by *ATLs*, on which approximately 90% of all the genes lack introns. Among plants, a CDS-intron was only predicted in green algae [8]. To identify trends that may contribute to a better understanding of the emerging plant BTLs, we contrasted the occurrence of spliceosomal introns within the CDS across eukaryotes. In contrast to plant *BTLs*, introns within the coding sequence of *Rabring7/BCA2* genes from animal, fungi, and protists were present in most organisms (Table 1).

**Table 1:** Spliceosomal Introns at the Coding DNA Sequence of Rabring7/BCA2/BTL Genes.

			**Introns within CDS**
Plants				
	Green algae		
		Prasinophyceans	1
		Chlorophyceae	5-10
	Ferns		0
	Mosses		0
	Monocots		0
	Eudicots		0
Animals			
	Vertebrates		
		Mammals	8-9
		Amphibians	6-8
		Fishes	8
	Lancelets		3
	Echinoderms		8
	Insects		
		Culicidae	3-4
		Apidae	7
		Aphididae	7
		Pediculidae	6
		Tenebrionidae	4
		Drosophilidae	0
	Nematodes		2-4
	Placozoans		8
Fungi			
	Basidiomycetes		
		Tremellomycetes	10
		Agaricomycetes	9
		Ustilagomycetes	0
	Ascomycetes		
		Eurotiomycetes	2-4
		Sordariomycetes:	
		-Hypocreales	7
		-Magnaporthales	3
		-Sordariales	0-2^1^
		Dothideomycetes	2
		Taphrinomycetes	1
Protists			
	Apicomplexans		0-2^2^
	Ciliates		0-1^3^
	Parabasalids		0
	Amoeboflagellate		0
	Diatoms		2
	Kinetoplasts		0
	Oomycetes		5

The taxonomic ranks of the hierarchy of biological classification within columns are not the same for every group of organisms.

^1^ smp|SMAC_08801and ncr|NCU06815 are intronless genes and smp|SMAC_01676 and ncr|NCU05314 contain introns.

^2^ pbe|PB000859.01.0, pcb|PC000705.02.0, pkn|PKH_080290, pvx|PVX_094410 and pyo|PY06662 are intronless genes and bbo|BBOV_IV006610, tan|TA09745 andtpv|TP01_1214 contain introns.

^3^ ptm|GSPATT00005501001, ptm|GSPATT00011088001, ptm|GSPATT00011596001, ptm|GSPATT00015487001, ptm|GSPATT00024937001, ptm|GSPATT00026716001, tet|TTHERM_00310220, tet|TTHERM_00670470 and tet|TTHERM_00726360 are intronless genes and ptm|GSPATT00004394001 and tet|TTHERM_00059280 contain introns.

Between two and nine introns were predicted in animals. One exception was evident among members of the family Drosophilidae. Insects encode a single *Rabring7*/*BCA2* gene containing from three to seven introns (Table 1), but in the case of the *Rabring7*/*BCA2* gene from the twelve Drosophila species analyzed, all were intronless genes. It is possible that during the evolution of the Drosophilidae family the *Rabring7*/*BCA2* gene was duplicated by retroposition that was generated by reverse transcription of mRNA [29]. Similar cases may have occurred in fungi and protists, and up to 10 introns were predicted in fungi. Basidiomycetes contained nine or ten introns, and in the Ustilagomycetes *Ustilagomaydis* , the *Rabring7*/*BCA2* ortholog (uma|UM04048.1) was an intronless gene. The uma|UM04048.1 might be a pseudogene generated by retroposition, since it contained an insertion that added twenty amino acid residues between the second and third residues involved in zinc ligation within the RING-H2 domain, which presumably render a defective enzyme. Among fungi, almost all species encoded a single *Rabring*7/*BCA2*. Two species from the order Sordariales, *Sordaria macrospora* and *Neurospora crassa*, contained two *Rabring*7/*BCA2* genes (Table 1). The gene architecture revealed that in these two species, one *Rabring*7/*BCA2* contained two introns and the other was an intronless gene; these may also be situations of duplication by retroposition. Most protist *Rabring*7/*BCA2* genes contained introns (Table 1). The apicomplexan species may or may not have introns, and in ciliates, which contained several copies, one copy had introns whereas the others did not. In those cases it is also possible that the occurrence of intronless genes is the result of retroposition duplication events.

### Spliceosomal introns located at untranslated regions (UTR) are evolutionary conserved in BTLs

During our analysis of the gene architecture of BTLs, we noticed that nine out of the seventeen *A. thaliana BTL* genes had introns at their UTRs: eight at the 5’ UTR and one at the 3’ UTR. Spliceosomal introns are transcribed into pre-mRNA and promptly processed during splicing to produce a mature mRNA. The 5’ UTR spliceosomal introns are usually longer than introns within coding sequences and can harbor regulatory elements that may promote an increase in gene expression or mRNA stability. The 3’ UTR introns occur less frequently than the 5’ UTR introns, and in some cases they down-regulate the level of gene expression [30].

To assess the importance of the UTR spliceosomal introns, we analyzed the evolutionary conservation by contrasting BTLs from *A. thaliana* and *O. sativa*, which are representative species from eudicots and monocots, respectively. The corresponding cDNA clones for most of the predicted genes from these species have been isolated. Seventeen BTLs were identified in *O. sativa*, twelve of which contained introns located at the UTRs: nine at the 5’ UTR, five at the 3’ UTR, and in two BTLS, introns were located in both UTRs (Table 2). The BTL groups supported by phylogenetic analysis contained members of both *A. thaliana* and *O. sativa* (Figure 8 and Table S4). Almost all of the genes from groups A and B contained an intron at the 5’ UTR, and all genes from groups C and E were intronless genes. A similar number of members from each species were present in each one of these four groups, suggesting that the occurrence of introns at the 5’ UTR is conserved by lineage. Conversely, 3’ UTR introns were not found unique in any of the groups, and the six BTL genes that contained 3’ UTR introns were distributed in four groups.

**Table 2:** Spliceosomal Introns at Untranslated Regions (UTR) of BTLs.

**Group**	**BTL gene**	**Intron location**	**Interon length (nt)**	**cDNA clone**
A	ath|BTL1/RHC1a	5’UTR	741	R24044
	ath|BTL2	5’UTR	792	R10352
	ath|BTL3	5’UTR	675	R24260
	osa|LOC_Os03g22830	5’UTR	821	J023049J20
	osa|LOC_Os05g41520	intronless	-	OSIGCSN035B04
	osa|LOC_Os01g58780	5’UTR	562	J033125G22
B	ath|BTL4	5’UTR	463	R11278
	ath|BTL5	5’UTR	337	R25506
	ath|BTL6	5’UTR	453	R14449
	ath|BTL7	5’UTR	299	BX837858
	osa|LOC_Os05g40980	5’UTR	764, 31	J013096K09
	osa|LOC_Os03g59760	5’UTR; 3’UTR	551; 109	J013111A16
	osa|LOC_Os03g20870	5’UTR	322	J013135G08
	osa|LOC_Os01g16950	5’UTR	420	J023108I11
C	ath|BTL9/AtRDUF2	intronless	-	R13790
	ath|BTL10/AtRDUF1	intronless	-	R24103
	ath|BTL11	intronless	-	R21507
	osa|LOC_Os03g16480	intronless	-	J023018C07
	osa|LOC_Os05g01940	intronless	-	J013094N21
	osa|LOC_Os01g74040	intronless	-	J065019N08
D	ath|BTL12	intronless	-	R16110
	ath|BTL13	intronless	-	BX822831
	ath|BTL14	intronless	-	NA
	ath|BTL16	5’UTR	126	BX832364
	osa|LOC_Os11g02670	5’UTR; 3’UTR	113; 920, 95	J033119L05
	osa|LOC_Os12g02620	5’UTR	113	J033073H23
	osa|LOC_Os08g36170	3’UTR	4332	R21874
E	ath|BTL15	intronless	-	NA
	ath|BTL17	intronless	-	NA
	osa|LOC_Os06g10800	intronless	-	NA
	osa|LOC_Os02g52870	3’UTR	93	OSIGCSA056L07
F	ath|BTL8	3’UTR	1143	R21874
	osa|LOC_Os10g34590	3’UTR	2123	J033094G14
	osa|LOC_Os06g01200	5’UTR	2855	J033105E24

## Discussion

In this study, we described a family of RING-H2 E3 ligases, named Rabring7/BCA2/BTL, which has members across diverse eukaryotic species. We integrated the resources of 33 viridiplantae, 41 animal, 18 fungi, and 15 protist genomes and defined common and unique features among *Rabring7*/*BCA2*/*BTL* genes that may be important for future analysis. One hallmark of the Rabring7/BCA2/BTL family is the presence of a C2/C2 zinc finger, named BZF, together with the RING-H2 domain in the same protein. The sequential arrangement of these two zinc fingers across eukaryotes suggests that they are evolutionarily related by homology and that they are likely to exhibit a similar mechanism of action. The fact that unique sequence LOGOs were generated from the RING-H2 and the BZF domains of most of the retrieved protein sequences across several eukaryotic species supports the notion that *Rabring7/BCA2/BTL* have a common origin (Figure 5).

Our previous analysis of the ATL family of RING-H2 E3 ligases was an informative reference to the study on *Rabring7*/*BCA2/BTL* genes [8]. Although the primary sequence of the RING-H2 domain is highly similar, the overall protein domain structure differs between them (Figure 1). A key difference is the BZF that is common to Rabring7/BCA2/BTLs as well as one or more transmembrane domains predicted in ATLs. Similarly, the inferred location of the domains for substrate recognition may be different in these two classes of E3 ligases since most of the yeast two-hybrid interactors of ATLs mapped toward the carboxy-terminal end [8], and all of the yeast two-hybrid interactors of BTLs mapped between the two zinc fingers (region III; Figure 6). The overall conservation in spacing between cysteines and histidines residues involved in zinc ligation suggests functional constraints on the RING-H2 domain. Moreover, a constraint related to the spacing between the second and third cysteines is exclusively preserved in each of the members of these two families (Figure 1); however, the structural implication of these constraints has not been established. Furthermore, a direct correlation exists between the co-occurrence of transmembrane helices or BZFs with an ATL RING-type or a Rabring7/BCA2/BTL RING-type of domains, respectively. Of note, in two species from the green algae class Prasinophyceae, the spacing between the second and third cysteines resembled the canonical spacing found in ATLs (Figure 1). This disposition is not found in algae from the class Chlorophyceae or in any other of the retrieved Rabring7/BCA2/BTL proteins. The fact that ATLs were not previously identified in green algae makes the evolution of these classes of E3 ligases in plants puzzling.

With few exceptions, *Rabring7*/*BCA2*/*BTLs* are found throughout the eukaryotic tree of life, whereas *ATLs* evolved specifically in land plants (Embryophyta). Nevertheless, due to the incomplete state of several genome sequences or their unfinished gene annotation, the occurrence or the actual number of *Rabring7/BCA2/BTLs* is probably underestimated in some cases. One to three *Rabring7*/*BCA2/BTL* genes are found in protists (except for ciliates), fungi, and animals, whereas up to thirty-one were retrieved from angiosperms (Figure 2). Therefore, gene duplication and whole genome duplication may have played important roles in the evolution and expansion of *Rabring7*/*BCA2/BTL* genes in plants because it has been found to occur in several families [28].

The expansion of a gene family in plants also often correlates with tandemly arrayed genes. Tandemly arrayed *ATLs* are common in most plants, including basal species, showing different rates of duplication that range from 3% to 36%, depending on the species. The number of genes in the array fluctuated from two arrayed genes to more than 20 genes. Conversely, tandemly arrayed *BTLs* were scarce, and only three arrays with a single duplication were identified. It is possible to speculate that this contrasting mode of gene duplication may denote the different basic function of these two types of E3 ligases and its effect on plant fitness. Considering that *Rabring7/BCA2/BTLs* are conserved throughout evolution, since they are present across most eukaryotic species, and therefore may be essential for all eukaryotes [31]. Duplication events of BTLs may not be maintained because they will perturb the gene function. In contrast, ATLs may freely evolve because they are under less constraint than BTLs without duplicated genes exerting deleterious effects on fitness [32,33]. In addition, it has been proposed that ATLs play a role during plant adaptation to changing environmental conditions, and thus tandem duplication of ATLs is beneficial, resulting in an advantage for survival [8].

Common sequence LOGOs across all or most eukaryotic organisms were identified, which supports the hypothesis that *Rabring7*/*BCA2*/*BTLs* have arisen from a common ancestor. Eight sequence LOGOs mapped to the two zinc finger domains, and two additional LOGOs were also found to be common to almost all species (LOGOs 2 and 6; Figure 5). LOGO 6 resembles the GLD motif, which was previously described for the ATL family of E3 ligases and may be important for proper functioning of the RING ligase [8] (Figure S2A). Novel LOGOs occur mostly in embryophytes, where the number of LOGOs in basal species is scarce and greatly increased among angiosperms. These findings indicate that plant *BTL* genes experienced expansion and diversification in superior plants. This increase in LOGO occurrence is also an indication of novelties in embryophytes and may be regarded as the acquisition of new motifs.

The occurrence and density of spliceosomal introns within the coding sequence of *Rabring7*/*BCA2*/*BTLs* differs among distant lineages. In general, animal, fungus, and protist genes contain introns, whereas plant genes lack introns. A peculiar observation is the absence of introns in the family Drosophilidae (Table 1). Assuming that in this case the occurrence of these intronless genes is the result of retroposition-mediated duplication generated by reverse transcription of mRNA during Drosophila evolution, the duplicated copy became fixed and shifted with the original copy. This intronless copy was presumably inserted in a transcriptionally active region of the genome appropriate for its expression. Examples where a gene, presumably generated by retroposition, displaced the source gene in evolution have been documented. The *e*(*y*) *2* and *e*(*y*) *2b* genes of *D. melanogaster* encode co-activators of RNA polymerase II that are conserved in evolution. The *e*(*y*) *2b* gene is a paralog of the *e*(*y*) *2* gene that originated by retroposition of *e*(*y*) *2b*. The *e*(*y*) *2* retrogene is broadly distributed and has a general function, while *e*(*y*) *2* only functions in a few specific cells [34]. Genes duplicated by retroposition may promptly acquire novel functions after duplication that are important for adaptation. Remarkably, various sequence LOGOs generated with Rabring7/BCA2 proteins are unique to the genus Drosophila, suggesting that they correspond to distinct motifs acquired in this group (LOGOs 23, 50, 19, and 45; Figure S1 and Table S2). The finding of fungi and protist cases that may be viewed as retroposition events (Table 1) suggest that BTLs present in land plants may have originated by retroposition and then underwent subsequent expansion. Alternatively, a cDNA-mediated homologous recombination mechanism triggered by double-strand-break repair machinery may be responsible for intron loss [35].

Novelties were also detected in BTL gene architecture. Although plant BTLs are intronless genes, many of them have spliceosomal introns located at UTRs, primarily at the 5’ UTR. This 5’ UTR intron arose early in BTL evolution as it was detected in lineages that included both monocot and eudicot representative species. The 5’ UTR spliceosomal introns are often found in plant genes and encode regulatory elements that enhance gene expression [36,37]; it is likely that in several BTLs, 5’ UTR introns may be important for gene expression. Assuming that BTLs in land plants originated by retroposition, the 5’ UTR intron may have been acquired in an early duplication event and preserved in many BTLs during the evolution.

Related domain architecture may be common to all Rabring7/BCA2/BTL proteins. All proteins contain a RING-H2 zinc finger, which is postulated to provide E3 ligase activity, and the hypothesized interaction between BZF and ubiquitin may play a role in Rabring7/BCA2/BTLs stability [15]. In addition to the two representative domains, region III may participate in substrate recognition or in interactions with elements that facilitate the function of BTLs. Sequence LOGO information revealed that most of the generated LOGOs mapped to region III, and that an exclusive LOGO set mapping to this region was characteristic for each BTL group. These results suggest that this region plays a central role in the functional diversity of BTLs. The fact that region III mediates the interaction with all of the interactors identified by the yeast two-hybrid screens supports these assumptions. Indeed, 14-3-3sigma protein, a substrate of BCA2, binds to the corresponding region III [15]. Conversely, region V may have a general function, since a sequence LOGO mapped to region V was frequently found in members of four BTL groups (LOGO 10; Figure 8 and Figure S3).

Results from *A. thaliana* suggest that BTL12/CIP8 forms an E3 complex with the E2 conjugase UBC8 and with COP1, a RING finger ligase. Accordingly, BTL12/CIP8 assists in the ubiquitination of target proteins [21]. A model for BCA2 function that might be conserved in Rabring7/BCA2/BTLs has been proposed [15]. Rabring7/BCA2 is highly unstable and is autoubiquitinated on two acceptor lysines present in the BZF. Although there is currently no parallel evidence on the mode of action of Rabring7/BCA2/BTLs in plants and animals, the fact that two canonical domains are preserved within them suggests that Rabring7/BCA2/BTLs use a similar mechanism of action. Characterization of Rabring7/BCA2/BTL E3 ligases is in its early stages. The functional analysis of members of this family together with he identification of substrates may reveal their role and the mode of action of these conserved components of the UPS across eukaryotic organisms.

## Materials and Methods

### Identification and Retrieval of Rabring7/BCA2/BTLs

The viridiplantae sequences analyzed in this study were retrieved from the genomes deposited in the Phytozome 8 database at http://www.phytozome.net/, and the sequences from animal, fungus, and protist genomes were retrieved from the Kyoto Encyclopedia of Genes and Genomes (KEGG) at http://www.genome.jp/kegg/. The viridiplantae genomes included 4 chlorophyte, 2 basal plants, 5 monocots, and 22 eudicot plants. Other eukaryotic genomes included 4 basal animals, 11 mammals, 2 amphibians, 1 fish, 19 insects, 4 nematodes, 18 fungi, and 15 protists (species are listed in Table S1). When truncated sequences were readily assembled into *Rabring7/BCA2/BTL*-like genes by visual inspection, they were included in the list.

A Rabring7/BCA2/BTL RING-H2 and BZF Hidden Markov Models (HMM) were constructed and calibrated from the four *A. thaliana* protein sequences and Rabring7/BCA2. The Rabring7/BCA2/BTL RING-H2 consisted of a forty-one amino acid sequence flanked by the first and eighth cysteine residues involved in metal ligation: CAVCKDDFEIGSEAKEMPCKHIYHSDCIVPWLELHNSCPVC. With this model, a total of 2356 non-redundant sequences were retrieved. We then queried the BZF model, which consisted of the 30-42 amino acid sequence ARYWCHMCSQMVNP-x(3,12)-IKCPFCQGGFVEE, and retrieved 502 sequences. These 502 were designated as *Rabring7*/*BCA2* when derived from animal, fungus, and protist genomes or *BTL* when derived from plant genomes. *Rabring7/BCA2* and *BTL* genes were designed based on the KEGG Organism Code at http://www.genome.jp/kegg/kegg3.html, and as previously described. In *A. thaliana* we numbered the genes *BTL1* to *BTL17* (see Table S4).

Some proteins displaying non-canonical distances between the residues that define the RING-H2 domain were included in the analysis as well, since it was inferred that a BZF-like domain was present. Two Basidiomycetes showed insertions that render twenty and six amino acid residues insertions, respectively, between the second and third residues involved in zinc ligation within the RING-H2 domain ( *Ustilagomaydis* , uma|UM04048.1 and *Coprinopsis* *cinerea* , cci|CC1G_06554). Two green algae, ( *Ostreococcus* *lucimarinus* , olu|OSTU_31341 and *Ostreococcus* *tauri* , ota|Ot04g04330), displayed an ATL-like RING-H2 domain. In three plant species, one to four amino acid residue insertions were detected between the second and third residues (*Zea mays*, zma|GRMZM2G164358; *Brassica* *rapa* , 2 bsr|Bra00282 and *Mimulus* *guttatus* , 4 mgu|mgv1a010758m.g), and two plant species had a single residue present between the sixth and seventh residues (*Manihot esculenta*, msc|cassava4.1_015095m.g and *Ricinus communis*, rcu|30226.t000059). In a group of apicomplexan protist proteins, insertions that resulted in four or five additional amino acids were also included (*Plasmodium berghei*, pbe|PB000859.01.0; *Plasmodium chabaudi*, pcb|PC000705.02.0; *Plasmodium* *yoelii* , pyo|PY06662; *Plasmodium* *knowlesi* , pkn|PKH_080290; *Plasmodium vivax*, pvx|PVX_094410; *Theileria annulata*, tan|TA09745; *Theileria parva*, tpv|TP01_1214 and *Babesia bovis*, bbo|BBOV_IV006610).

#### Phylogenetic analyses, alignments, and model test for sequences

Basic bioinformatic procedures were essentially performed as previously described [8]. To obtain Rabring7/BCA2/BTL phylogenetic trees based on high alignment quality support, trees were generated with complete protein sequences. The 502 retrieved sequence (or the 403 plant and the 99 animal, fungi and protists) were aligned using MUSCLE version 3.8.31 [38] and estimated the amino acid substitution model best fitting the BTL alignment using ProtTest [39]. In the phylogenetic analysis, Neighbor-joining (NJ) and Maximun-parsimony (MP) trees were obtained using MEGA 5 [40]. Maximum-likelihood (ML) trees were generated using FasTree [41]. For NJ and ML, the Jones-Taylor-Thornton (JTT) model was used with Gamma parameter 1.4. For MP, the tree-bisection-reconnection algorithm was used with all sites parameter and 100 maximum number of retained trees. In NJ and MP phylogenetic analysis, 1000 bootstrap replicates were obtained. For ML, the JTT model was used with 20 gamma categories (Gamma20) and the posterior probabilities support values for each node was computed by resampling 1,000 times the site likelihoods and performing the Shimodaira Hasegawa test [42]. The phylogenies for Rabring7/BCA2/BTLs, obtained with NJ, ML, and MP, were assessed to compare their agree with conventional taxonomic classification in Order, Family and Genera; got from the National Center of Biotechnology Information (NCBI) taxonomy server http://www.ncbi.nlm.nih.gov/Taxonomy. The tree phylogeny was displayed and edited by iTOL (Interactive Tree Of Life) at http://itol.embl.de/ [43]. We opted for a color code as described in Figure 2.

#### Generation of sequence LOGOs

To search for conserved motifs in Rabring7/BCA2 and BTL proteins, Multiple EM for Motif Elicitation (MEME) version 4.8.1 (http://meme.sdsc.edu/meme/cgi-bin/meme.cgi) was used as previously described. To simultaneously visualize the phylogeny and the predicted MEME conserved motifs, we represented each region with one shape symbol as follows: I, left pointing triangle; II, left and right pointing pentagrams; III, horizontal rectangle; IV, horizontal hexagon; and V, right pointing triangle. The sequence LOGOs generated were mapped to the five Rabring7/BCA2/BTL regions, a different color indicated more than one conserved sequence LOGO in a region. The Rabring7/BCA27/BTL proteins that displayed non-canonical distances between the residues defining the RING-H2 domain were not included in this analysis (see above). In this case, domain architecture was adjusted by sequence comparison with similar proteins.

#### Classification of BTLs from angiosperms

Phylogenetic analysis and BTL-generated pHMM LOGOs were used in order to classify BTLs. Six groups (A to F) were formed on a collapsed branch tree with local support less than 80% on ML method (Figure 5 and Table S3). ML, NJ, and MP methods resulted in a similar topology. The local supports were placed, ML/NJ/MP for values above 0.5 (ML) or 50% (NJ and MP). BTL sequences that were not included in this classification are: aly|936073, cpp|evm. TU.supercontig 1036.1, cpp|evm. TU.supercontig 34.207, cre|Cre10.g422050, egr|Eucgr.A02079, mgu|mgv1a010758m.g, olu|OSTLU 31341, ota|Ot04g04330, ppp|Pp1s161 109V6, ppp|Pp1s223 128V6, ppp|Pp1s37 80V6, set|Si004415m.g, smo|18755, smo|8109, and vcn|VOLCADRAFT 100265.

### Yeast two-hybrid screenings and assays

Fragments encompassing distinct regions of *ath*|*BTL4*|*At5g56340* were amplified by PCR from *A. thaliana* genomic DNA and cloned into the pGBKT7 plasmid (Clontech, Mountain View, CA) (segments are diagrammatically represented in Figure 9). The clones were verified by sequencing. Clone ath|BTL4 (III-IV–V) was used as bait in the yeast two-hybrid screenings, which was transformed into the *Saccharomyces cerevisiae* strain AH109, and a *A. thaliana* Matchmaker cDNA library constructed in plasmid pGAD10 (Clontech, Mountain View, CA) was screened in the assay. Transformants were selected on SC lacking Trp and Leu, and then screened under high-stringency selective conditions (medium lacking Trp, Leu, His, and Ade). To map regions in ath|BTL4 responsible for mediating the interaction with the positive clones, the twelve clones selected from the screen as positive were cotransformed into AH109 with clones containing regions III and V, together with empty vectors as controls. Only the combinations of the twelve clones with the clone containing region III of ath|BTL4 grew under high-stringency selective conditions.

To test whether the BZF region present in BTLs binds ubiquitin, fragments encompassing the BZF region ath|BTL4 were amplified by PCR from *A. thaliana* genomic DNA and then cloned into the pGBKT7 plasmid. These clones were verified by sequencing and used in the yeast two-hybrid assays (Figure 6). The clones containing the ubiquitin genes AtUBQ3, AtUBQ10, AtUBQ11, and AtUBQ14 in pGADT7 were obtained from a previous yeast-two hybrid screen (Medina and Guzman, unpublished results). The yeast two-hybrid screenings and assay were performed basically according to the manufacturer’s instructions as previously described (MATCHMAKER GAL4 Two-Hybrid System 3, Clontech, Mountain View, CA). In all cases, activation of His3 and Ade2 reporter genes was assessed in media supplemented with 7 mM 3-Amino-1,2,4-triazole (3-AT) to eliminate auto-activation background of the BZF clone.

## Supporting Information

Figure S1 **Phylogenetic trees of BTLs and Rabring7/BCA2s based on concatenated RING-H2 and BZF domains.** The topology were generated by the ML method; statistical significance in percentages above 50% for NJ, and MP, and posterior probability above 0.5 for ML methods is indicated on the nodes (ML/NJ/MP).
(PDF)

Figure S2 **Sequence LOGOs from previously identified motifs**.
(PDF)

Figure S3 Protein sequence alignment of the six BTL groups. The sequence alignments were performed using ClustalX 2.0.12; default colors were used. Regions encompassing sequence LOGOs are enclosed by rectangles.
(PDF)

Figure S4 **Domain architecture of Rabring7/BCA2s based on sequence LOGOs depicted next to the phylogenetic tree.**
(PDF)

Figure S5 **Domain architecture of BTLs based on sequence LOGOs depicted next to the phylogenetic tree.**
(PDF)

Table S1 Names and abbreviations of species used in this work. The color code of the species is the same as in Figure 2.
(PDF)

Table S2 **List of retrieved genes from animals, fungibold>, protists and plants**.
(PDF)

Table S3 **Catalog of 73 sequence LOGOs generated from 502 Rabring7/BCA2/BTLs.**
(PDF)

Table S4 Distribution of BTLs retrieved from 27 angiosperm species in 6 groups. Pairs of tandemly arrayed *BTLs* are shadowed in gray. *A. thaliana* BTLs are highlighted in yellow.
(PDF)

Table S5 **The ath|BTL4 interactors mapped to region III.**
(PDF)
