# Supplementary figures and images for: Expansion and Diversification of BTL Ring-H2 Ubiquitin Ligases in Angiosperms: Putative Rabring7/BCA2 Orthologs
Source: PLoS One. 2013 Aug 8;8(8):e72729. doi: 10.1371/journal.pone.0072729 (PMC3738576; doi:10.1371/journal.pone.0072729)

## BTLs Phylogeny.

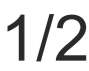

Figure S1. (continued).

Rabring7/BCA2s Phylogeny.

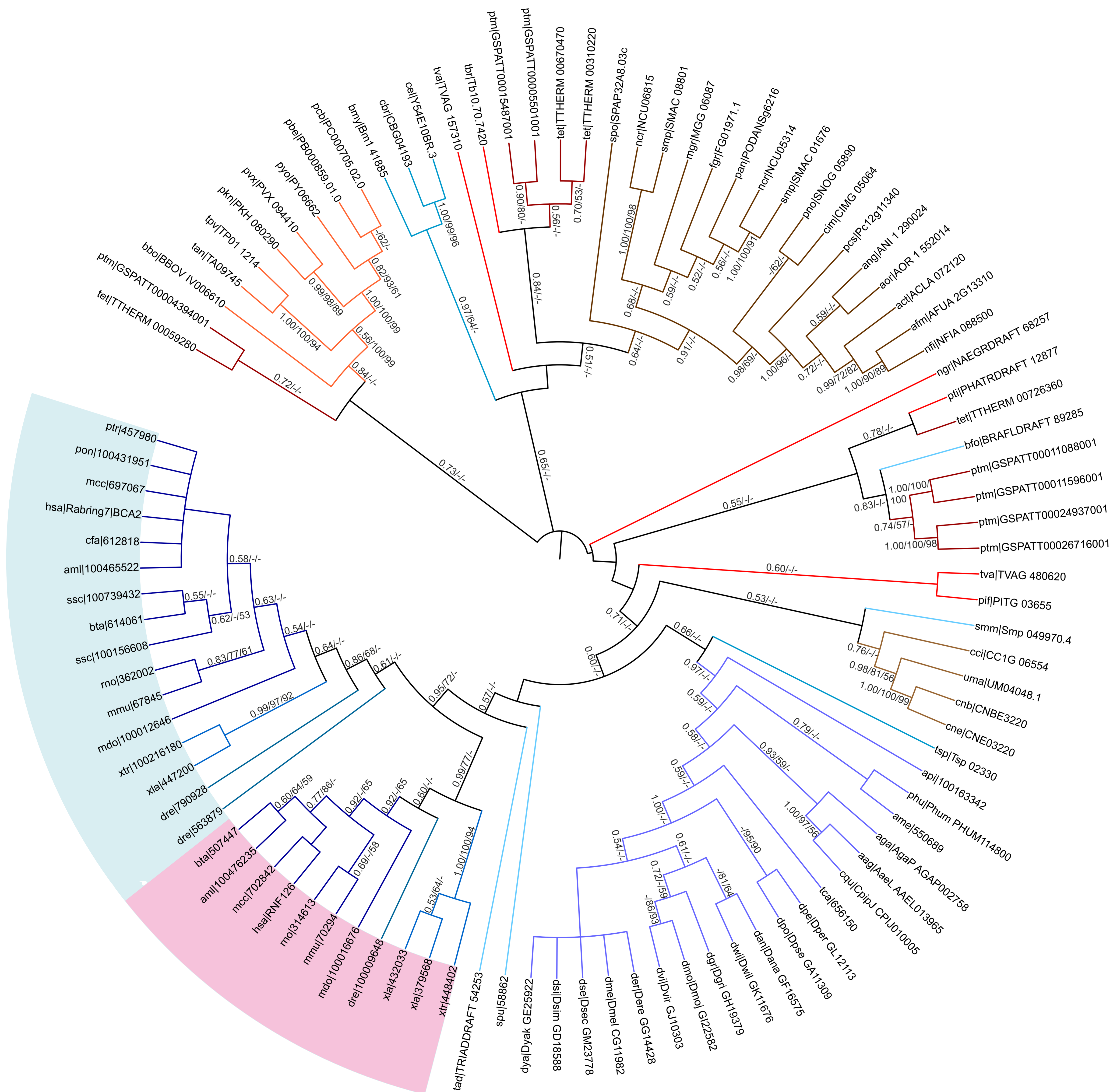

Supplement: Figure S1 — Phylogenetic trees of BTLs and Rabring7/BCA2s based on concatenated RING-H2 and BZF domains. The topology were generated by the ML method; statistical significance in percentages above 50% for NJ, and MP, and posterior probability above 0.5 for ML methods is indicated on the nodes (ML/NJ/MP). (PDF) [file pone.0072729.s001.pdf]

Figure S2. Sequence LOGOs from previously identified motifs.

(A) GLD-like motif

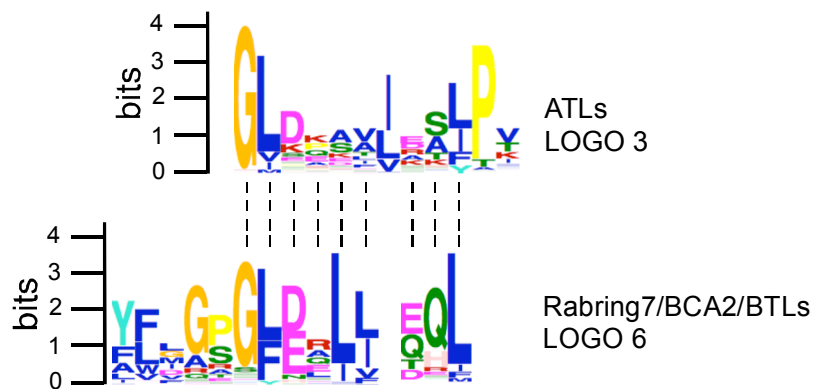

(B) AKT domain

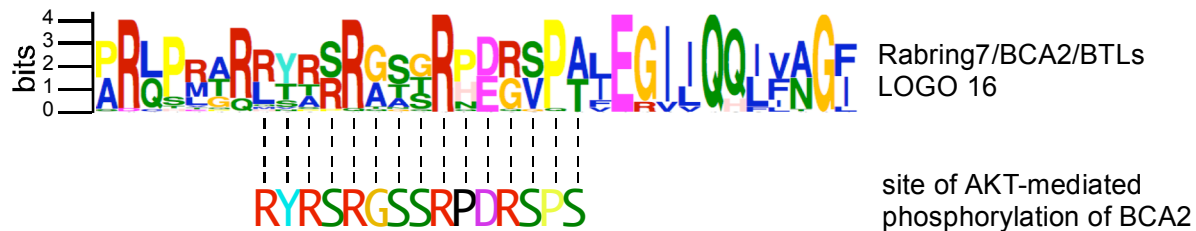

(C) DUF1117

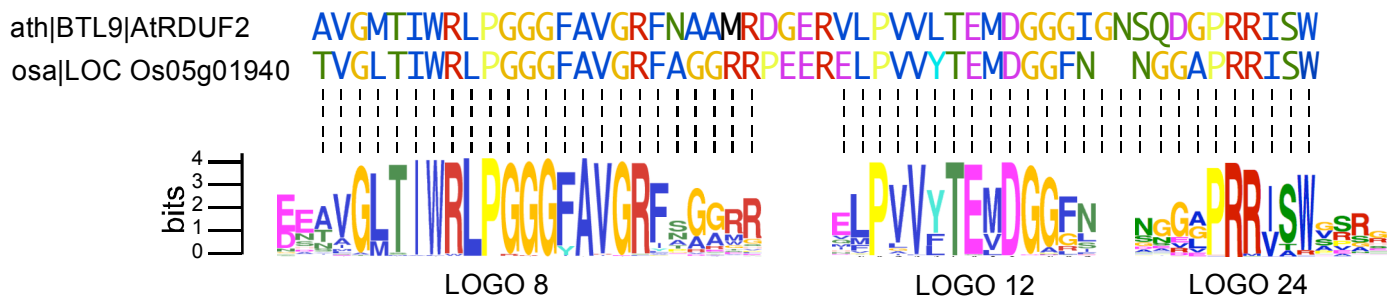

Supplement: Figure S2 — Sequence LOGOs from previously identified motifs. (PDF) [file pone.0072729.s002.pdf]
