# Supplementary material for: Expansion and Diversification of BTL Ring-H2 Ubiquitin Ligases in Angiosperms: Putative Rabring7/BCA2 Orthologs
Source: PLoS One. 2013 Aug 8;8(8):e72729. doi: 10.1371/journal.pone.0072729 (PMC3738576; doi:10.1371/journal.pone.0072729)

FIGURE S3. Sequence alignment of six BTL groups.

Group A

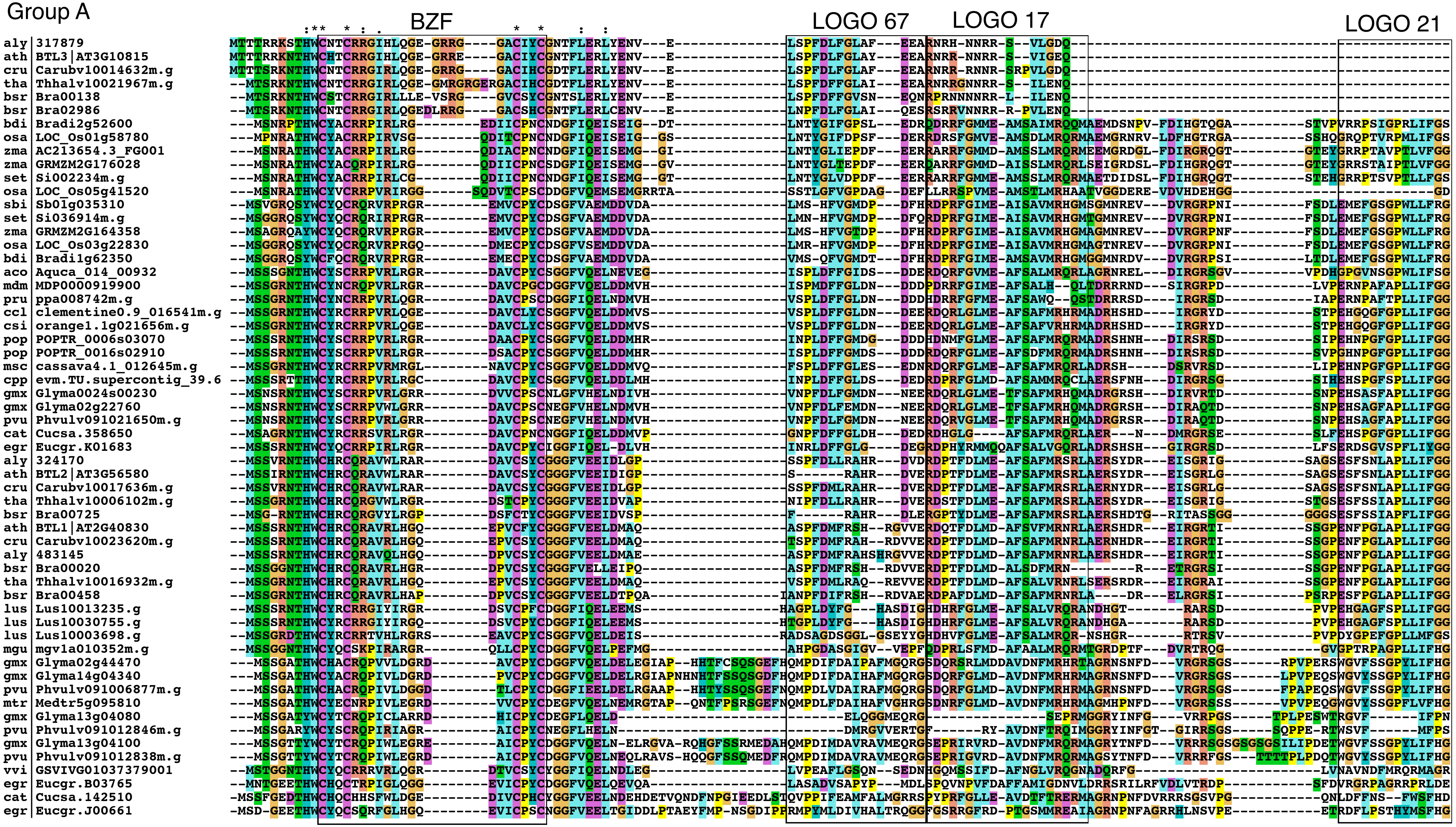

Group A (continued)

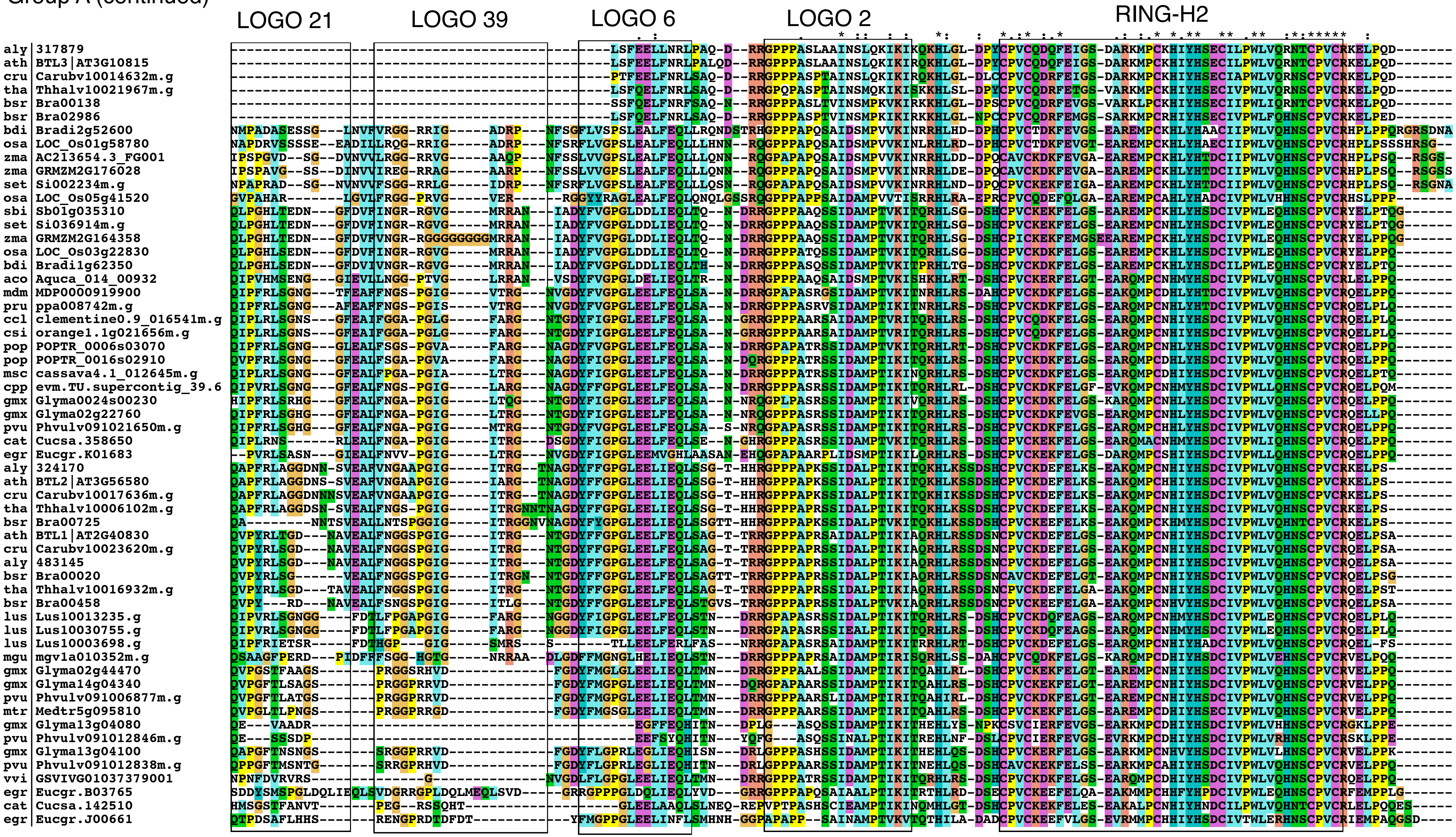

Group A (continued)

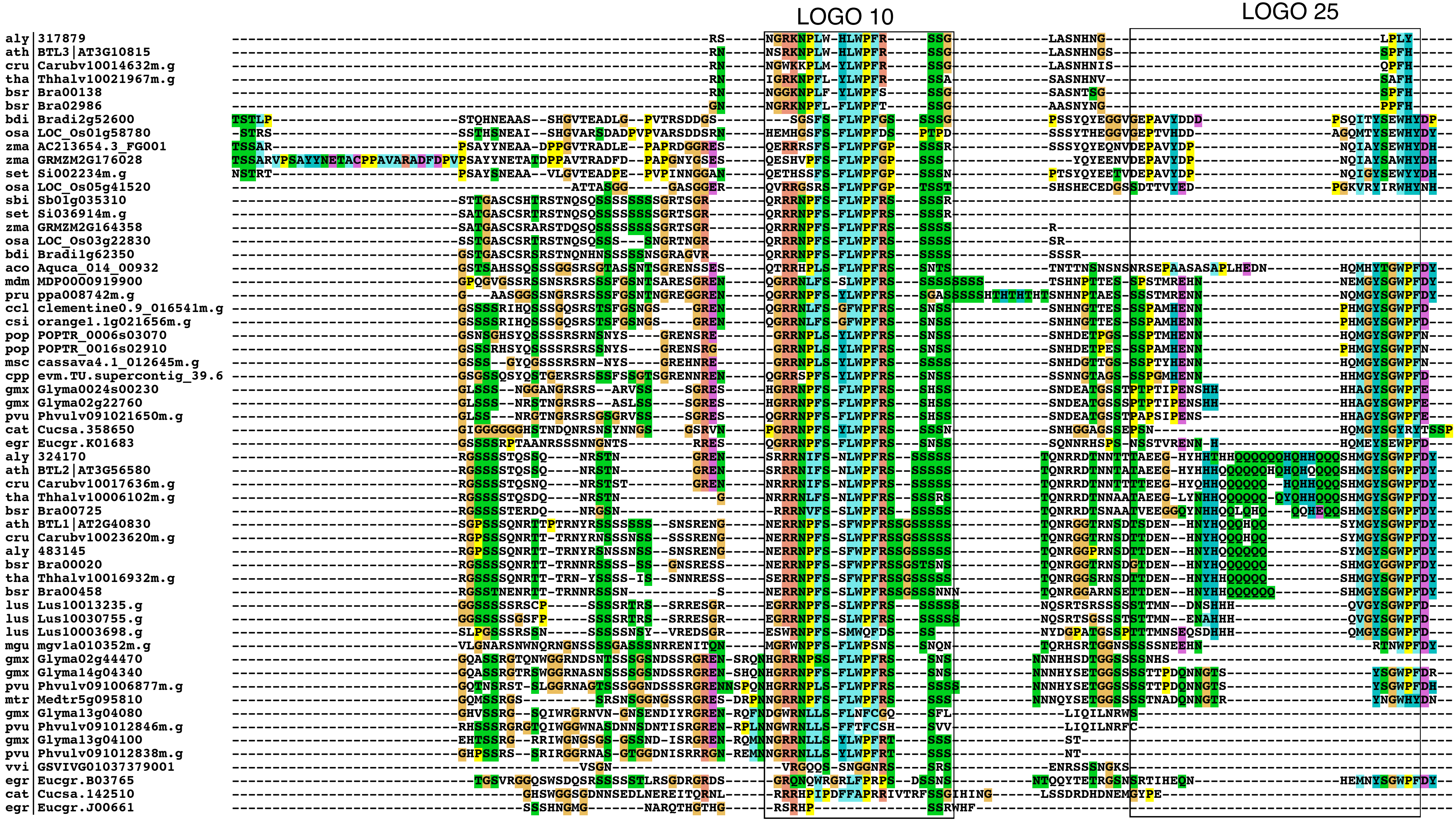

Group B

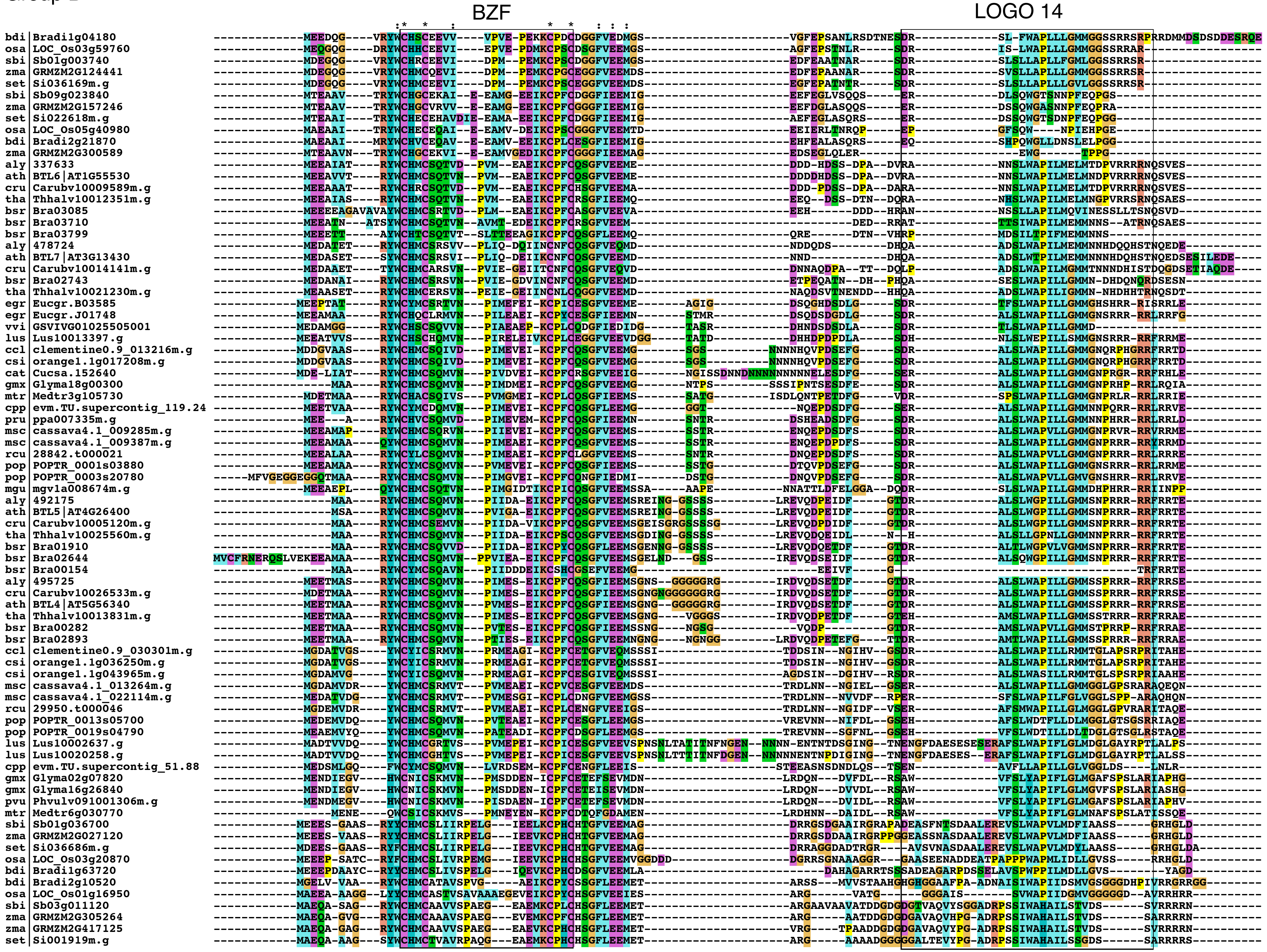
BZF
LOGO 14
bdi Bradi1g04180
osa LOC_Os03g59760
sbi Sb01g003740
zma GRMZM2G124441
set Si036169m.g
sbi Sb09g023840
zma GRMZM2G157246
set Si022618m.g
osa LOC_Os05g40980
bdi Bradi2g21870
zma GRMZM2G300589
aly 337633
ath BTL6|AT1G55530
cru Carubv10009589m.g
tha Thhalv10012351m.g
bsr Bra03085
bsr Bra03710
bsr Bra03799
aly 478724
ath BTL7|AT3G13430
cru Carubv10014141m.g
bsr Bra02743
tha Thhalv10021230m.g
egr Eucgr.B03585
egr Eucgr.J01748
vvi GSVIVG01025505001
lus Lus10013397.g
ccl clementine0.9_013216m.g
csi orange1.1g017208m.g
cat Cucsa.152640
gmx Glyma18g00300
mtr Medtr3g105730
cpp evm.TU.supercontig_119.24
pru ppa007335m.g
msc cassava4.1_009285m.g
msc cassava4.1_009387m.g
rcu 28842.t000021
pop POPTR_0001s03880
pop POPTR_0003s20780
mgu mgv1a008674m.g
aly 492175
ath BTL5|AT4G26400
cru Carubv10005120m.g
tha Thhalv10025560m.g
bsr Bra01910
bsr Bra02644
bsr Bra00154
aly 495725
cru Carubv10026533m.g
ath BTL4|AT5G56340
tha Thhalv10013831m.g
bsr Bra00282
bsr Bra02893
ccl clementine0.9_030301m.g
csi orange1.1g036250m.g
csi orange1.1g043965m.g
msc cassava4.1_013264m.g
msc cassava4.1_022114m.g
rcu 29950.t000046
pop POPTR_0013s05700
pop POPTR_0019s04790
lus Lus10002637.g
lus Lus10020258.g
cpp evm.TU.supercontig_51.88
gmx Glyma02g07820
gmx Glyma16g26840
pvu Phvulv091001306m.g
mtr Medtr6g030770
sbi Sb01g036700
zma GRMZM2G027120
set Si036686m.g
osa LOC_Os03g20870
bdi Bradi1g63720
bdi Bradi2g10520
osa LOC_Os01g16950
sbi Sb03g011120
zma GRMZM2G305264
zma GRMZM2G417125
set Si001919m.g

Group B (continued)

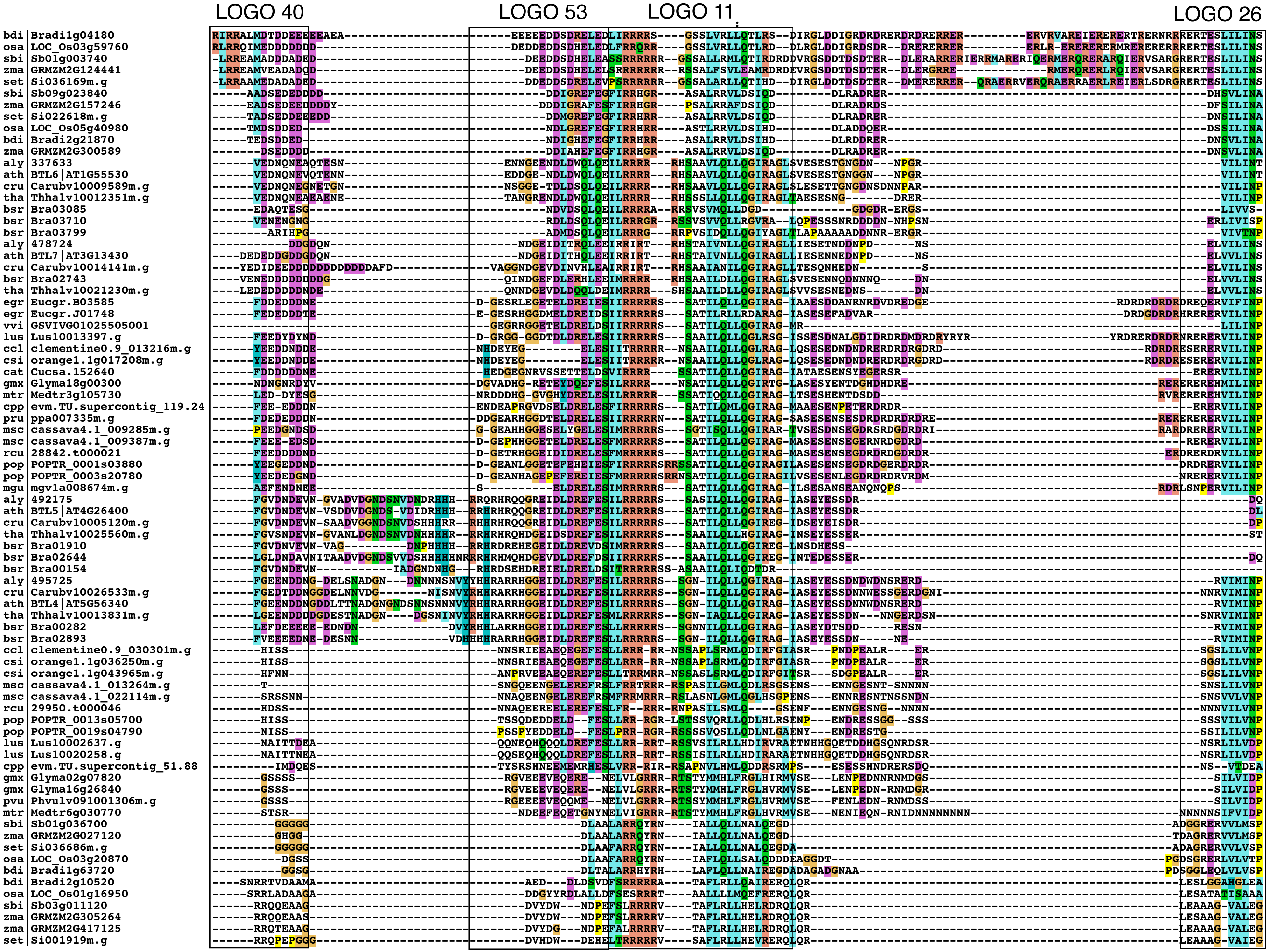

Group B (continued)

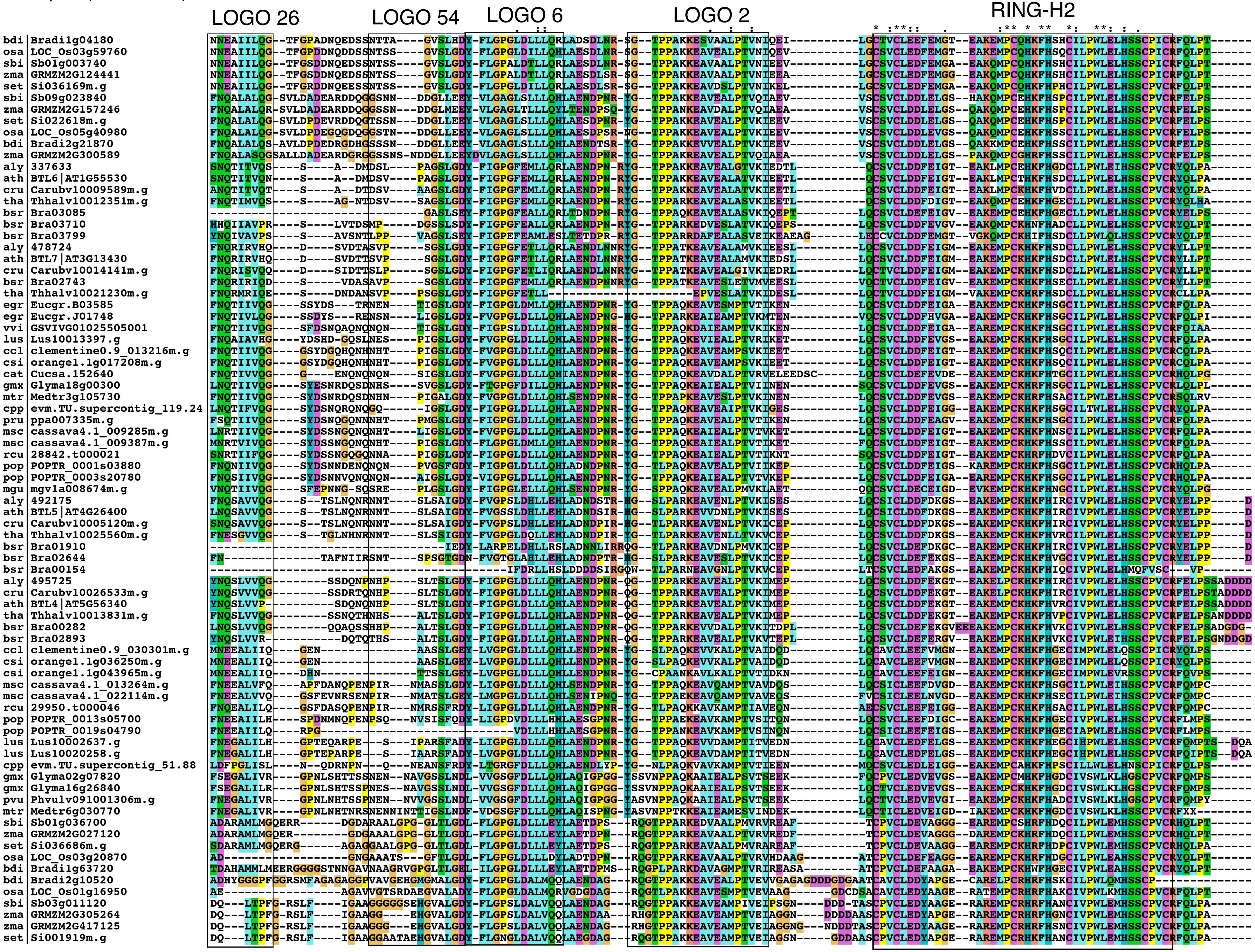

Group B (continued)

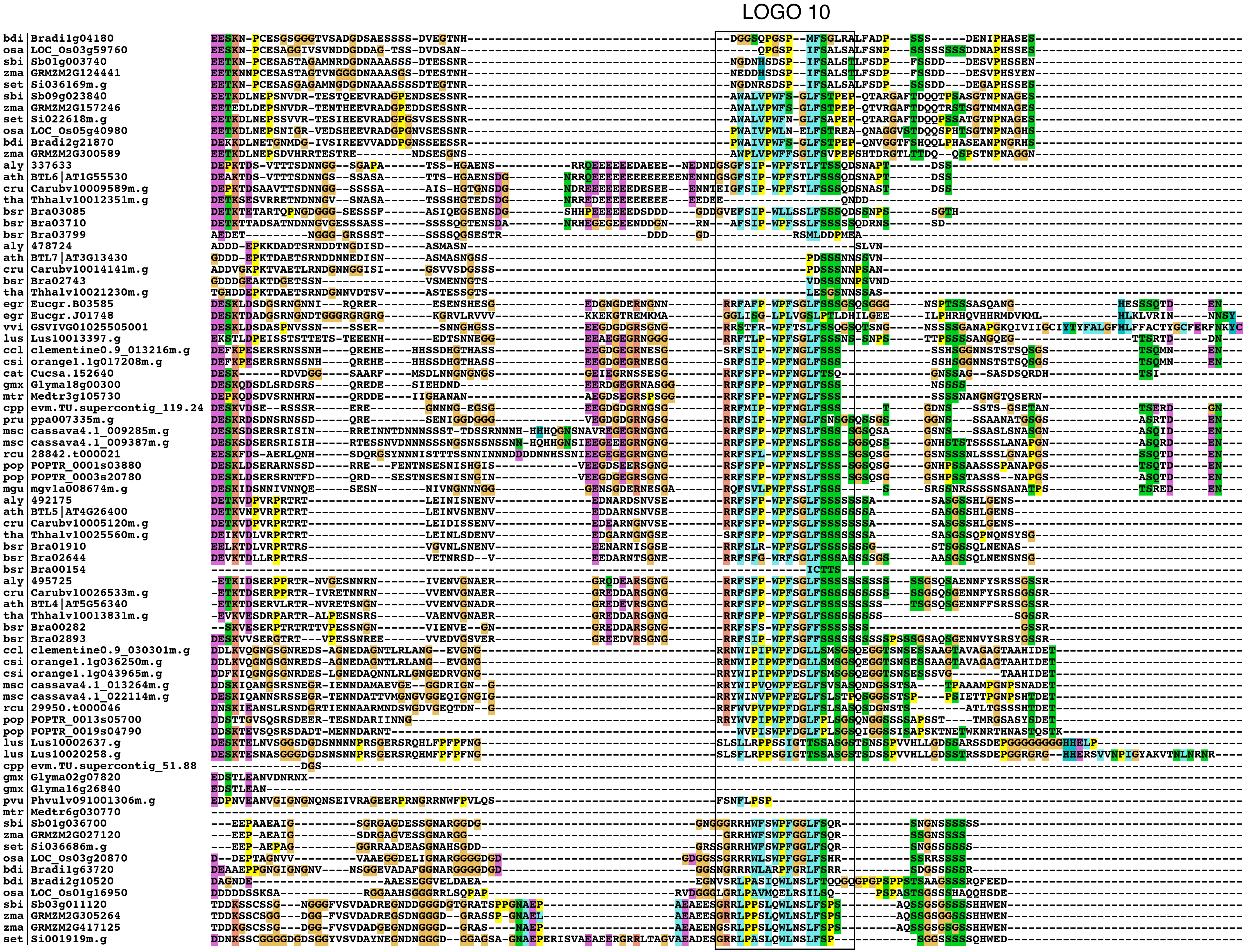

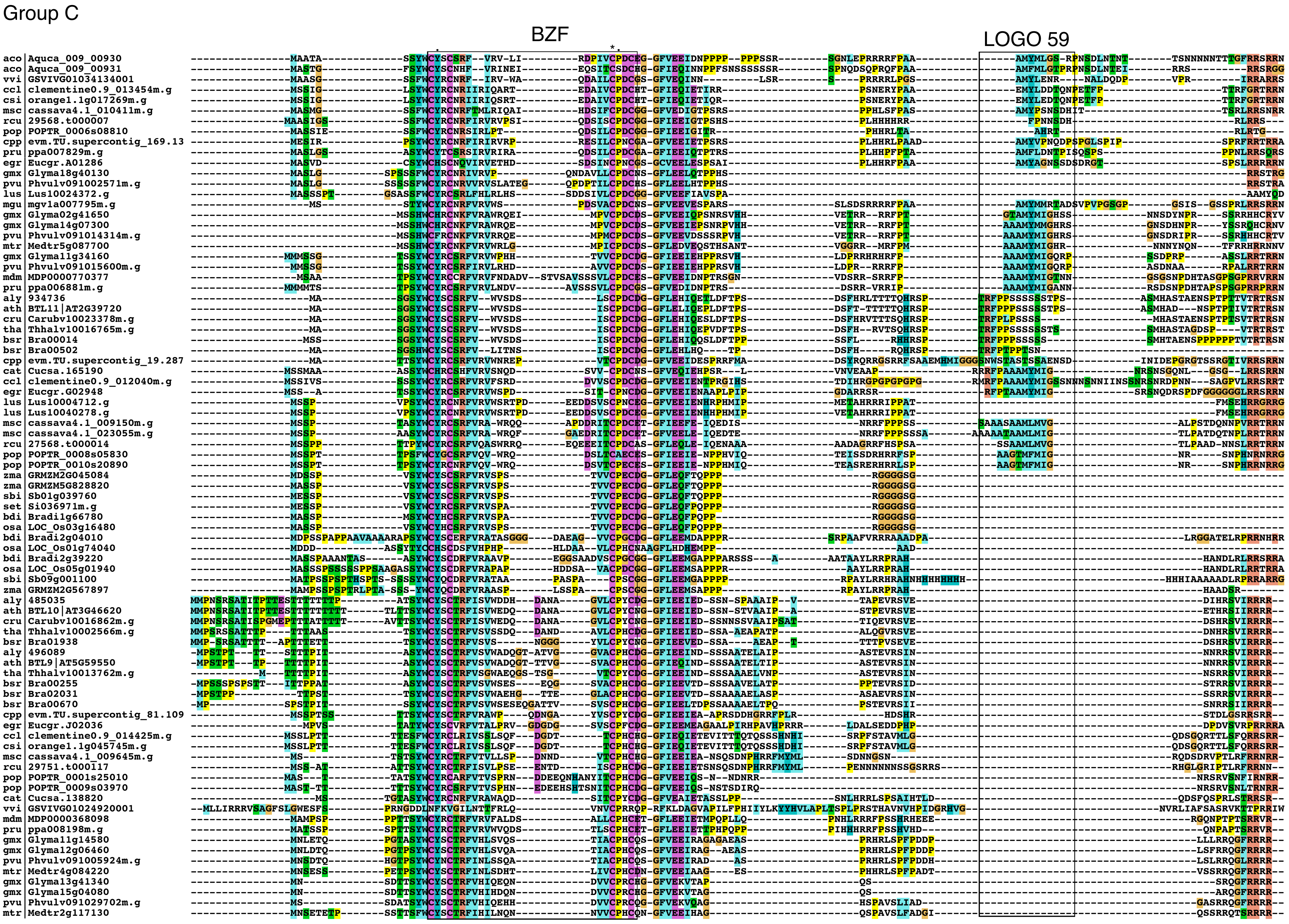
Group C
BZF
LOGO 59

Group C (continued)

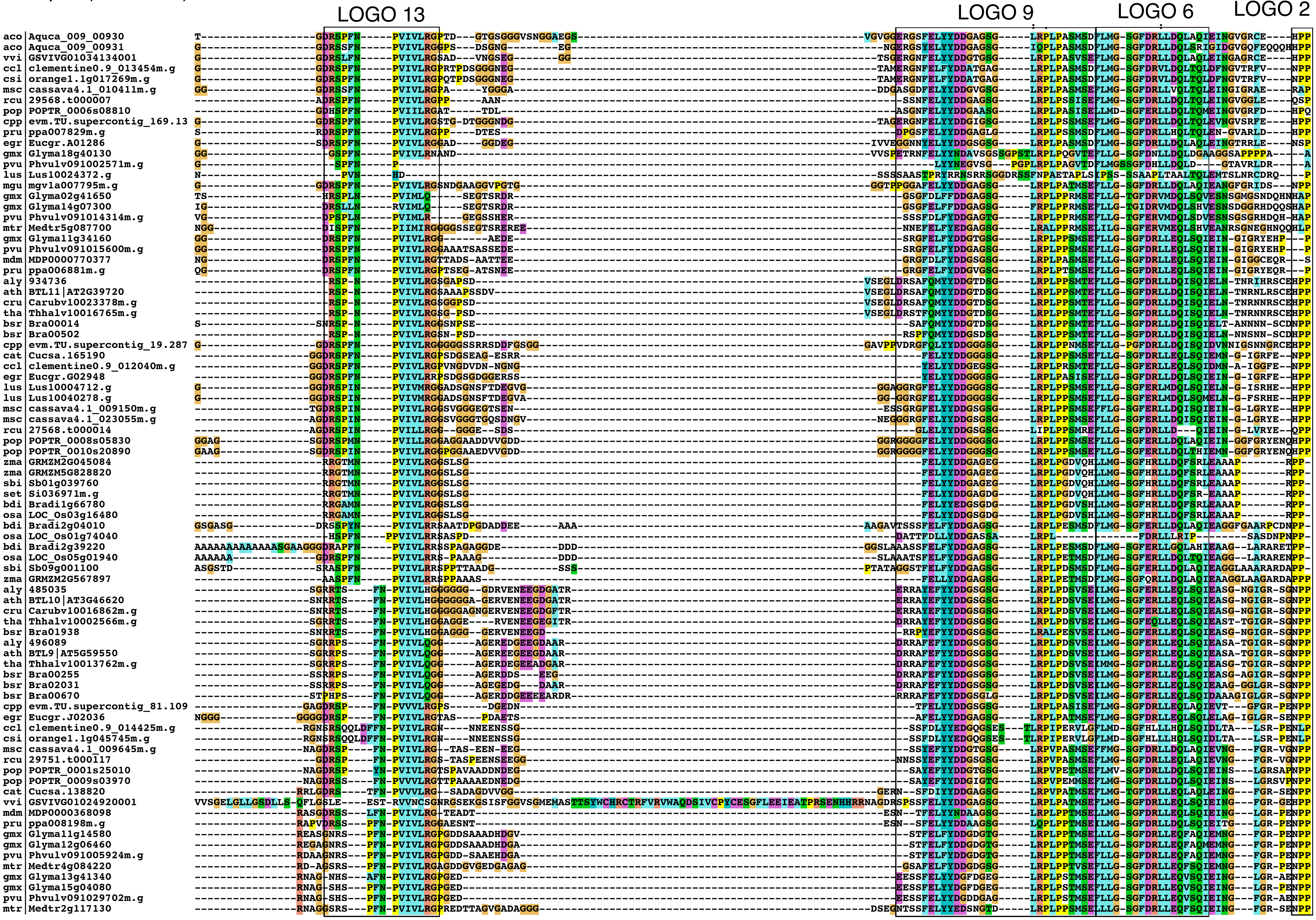

Group C (continued)

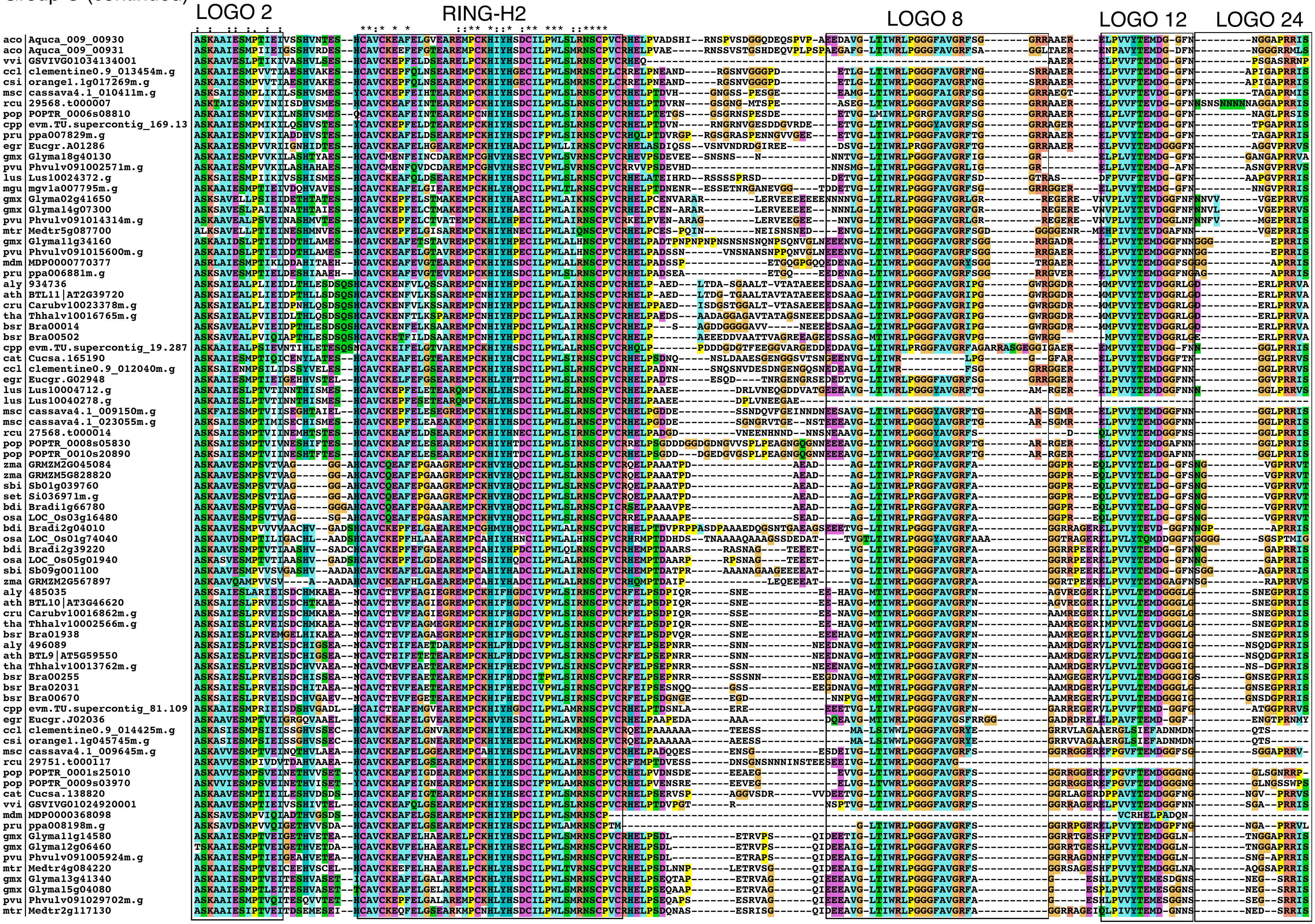

Group C (continued)

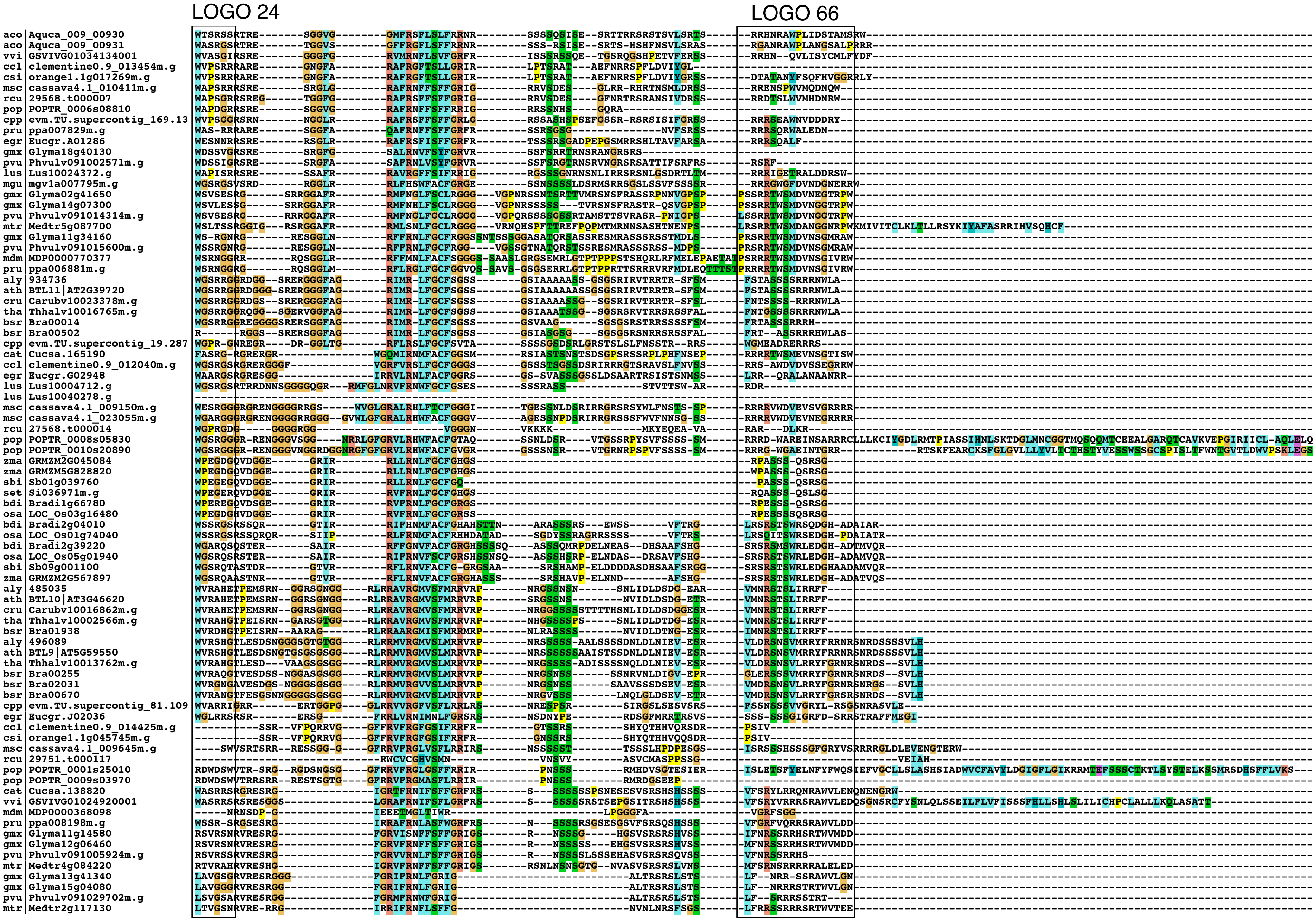

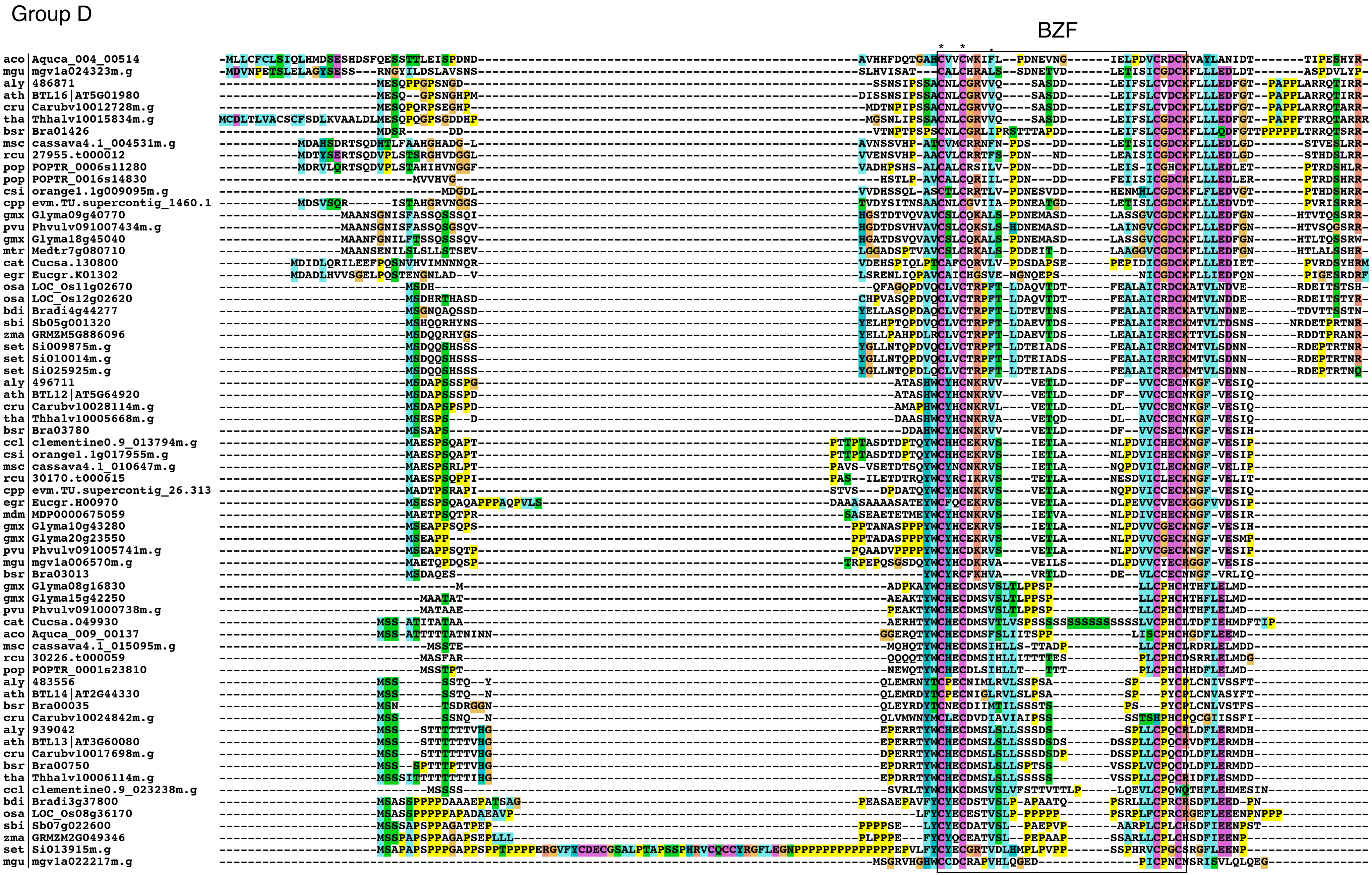
Group D
BZF
aco Aquca_004_00514
mgu mgv1a024323m.g
aly 486871
ath BTL16|AT5G01980
cru Carubv10012728m.g
tha Thhalv10015834m.g
bsr Bra01426
msc cassava4.1_004531m.g
rcu 27955.t000012
pop POPTR_0006s11280
pop POPTR_0016s14830
csi orange1.1g009095m.g
cpp evm.TU.supercontig_1460.1
gmx Glyma09g40770
pvu Phvulv091007434m.g
gmx Glyma18g45040
mtr Medtr7g080710
cat Cucsa.130800
egr Eucgr.K01302
osa LOC_Os11g02670
osa LOC_Os12g02620
bdi Bradi4g44277
sbi Sb05g001320
zma GRMZM5G886096
set Si009875m.g
set Si010014m.g
set Si025925m.g
aly 496711
ath BTL12|AT5G64920
cru Carubv10028114m.g
tha Thhalv10005668m.g
bsr Bra03780
ccl clementine0.9_013794m.g
csi orange1.1g017955m.g
msc cassava4.1_010647m.g
rcu 30170.t000615
cpp evm.TU.supercontig_26.313
egr Eucgr.H00970
mdm MDP0000675059
gmx Glyma10g43280
gmx Glyma20g23550
pvu Phvulv091005741m.g
mgu mgv1a006570m.g
bsr Bra03013
gmx Glyma08g16830
gmx Glyma15g42250
pvu Phvulv091000738m.g
cat Cucsa.049930
aco Aquca_009_00137
msc cassava4.1_015095m.g
rcu 30226.t000059
pop POPTR_0001s23810
aly 483556
ath BTL14|AT2G44330
bsr Bra00035
cru Carubv10024842m.g
aly 939042
ath BTL13|AT3G60080
cru Carubv10017698m.g
bsr Bra00750
tha Thhalv10006114m.g
ccl clementine0.9_023238m.g
bdi Bradi3g37800
osa LOC_Os08g36170
sbi Sb07g022600
zma GRMZM2G049346
set Si013915m.g
mgu mgv1a022217m.g

Group D (continued)

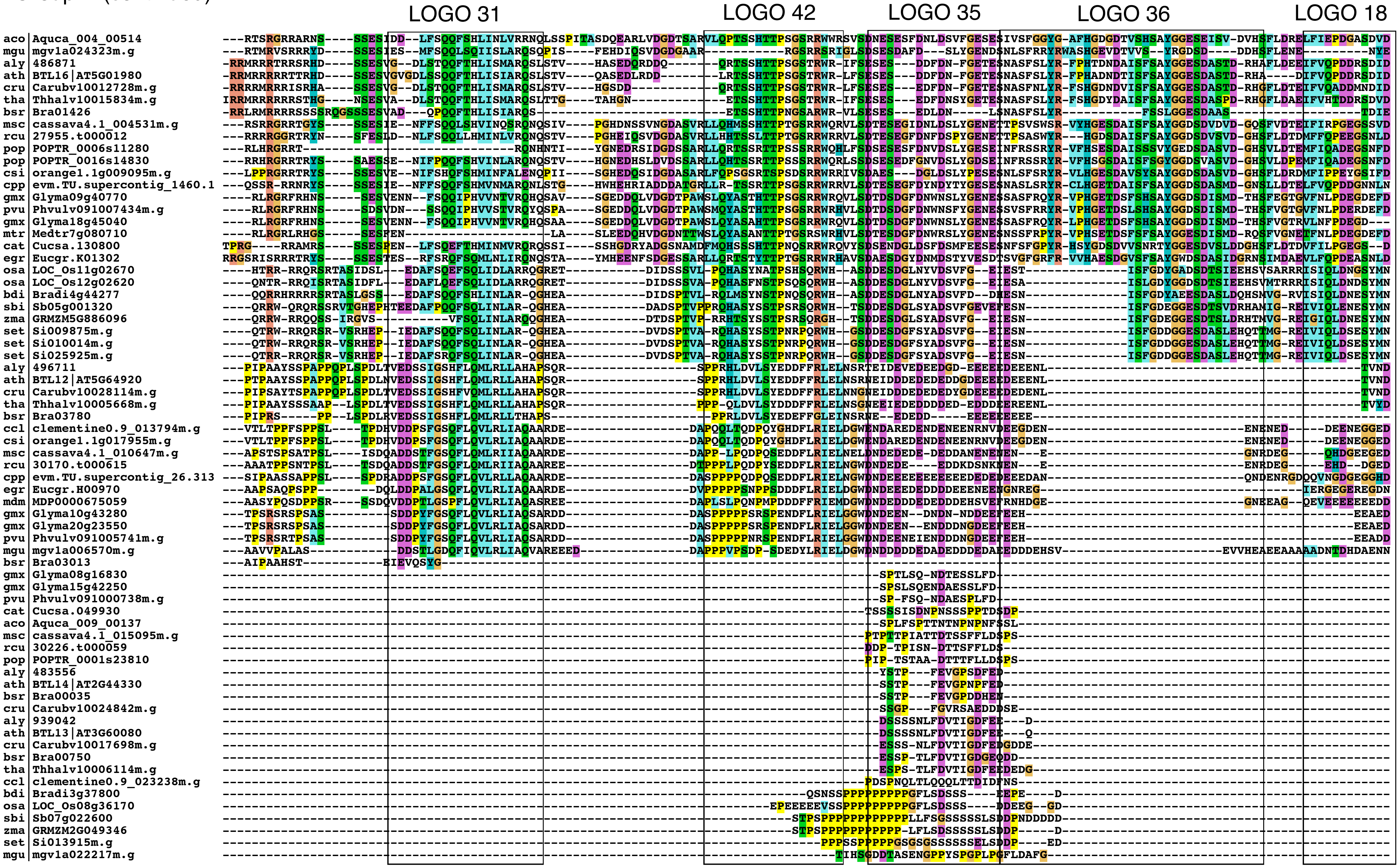

Group D (continued)

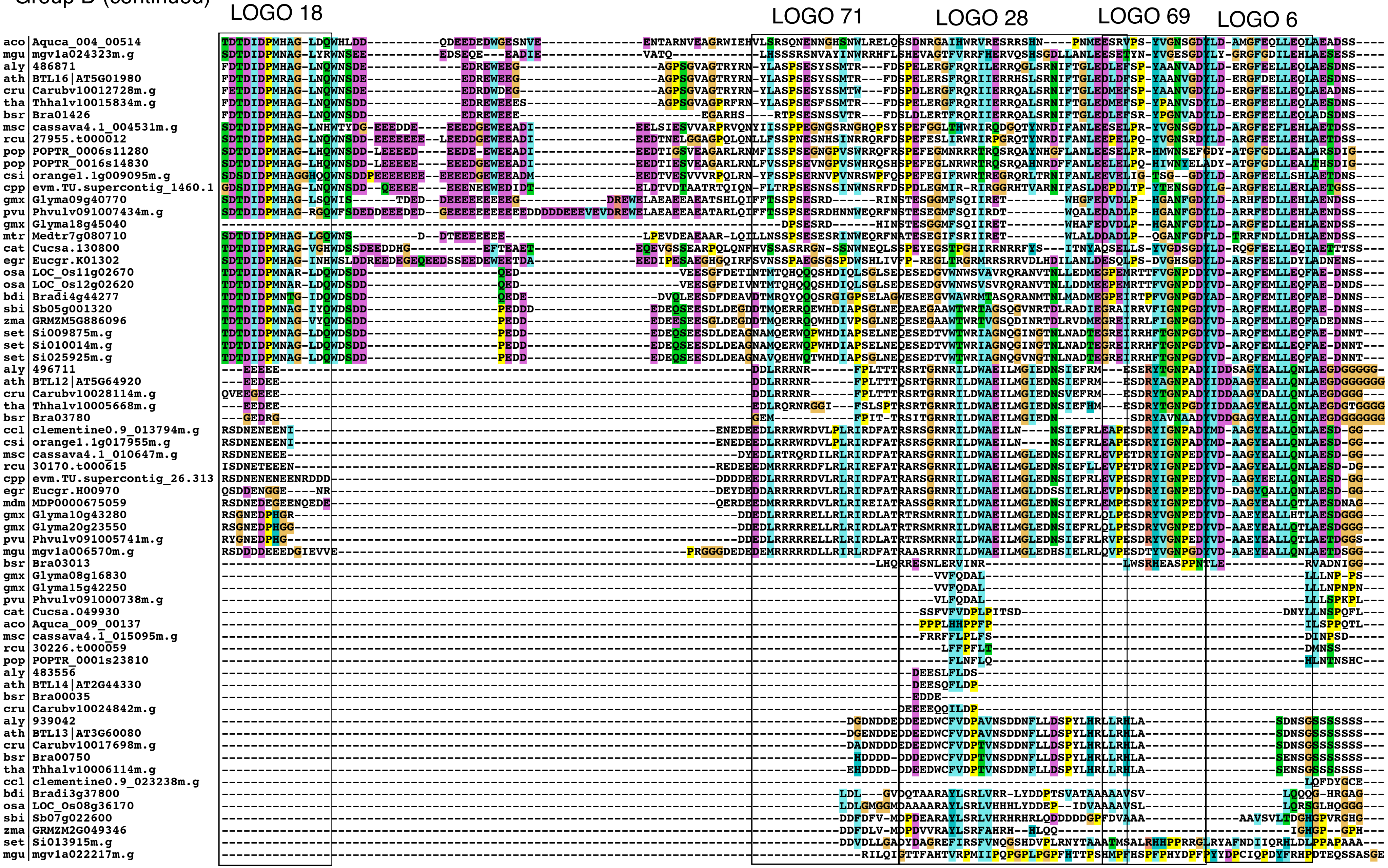

Group D (continued)

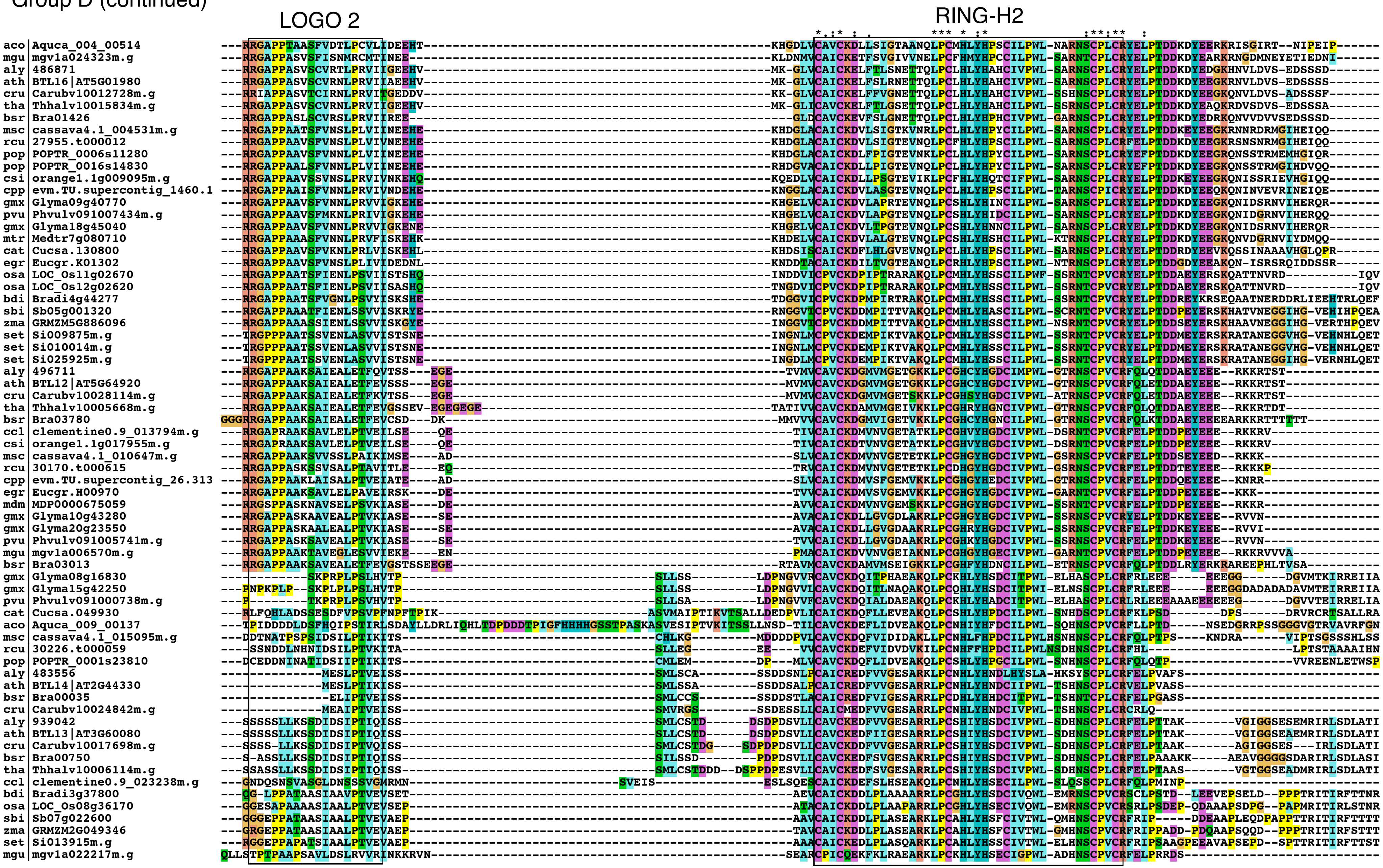

Group D (continued)

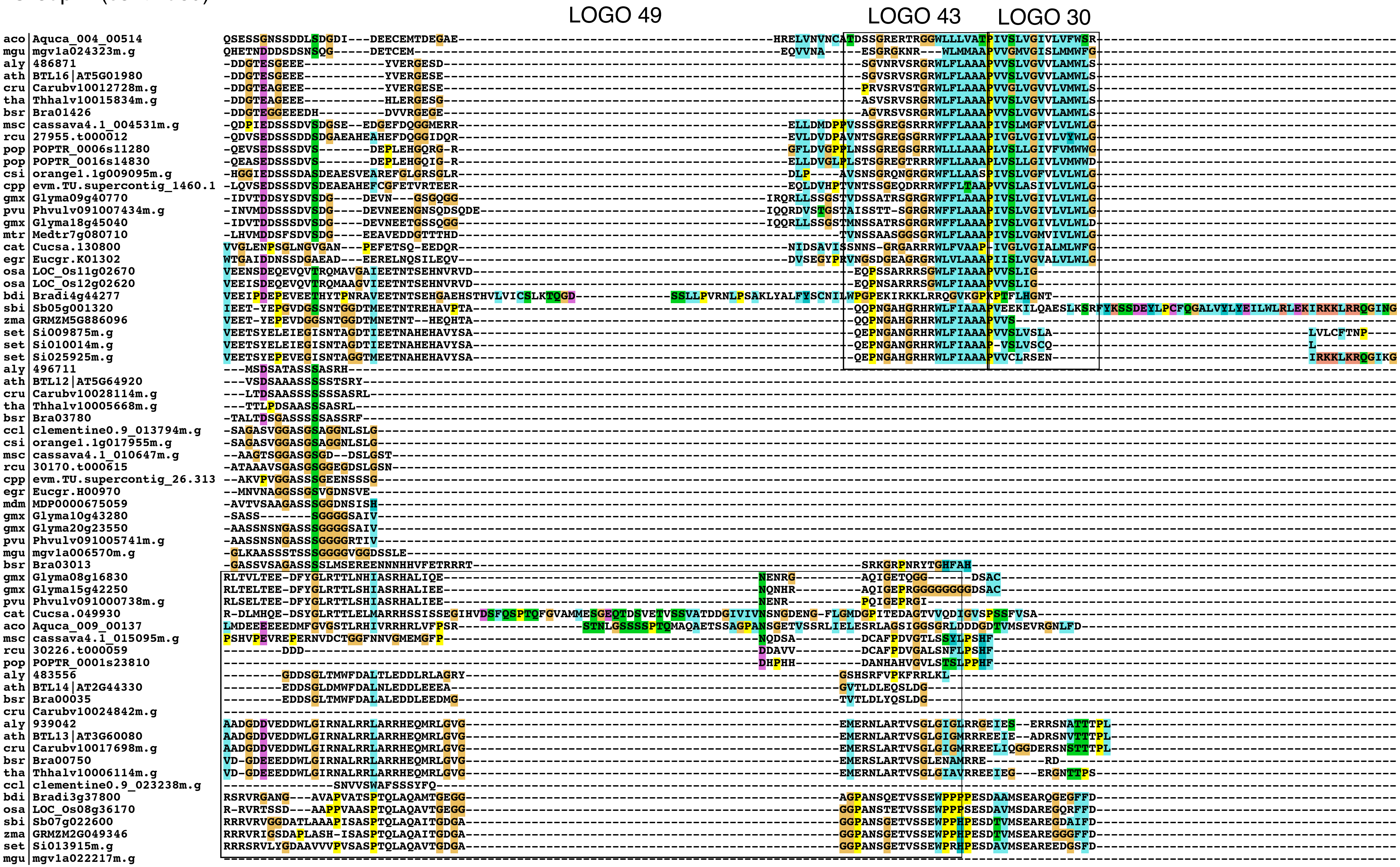

Group D (continued)

LOGO 58

```
aco|Aquca_004_00514            ---NPLVGRVGPRGQCGLYGQHQQQVQIPLRNPRDGQGTNRNRRWWSFF---
mgu|mgv1a024323m.g             ---NSSAAAAAYR---RSPTSAHIHRPKD---NGQRRWWSSFF---
aly|486871                     ---NQQRR---DIAISHS---QRGNRTRRWLPFF---
ath|BTL16|AT5G01980            ---SPHRR---GIAISHS---QRENRTRRWLPFF---
cru|Carubv10012728m.g          ---NPQRR---DIAVSHS---QRGSRTRRWLPFF---
tha|Thhalv10015834m.g          ---NPQRR---DIVISHN---QRGNRTRRWLPFF---
bsr|Bra01426                   ---NPQRR---NIVFSHS---QRGNRSRRWLPFF---
msc|cassava4.1_004531m.g       ---NNQGRGPTRHCNLPERGL---HQIHVPGSSTPNQRGNRSRRWWSLF---
rcu|27955.t000012              ---SPQRRGPTHHCDVPQRGL---HQIRAPGSSAPSQRGNRSRRWWSFF---
pop|POPTR_0006s11280           ---NPQGRRANGHCNFSGRGL---HQIQVSGSSQLNQRGNGRRRWWSFF---
pop|POPTR_0016s14830           ---NPQGRRANSHGNFPERGL---YQIQVSGPPQPNQKVNERRRWWFFS---
csi|orange1.1g009095m.g        ---NPP---TARWGPVTQP---QISFHNSASPSRTENRSRRWWSLF---
cpp|evm.TU.supercontig_1460.1  ---TPG---RNGLASPYNP---HPIHY---QRGNRRRRWWPFF---
gmx|Glyma09g40770              ---NNSQNEGSRHLGSHYLSG--QNQHAVHAYSSPNQRESRSRRWWCPF---
pvu|Phvulv091007434m.g         ---NNSEIEGNRHLGSHYLSG--QNQHAVHAYSAPNQRESRSRRWWCPF---
gmx|Glyma18g45040              ---IWAVTTYPGKISTPFMLIPLQTRGRVEAEGGGAHSNLCFKDLMSKYLIIY---
mtr|Medtr7g080710              ---SNSQIEETRHSSTRYLSV--QNQHPVHVSGSQNQRESRSRRWWCPF---
cat|Cucsa.130800               ---NPLCDQR--VSGGQLTDG---RQLDAHSAESSNQRENRSRRWWQIV---
egr|Eucgr.K01302               ---NPLAQRRRATNNHNFTEGH-LRQVPGFGAPASNRSDRTNRRWWFPF---
osa|LOC_Os11g02670             ---FALVLCFTNPAR---SGRRQLYCRSPSATEVHVGTKK---SWWSMF---
osa|LOC_Os12g02620             ---FALVLCFTNPAR---SGRRQLYCRSPSATEVHVDTKK---SWWSMF---
bdi|Bradi4g44277               ---REMKSICHELKPVKK--QDSNNYISTLFPHLLVWREAYGPVFQYSTGGRE---ILHVSQPDMVKDIGHWTPSELGKPSYLKKSRKALLGGGLFTVNGDEWAYQRKVMAPEFFMDKIKDMIQLIEDATAPLLEAWESILEGAGGSTE
sbi|Sb05g001320                PKPTLLYGNTQEMKRIRQELKCVQRKDRDMNGYISTIFPHFLLWRKTYDSCLRKDISKRFVFKWTENLKNGYSTLWSSVLLLCHYMVFENWDRVRVPLLNRSFGGILTANGDLWAYEKKILAPQFFVEKIKGMIGLIVDATVPLLQSWENML---
zma|GRMZM5G886096              ---LVSLALVLCFTNPAGN---VRRQLCRRSQ---TTTTAHVDTRRSWWSMF---
set|Si009875m.g                ---AGNVRRQLYHRSQSTTTTR--VDTRRSWWSMF---
set|Si010014m.g                ---
set|Si025925m.g                PEPTVLYGNTREMKRIKEGLKIVQT--QDANNYLSSVFPYLLLWRETYGPVFIYSTG---ALEILHVSDPEMVKDIGHRTPSELGKPNYLKRSRKALFGGGLFTLNGNDWAYQRKLIAPEFFMDKIKGMIELVEDATAPLLESWESLL---
aly|496711                     ---
ath|BTL12|AT5G64920            ---
cru|Carubv10028114m.g          ---
tha|Thhalv10005668m.g          ---
bsr|Bra03780                   ---
ccl|clementine0.9_013794m.g    ---
csi|orange1.1g017955m.g        ---
msc|cassava4.1_010647m.g       ---
rcu|30170.t000615              ---
cpp|evm.TU.supercontig_26.313  ---
egr|Eucgr.H00970               ---
mdm|MDP0000675059              ---
gmx|Glyma10g43280              ---
gmx|Glyma20g23550              ---
pvu|Phvulv091005741m.g         ---
mgu|mgv1a006570m.g             ---
bsr|Bra03013                   ---
gmx|Glyma08g16830              ---
gmx|Glyma15g42250              ---
pvu|Phvulv091000738m.g         ---
cat|Cucsa.049930               ---
aco|Aquca_009_00137            ---
msc|cassava4.1_015095m.g       ---
rcu|30226.t000059              ---
pop|POPTR_0001s23810           ---
aly|483556                     ---
ath|BTL14|AT2G44330            ---
bsr|Bra00035                   ---
cru|Carubv10024842m.g          ---
aly|939042                     ---
ath|BTL13|AT3G60080            ---
cru|Carubv10017698m.g          ---
bsr|Bra00750                   ---
tha|Thhalv10006114m.g          ---
ccl|clementine0.9_023238m.g    ---
bdi|Bradi3g37800               ---
osa|LOC_Os08g36170             ---
sbi|Sb07g022600                ---
zma|GRMZM2G049346              ---
set|Si013915m.g                ---
mgu|mgv1a022217m.g             ---
```

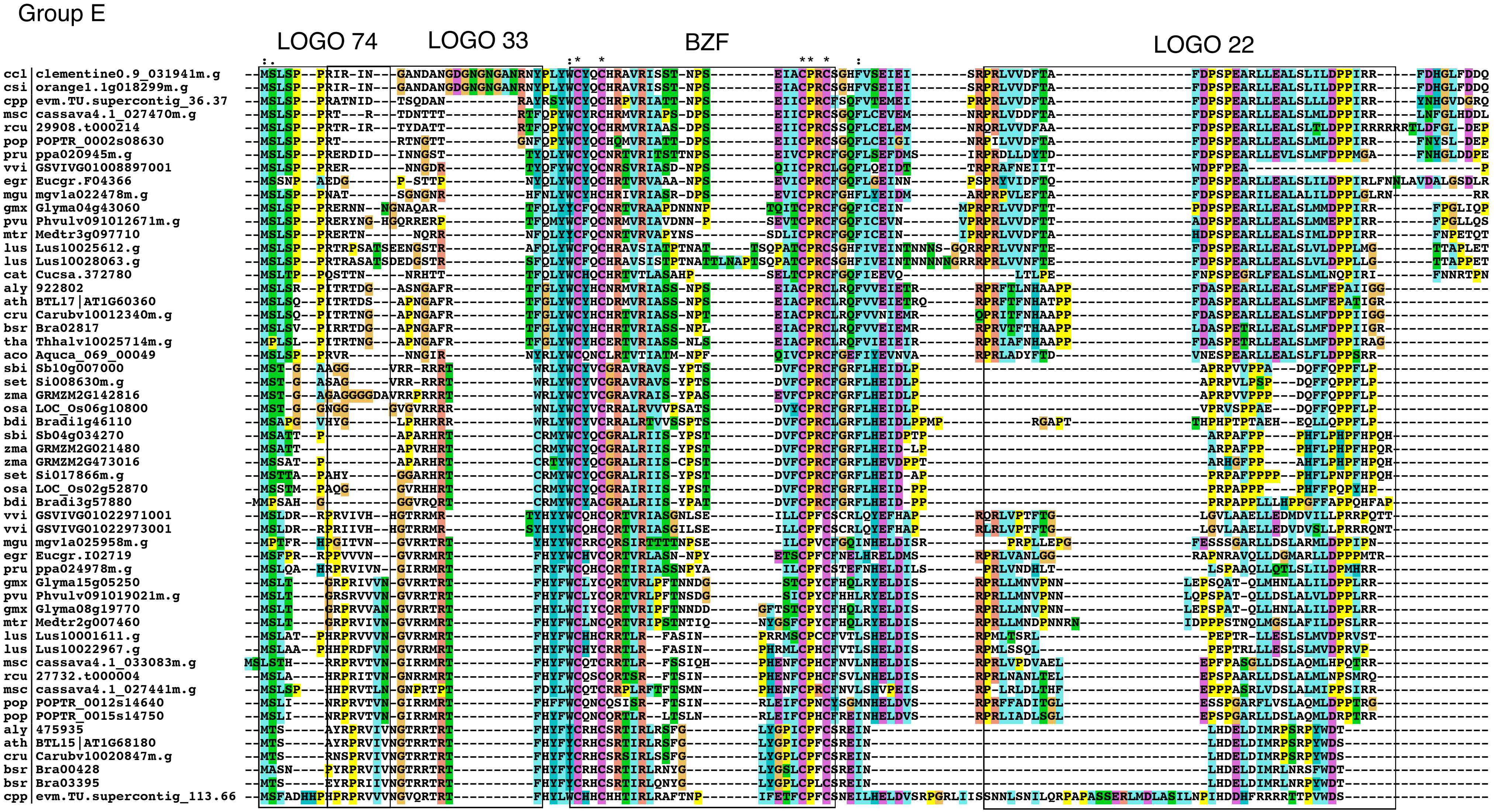
Group E
LOGO 74
LOGO 33
BZF
LOGO 22

Group E (continued)

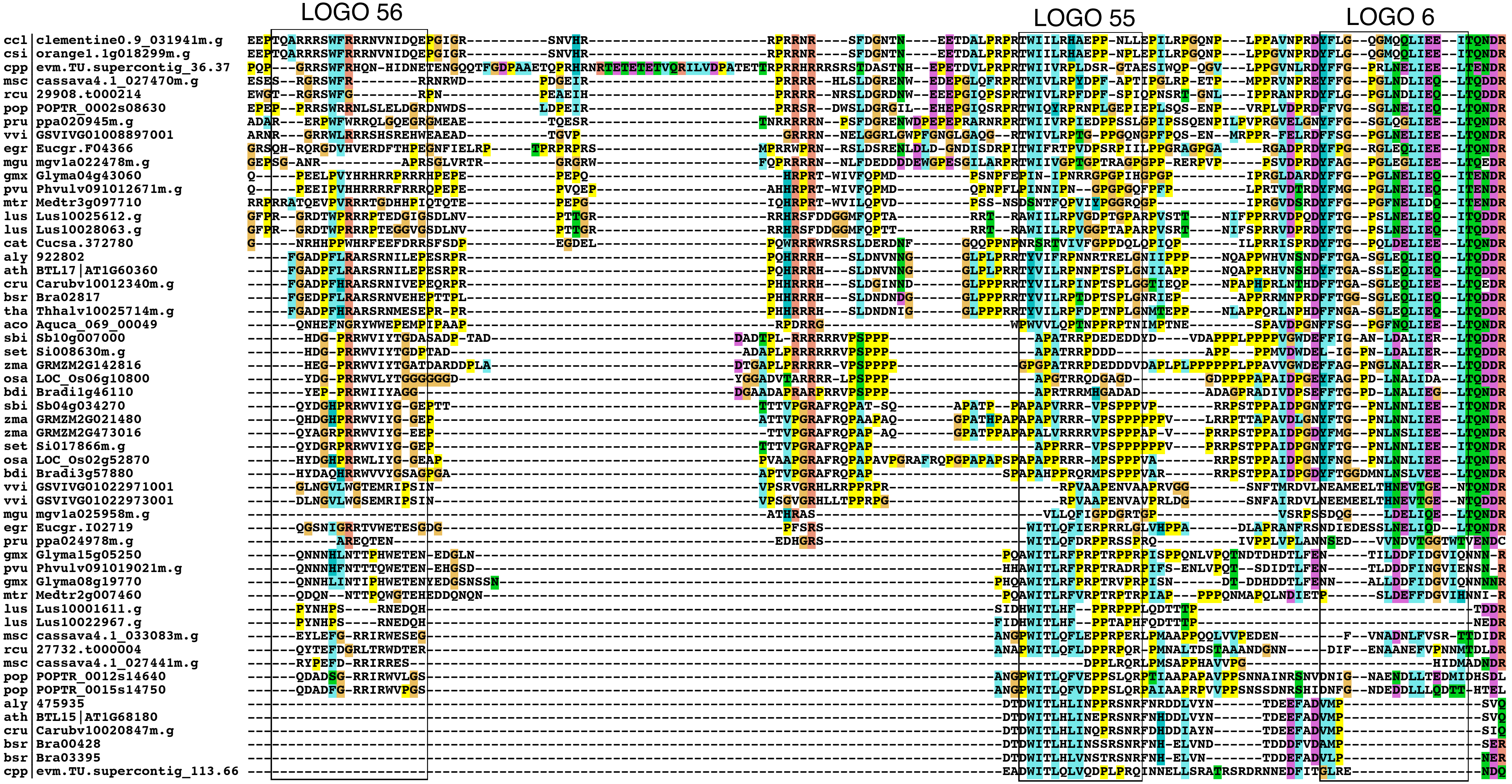

Group E (continued)

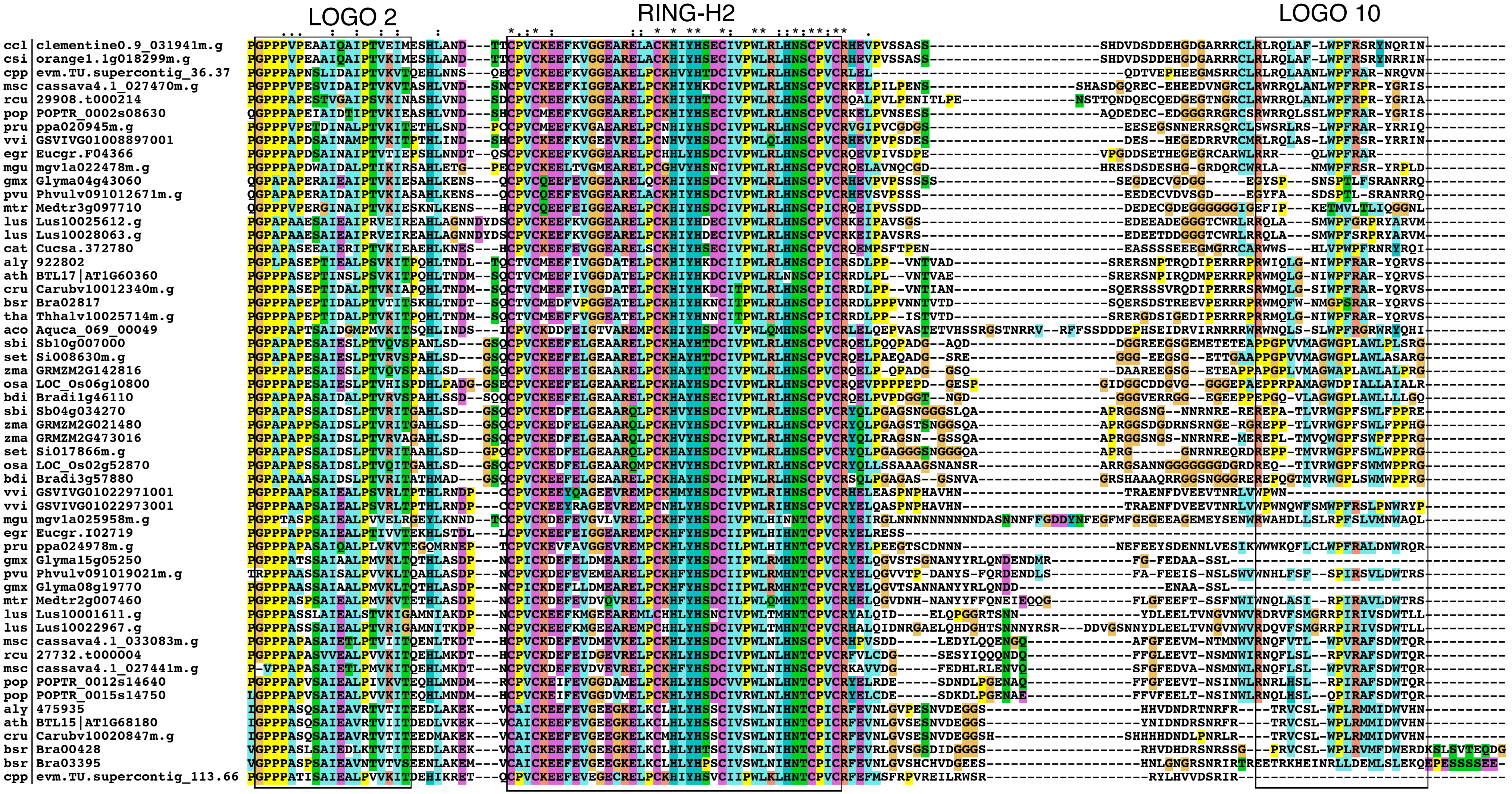

```
ccl|clementine0.9_031941m.g    ---------------------------------------------------------------------------PEG-DNIASSQ---------------------------------------------------------------------
csi|orange1.1g018299m.g        ---------------------------------------------------------------------------PEG-DNIAPSQAENSGWRHCGIL---------------------------------------------------------
cpp|evm.TU.supercontig_36.37   ---------------------------------------------------------------------------PQG-EDTGASQAQGNQSLSSV-----------------------------------------------------------
msc|cassava4.1_027470m.g       ---------------------------------------------------------------------------PHG-EHDGASQG--------------------------------------------------------------------
rcu|29908.t000214              ---------------------------------------------------------------------------PHGEDHVGTSQGGKALWKRRPLSQVVTGMPFSPSSLSSLVITNSQQSPFPLPIL--------------------------
pop|POPTR_0002s08630           ---------------------------------------------------------------------------PHG-EVAGTSQGGEN-----------------------------------------------------------------
pru|ppa020945m.g               ---------------------------------------------------------------------------PQG-DNEQHSAATSASRVG-------------------------------------------------------------
vvi|GSVIVG01008897001          ---------------------------------------------------------------------------PQD-RDVTPHRGES------------------------------------------------------------------
egr|Eucgr.F04366               ----------------------------------------------------------------------------HGQSPPPANSWWNSCSIL-------------------------------------------------------------
mgu|mgv1a022478m.g             ---------------------------------------------------------------------------SHGEGPAASSQHHGEH----------------------------------------------------------------
gmx|Glyma04g43060              ---------------------------------------------------------------------------PSCCIL--------------------------------------------------------------------------
pvu|Phvulv091012671m.g         ---------------------------------------------------------------------------PSCCIL--------------------------------------------------------------------------
mtr|Medtr3g097710              ---------------------------------------------------------------------------AAFCNPSKDLLQLEA-----------------------------------------------------------------
lus|Lus10025612.g              ---------------------------------------------------------------------------PNESVPANDNPNPNAPRRGRN-----------------------------------------------------------
lus|Lus10028063.g              ---------------------------------------------------------------------------PNDSVLANDNANPNAPRRGWN-----------------------------------------------------------
cat|Cucsa.372780               ---------------------------------------------------------------------------SPFPRNRFDASSRGM-----------------------------------------------------------------
aly|922802                     ---------------------------------------------------------------------------PEETTNQNPRSTRS------------------------------------------------------------------
ath|BTL17|AT1G60360            ---------------------------------------------------------------------------PEETANQNPRDNRS------------------------------------------------------------------
cru|Carubv10012340m.g          ---------------------------------------------------------------------------PEERAHQNPRGNRS------------------------------------------------------------------
bsr|Bra02817                   ---------------------------------------------------------------------------PEETAHRNTRGNRS------------------------------------------------------------------
tha|Thhalv10025714m.g          ---------------------------------------------------------------------------PEETAHRNPRGNRS------------------------------------------------------------------
aco|Aquca_069_00049            ---------------------------------------------------------------------------GTRDNNVNTNRGANSWWRSWFLL---------------------------------------------------------
sbi|Sb10g007000                ---------------------------------------------------------------------------PDGDGRV-------RNEANDGDGDGDADAAGGGSCAPAILQSFVVVAACFVVLSFFV-----------------------
set|Si008630m.g                ---------------------------------------------------------------------------PDGDAWE-------RSEADDGD------AAGGGTCAAAVMQSFVVVAACFFALSFFV-----------------------
zma|GRMZM2G142816              ---------------------------------------------------------------------------PDGDGLA-------RNEPDDGD------ARRSGVCAPAILQSFAVVAACFFILSFLV-----------------------
osa|LOC_Os06g10800             ---------------------------------------------------------------------------PDLNGWENSHG-RSESEADDDE------VAGGGVSTTAMIHSFFVVAACFLFISFLV-----------------------
bdi|Bradi1g46110               ---------------------------------------------------------------------------MGADGLEHRHGGRERGEADADD-----AGGNGGSSAPTILQSFVLVAACFLFASFLL-----------------------
sbi|Sb04g034270                ---------------------------------------------------------------------------MDDPDDH----AWEHERRGR------HDAADAG--------AFYAWWRSLFLI---------------------------
zma|GRMZM2G021480              ---------------------------------------------------------------------------MDDPDD-----AWEHGRRGR------HDAADAG--------AFYAWWRSLFLI---------------------------
zma|GRMZM2G473016              ---------------------------------------------------------------------------MDDPGD-----AWEHGQRGR------HDAADAG--------AFYAWWRSLFFI---------------------------
set|Si017866m.g                ---------------------------------------------------------------------------LDDPDDD----AWEHGRRRRQ-----HDVADAG--------AFYAWWRSLFLI---------------------------
osa|LOC_Os02g52870             ---------------------------------------------------------------------------LEDPDD-----GWEYGRRGR------PEAGDAG--------AFYAWWRSLFLF---------------------------
bdi|Bradi3g57880               ---------------------------------------------------------------------------MEDPDD-----EWEYGRHGHGRGHGRPEAGDAGGNDLSALQSFVLVATCVFFFSFFA-----------------------
vvi|GSVIVG01022971001          -----------------------------------------------------------------------------------------------------------------------------------------------------------
vvi|GSVIVG01022973001          ---------------------------------------------------------------------------DGLDFHYNRRGGETQNHAFFFLIFLKIMKDFKFLCKQNPLYLLTNNELKCAVSCSWHLVAFLAHSIACICMLQQHSPCGE
mgu|mgv1a025958m.g             ---------------------------------------------------------------------------CLDFLDDRINVSRGGN----------------------------------------------------------------
egr|Eucgr.I02719               -----------------------------------------------------------------------------------------------------------------------------------------------------------
pru|ppa024978m.g               ---------------------------------------------------------------------------YLDNQQ--------------------------------------------------------------------------
gmx|Glyma15g05250              -----------------------------------------------------------------------------------------------------------------------------------------------------------
pvu|Phvulv091019021m.g         ---------------------------------------------------------------------------YFDFHENRLRGREGRSWWRSLFIIE-------------------------------------------------------
gmx|Glyma08g19770              -----------------------------------------------------------------------------------------------------------------------------------------------------------
mtr|Medtr2g007460              ---------------------------------------------------------------------------YFELHAR--ARSRSSAWWRALLIS--------------------------------------------------------
lus|Lus10001611.g              ---------------------------------------------------------------------------CMEMLDRRIIGLINTNDDHYFSTGATEINDESGGSRWWPSWLIL------------------------------------
lus|Lus10022967.g              ---------------------------------------------------------------------------CMEMLDRRMVGLINTTDG---ATGDLGLKF--------------------------------------------------
msc|cassava4.1_033083m.g       ---------------------------------------------------------------------------YLEFLDNR-AASSRGDCKDEKVLKLN------------------------------------------------------
rcu|27732.t000004              ---------------------------------------------------------------------------YLDFLDSRPVATSRRDCKDEKLLKLNRFLQVGSNDMMMPFSNFRITEDGR------------------------------
msc|cassava4.1_027441m.g       ---------------------------------------------------------------------------SLDFLDNR-VSSSSG-----------------------------------------------------------------
pop|POPTR_0012s14640           ---------------------------------------------------------------------------YLDFLDSR-LATSNGARSWWRSWLIL------------------------------------------------------
pop|POPTR_0015s14750           ---------------------------------------------------------------------------YLDFLDSR-LATSRG-----------------------------------------------------------------
aly|475935                     -----------------------LIG-------------------------------------------------KIPPLDITFMAFLLCF---YVF----------------------------------------------------------
ath|BTL15|AT1G68180            -----------------------LIG-------------------------------------------------KIPPPDIAFMAFLLCL---YGF----------------------------------------------------------
cru|Carubv10020847m.g          -----------------------LIG-------------------------------------------------KIPSPDLTFMAFLLCF---YVF----------------------------------------------------------
bsr|Bra00428                   PSPLELPKQSEERLVHLSQTGKYLIVDFSHLSSSSTLGDSYWECKRPFNKNNQLWDQNLQDIGVCEDTICDDHDFHIPDIDLTFRNFEELFGADYDLVADDNNILFKGTLHQPICTRQNAISRLKEKKRARA-----------------------
bsr|Bra03395                   -AQAVTPWVVSDSVERLTSPPWRLLRTQGSSSNGQNYRMSTRSGRPFSAICWSTLEPVKPRAG------------SIIGANFTAGFVAGAATCPLDVAKTRRQIEKDRDRAMKMTTRQTLAEIWRYELAKGNSFLVDISKK--------------
cpp|evm.TU.supercontig_113.66  ---------------------------------------------------------------------------VNSNEGKFLMFSSSS-----F----------------------------------------------------------
```

Group F

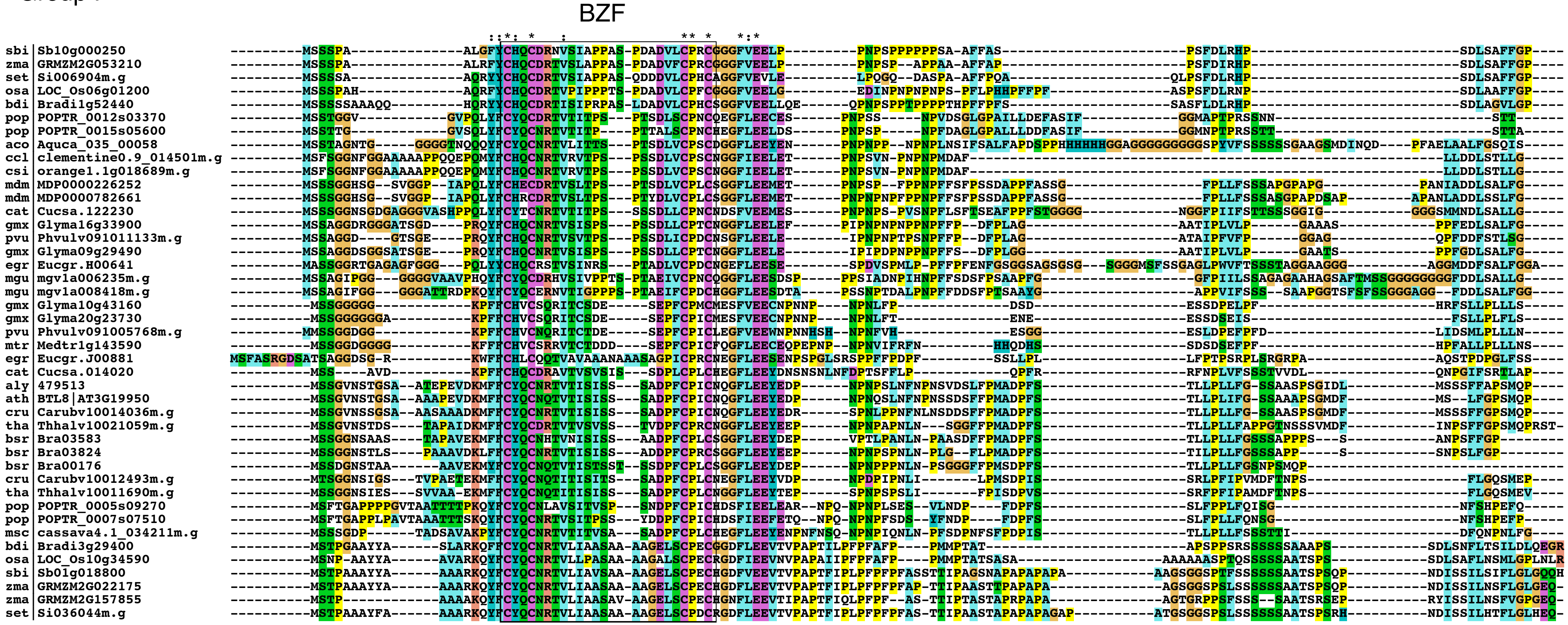
BZF
sbi Sb10g000250
zma GRMZM2G053210
set Si006904m.g
osa LOC_Os06g01200
bdi Bradi1g52440
pop POPTR_0012s03370
pop POPTR_0015s05600
aco Aquca_035_00058
ccl clementine0.9_014501m.g
csi orange1.1g018689m.g
mdm MDP0000226252
mdm MDP0000782661
cat Cucsa.122230
gmx Glyma16g33900
pvu Phvulv091011133m.g
gmx Glyma09g29490
egr Eucgr.H00641
mgu mgv1a006235m.g
mgu mgv1a008418m.g
gmx Glyma10g43160
gmx Glyma20g23730
pvu Phvulv091005768m.g
mtr Medtr1g143590
egr Eucgr.J00881
cat Cucsa.014020
aly 479513
ath BTL8|AT3G19950
cru Carubv10014036m.g
tha Thhalv10021059m.g
bsr Bra03583
bsr Bra03824
bsr Bra00176
cru Carubv10012493m.g
tha Thhalv10011690m.g
pop POPTR_0005s09270
pop POPTR_0007s07510
msc cassava4.1_034211m.g
bdi Bradi3g29400
osa LOC_Os10g34590
sbi Sb01g018800
zma GRMZM2G022175
zma GRMZM2G157855
set Si036044m.g

Group F (continued)

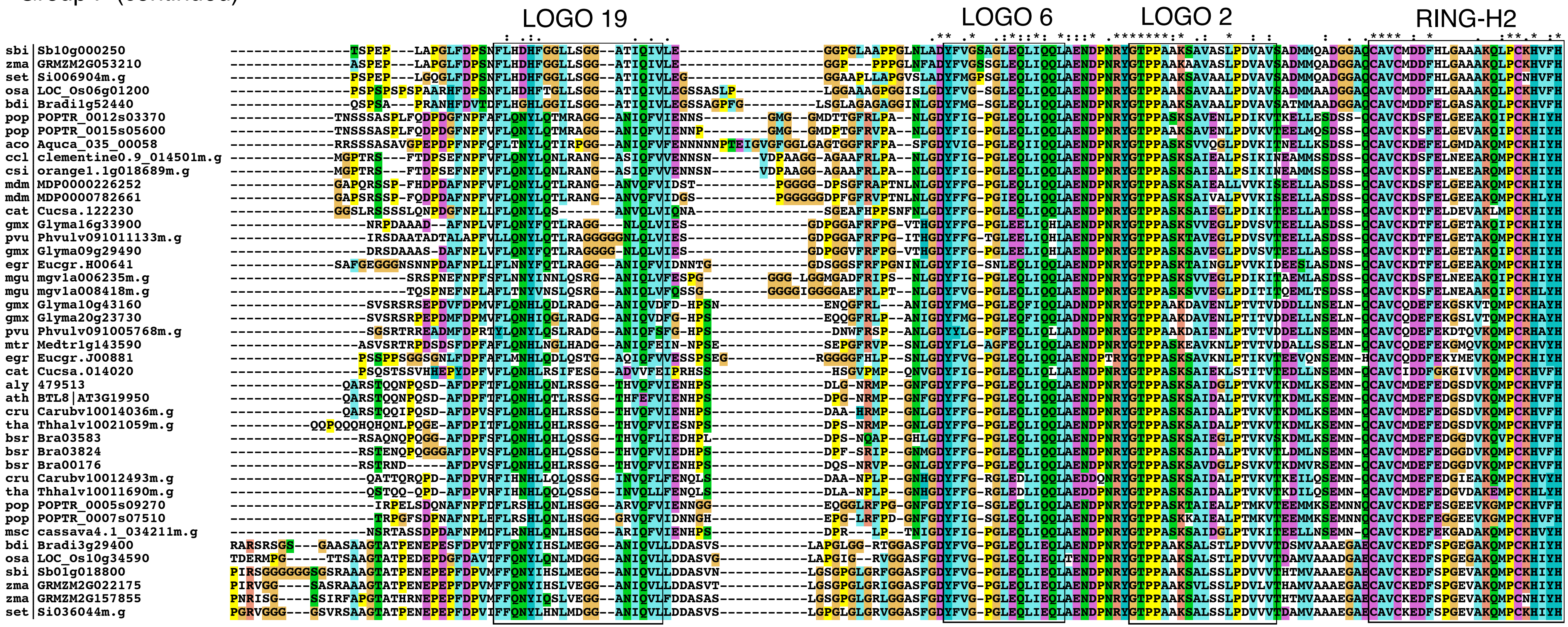

Group F (continued)

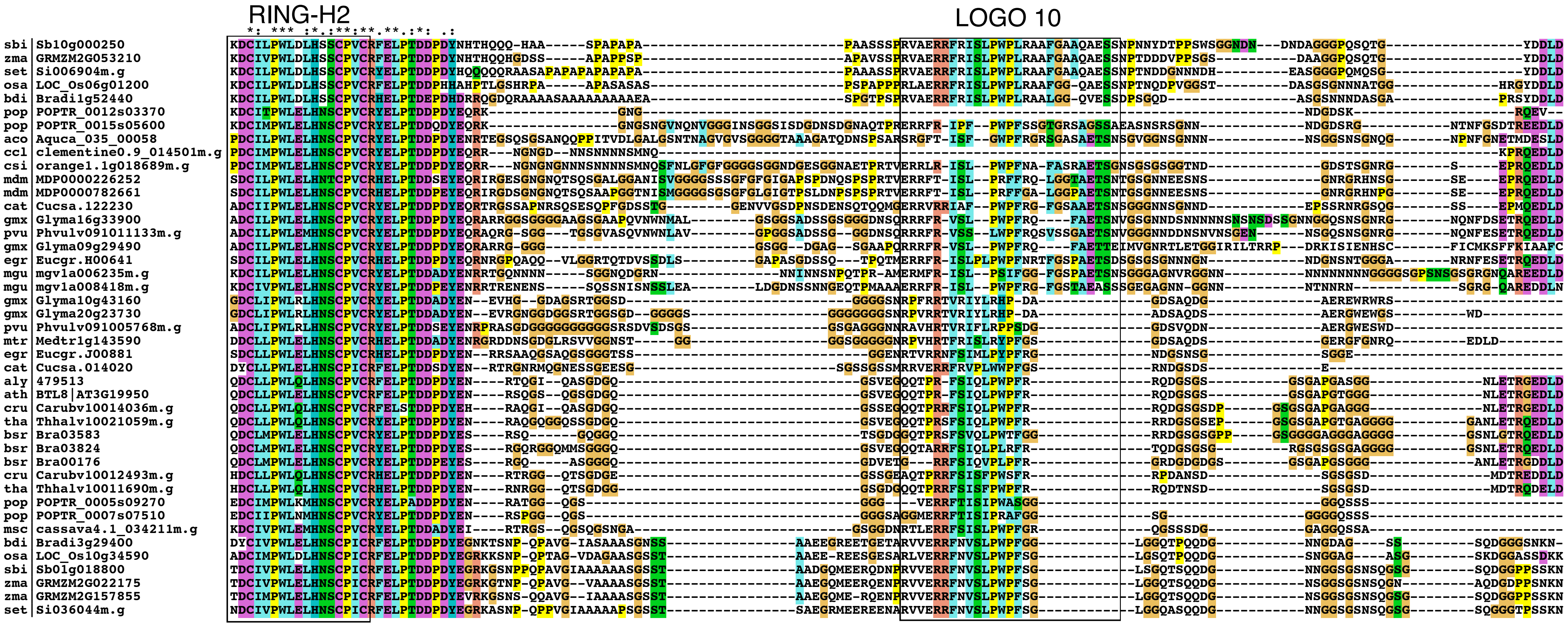

Supplement: Figure S3 — The sequence alignments were performed using ClustalX 2.0.12; default colors were used. Regions encompassing sequence LOGOs are enclosed by rectangles. (PDF) [file pone.0072729.s003.pdf]
