# Supplementary material for: Expansion and Diversification of BTL Ring-H2 Ubiquitin Ligases in Angiosperms: Putative Rabring7/BCA2 Orthologs
Source: PLoS One. 2013 Aug 8;8(8):e72729. doi: 10.1371/journal.pone.0072729 (PMC3738576; doi:10.1371/journal.pone.0072729)

Figure S4. Domain architecture of Rabring7/BCA2s based on sequence LOGOs depicted next to the phylogenetic tree.

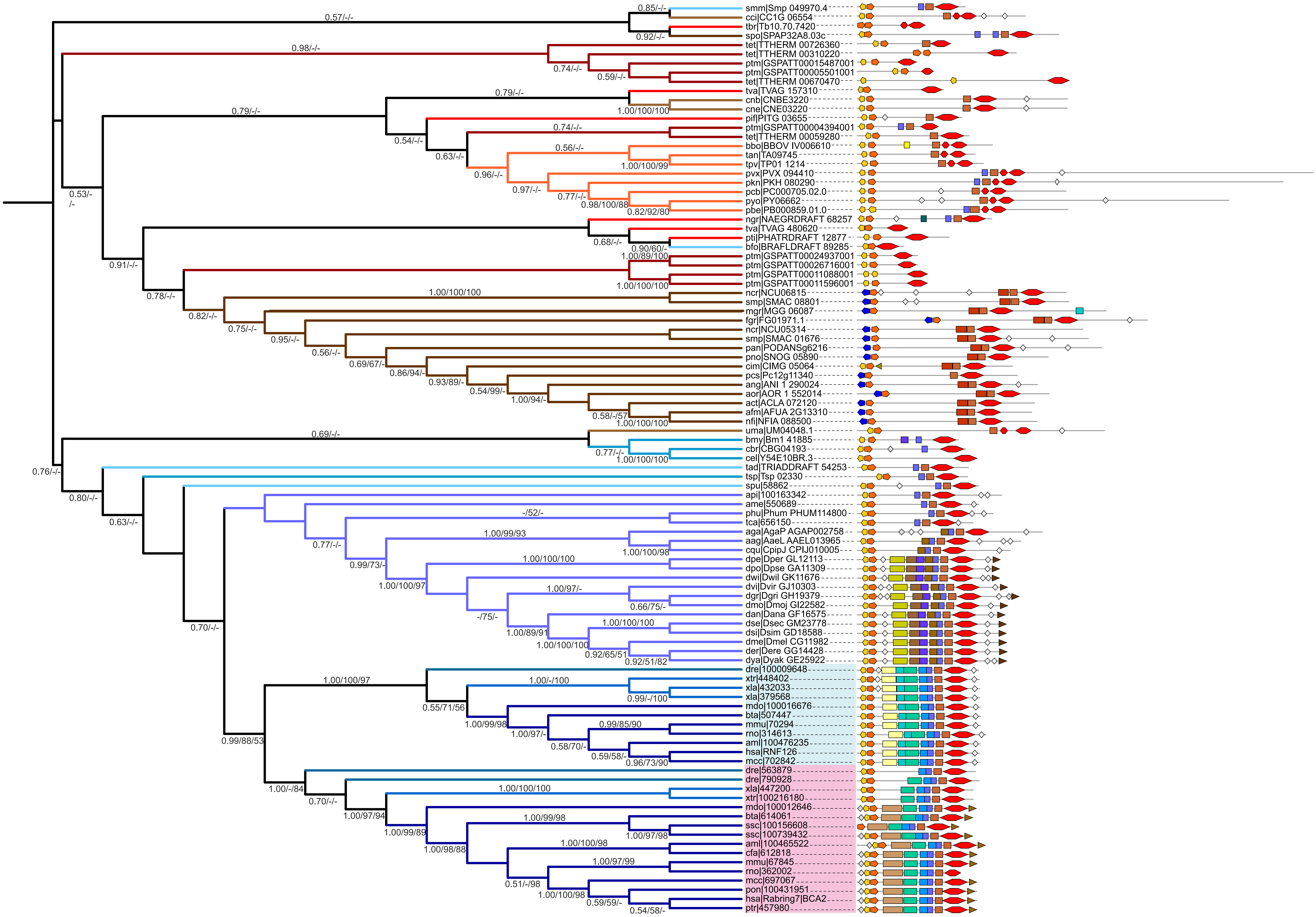

Supplement: Figure S4 — Domain architecture of Rabring7/BCA2s based on sequence LOGOs depicted next to the phylogenetic tree. (PDF) [file pone.0072729.s004.pdf]
