# Supplementary material for: Expansion and Diversification of BTL Ring-H2 Ubiquitin Ligases in Angiosperms: Putative Rabring7/BCA2 Orthologs
Source: PLoS One. 2013 Aug 8;8(8):e72729. doi: 10.1371/journal.pone.0072729 (PMC3738576; doi:10.1371/journal.pone.0072729)
Supplement: Table S1 — The color code of the species is the same as in Figure 2. (PDF) [file pone.0072729.s006.pdf]

Table S1. Name and abbreviation of species used in this work.

	Specie	Abbreviation
Chlorophyte	*Chlamydomonas reinhardtii*	cre
	Volvox carteri	vcn
	Ostreococcus lucimarinus	olu
	Ostreococcus tauri	ota
Bryophyta (Basal Plant)	*Physcomitrella patens*	ppp
Lycophyta (Basal Plant)	*Selaginella moellendorfii*	smo
Monocots	*Oryza sativa*	osa
	Brachypodium distachyon	bdi
	Setaria italica	set
	Zea mays	zma
	Sorghum bicolor	sbi
Eudicots	*Aquilegia coerulea*	aco
	Mimulus guttatus	mgu
	Vitis vinifera	vvi
	Eucalyptus grandis	egr
	Citrus clementina	ccl
	Citrus sinensis	csi
	Carica papaya	cpp
	Thellungiella halophila	tha
	Brassica rapa	bsr
	Capsella rubella	cru
	Arabidopsis lyrata	aly
	Arabidopsis thaliana	ath
	Prunus persica	pru
	Malus domestica	mdm
	Cucumis sativus	cat
	Glycine max	gmx
	Phaseolus vulgaris	pvu
	Medicago truncatula	mtr
	Populus trichocarpa	pop
	Linum usitatissimum	lus
	Ricinus communis	rcu
	Manihot esculenta	msc
Placozoans (Basal Animal)	*Trichoplax adhaerens*	tad
Flatworms (Basal Animal)	*Schistosoma mansoni*	smm
Lancelets (Basal Animal)	*Branchiostoma floridae*	bfo
Echinoderms (Basal Animal)	*Strongylocentrotus purpuratus*	spu
Mammals	*Ailuropoda melanoleuca*	aml
	Bos Taurus	bta
	Canis familiaris	cfa
	Homo sapiens	hsa
	Macaca mulatta	mcc
	Monodelphis domestica	mdo
	Mus musculus	mmu
	Pongo abelii	pon
	Pan troglodytes	ptr
	Rattus norvegicus	rno
	Sus scrofa	ssc
Fishes	*Danio rerio*	dre
Anphibians	*Xenopus laevis*	xla
	Xenopus tropicalis	xtr
Insects	*Aedes aegypti*	aag
	Anopheles gambiae	aga
	Apis mellifera	ame
	Acyrthosiphon pisum	api
	Culex quinquefasciatus	cqu

Table S1. (continued).

	Pediculus humanus	**phu**
	Tribolium castaneum	**tca**
	Drosophila ananassae	**dan**
	Drosophila erecta	**der**
	Drosophila grimshawi	**dgr**
	Drosophila melanogaster	**dme**
	Drosophila mojavensis	**dmo**
	Drosophila persimilis	**dpe**
	Drosophila pseudoobscura	**dpo**
	Drosophila sechellia	**dse**
	Drosophila simulans	**dsi**
	Drosophila virilis	**dvi**
	Drosophila willistoni	**dwi**
	Drosophila yakuba	**dya**
Nematodes	*Brugia malayi*	**bmy**
	Caenorhabditis briggsae	**cbr**
	Caenorhabditis elegans	**cel**
	Trichinella spiralis	**tsp**
Basidiomycetes	*Cryptococcus neoformans* JEC21	**cnb**
	Cryptococcus neoformans B-3501A	**cne**
	Ustilago maydis	**uma**
	Coprinopsis cinerea	**cci**
Taphrinomycetes (Ascomycetes)	*Schizosaccharomyces pombe*	**spo**
Eurotiomycetes (Ascomycetes)	*Phaeosphaeria nodorum*	**pno**
	Aspergillus clavatus	**act**
	Aspergillus fumigatus	**afm**
	Aspergillus niger	**ang**
	Aspergillus oryzae	**aor**
	Coccidioides immitis	**cim**
	Neosartorya fischeri	**nfi**
	Penicillium chrysogenum	**pcs**
Sordariomycetes (Ascomycetes)	*Fusarium graminearum*	**fgr**
	Magnaporthe oryzae	**mgr**
	Neurospora crassa	**ncr**
	Sordaria macrospora	**smp**
	Podospora anserine	**pan**
Apicomplexans (Protists)	*Babesia bovis*	**bbo**
	Plasmodium berghei	**pbe**
	Plasmodium chabaudi	**pcb**
	Plasmodium knowles	**pkn**
	Plasmodium vivax	**pvx**
	Plasmodium yoelii	**pyo**
	Theileria annulata	**tan**
	Theileria parva	**tpv**
Ciliates (Protist)	*Paramecium tetraurelia*	**ptm**
	Tetrahymena thermophila	**tet**
Other protist (Protist)	*Naegleria gruberi*	**ngr**
	Phaeodactylum tricornutum	**pti**
	Trypanosoma brucei	**tbr**
	Phytophthora infestans	**pif**
	Trichomonas vaginalis	**tva**
