# Supplementary material for: Expansion and Diversification of BTL Ring-H2 Ubiquitin Ligases in Angiosperms: Putative Rabring7/BCA2 Orthologs
Source: PLoS One. 2013 Aug 8;8(8):e72729. doi: 10.1371/journal.pone.0072729 (PMC3738576; doi:10.1371/journal.pone.0072729)
Supplement: Table S2 — List of retrieved genes from animals, fungibold>, protists and plants. (PDF) [file pone.0072729.s007.pdf]

Table S2. List of retrieved genes from animals, fungi, protist and plants.

Specie	Locus	GenBank/protein accession no.
Naegleria gruberi	ngr|NAEGRDRAFT_68257	EFC43818
Trichomonas vaginalis	tva|TVAG_157310	EAY10654
	tva|TVAG_480620	EAX94245
Trypanosoma brucei	tbr|Tb10.70.7420	EAN77508
Phytophthora infestans	pif|PITG_03655	EEY66113
Phaeodactylum tricornutum	pti|PHATRDRAFT_12877	EEC47891
Tetrahymena thermophila	tet|TTHERM_00059280	EAR87415
	tet|TTHERM_00310220	EAS00853
	tet|TTHERM_00670470	EAS06123
	tet|TTHERM_00726360	EAS06916
Paramecium tetraurelia	ptm|GSPATT00004394001	CAK56300
	ptm|GSPATT00005501001	CAK62255
	ptm|GSPATT00011088001	CAK75456
	ptm|GSPATT00011596001	CAK75995
	ptm|GSPATT00015487001	CAK80610
	ptm|GSPATT00024937001	CAK92205
	ptm|GSPATT00026716001	CAK92205
Babesia bovis	bbo|BBOV_IV006610	EDO07017
Theileria parva	tpv|TP01_1214	EAN34452
Theileria annulata	tan|TA09745	CAI72880
Plasmodium vivax	pvx|PVX_094410	EDL44623
Plasmodium knowles	pkn|PKH_080290	CAQ39443
Plasmodium yoelii	pyo|PY06662	EAA18913
Plasmodium chabaudi	pcb|PC000705.02.0	CAH78018
Plasmodium berghei	pbe|PB000859.01.0	CAH96534
Ostreococcus lucimarinus	olu|OSTLU_31341	ABO95627
Ostreococcus tauri	ota|Ot04g04330	CAL51831
Volvox carteri	vcn|VOLCADRAFT_100265	CAL51831
Chlamydomonas reinhardtii	cre|Cre10.g422050	Cre10.g422050
Physcomitrella patens	ppp|Pp1s161_109V6	Pp1s161_109V6
	ppp|Pp1s223_128V6	Pp1s223_128V6
	ppp|Pp1s37_80V6	Pp1s37_80V6
Selaginella moellendorfii	smo|18753	18753
	smo|18755	18755
	smo|18758	18758
	smo|8109	8109
Oryza sativa	osa|LOC_Os01g16950	LOC_Os01g16950
	osa|LOC_Os01g58780	LOC_Os01g58780
	osa|LOC_Os01g74040	LOC_Os01g74040
	osa|LOC_Os02g52870	LOC_Os02g52870
	osa|LOC_Os03g16480	LOC_Os03g16480
	osa|LOC_Os03g20870	LOC_Os03g20870
	osa|LOC_Os03g22830	LOC_Os03g22830
	osa|LOC_Os03g59760	LOC_Os03g59760
	osa|LOC_Os05g01940	LOC_Os05g01940
	osa|LOC_Os05g40980	LOC_Os05g40980
	osa|LOC_Os05g41520	LOC_Os05g41520
	osa|LOC_Os06g01200	LOC_Os06g01200
	osa|LOC_Os06g10800	LOC_Os06g10800
	osa|LOC_Os08g36170	LOC_Os08g36170
	osa|LOC_Os10g34590	LOC_Os10g34590
	osa|LOC_Os11g02670	LOC_Os11g02670
	osa|LOC_Os12g02620	LOC_Os12g02620

Table S2. (continued).

Brachypodium distachyon	bdi|Bradi1g04180	Bradi1g04180
	bdi|Bradi1g46110	Bradi1g46110
	bdi|Bradi1g52440	Bradi1g52440
	bdi|Bradi1g62350	Bradi1g62350
	bdi|Bradi1g63720	Bradi1g63720
	bdi|Bradi1g66780	Bradi1g66780
	bdi|Bradi2g04010	Bradi2g04010
	bdi|Bradi2g10520	Bradi2g10520
	bdi|Bradi2g21870	Bradi2g21870
	bdi|Bradi2g39220	Bradi2g39220
	bdi|Bradi2g52600	Bradi2g52600
	bdi|Bradi3g29400	Bradi3g29400
	bdi|Bradi3g37800	Bradi3g37800
	bdi|Bradi3g57880	Bradi3g57880
	bdi|Bradi4g44277	Bradi4g44277
Setaria italica	set|Si001919m.g	Si001919m.g
	set|Si002234m.g	Si002234m.g
	set|Si004415m.g	Si004415m.g
	set|Si006904m.g	Si006904m.g
	set|Si008630m.g	Si008630m.g
	set|Si009875m.g	Si009875m.g
	set|Si010014m.g	Si010014m.g
	set|Si013915m.g	Si013915m.g
	set|Si017866m.g	Si017866m.g
	set|Si022618m.g	Si022618m.g
	set|Si025925m.g	Si025925m.g
	set|Si036044m.g	Si036044m.g
	set|Si036169m.g	Si036169m.g
	set|Si036686m.g	Si036686m.g
	set|Si036914m.g	Si036914m.g
	set|Si036971m.g	Si036971m.g
Zea mays	zma|AC213654.3_FG001	AC213654.3_FG001
	zma|GRMZM2G021480	GRMZM2G021480
	zma|GRMZM2G022175	GRMZM2G022175
	zma|GRMZM2G027120	GRMZM2G027120
	zma|GRMZM2G045084	GRMZM2G045084
	zma|GRMZM2G049346	GRMZM2G049346
	zma|GRMZM2G053210	GRMZM2G053210
	zma|GRMZM2G124441	GRMZM2G124441
	zma|GRMZM2G142816	GRMZM2G142816
	zma|GRMZM2G157246	GRMZM2G157246
	zma|GRMZM2G157855	GRMZM2G157855
	zma|GRMZM2G164358	GRMZM2G164358
	zma|GRMZM2G176028	GRMZM2G176028
	zma|GRMZM2G300589	GRMZM2G300589
	zma|GRMZM2G305264	GRMZM2G305264
	zma|GRMZM2G417125	GRMZM2G417125
	zma|GRMZM2G473016	GRMZM2G473016
	zma|GRMZM2G567897	GRMZM2G567897
	zma|GRMZM5G828820	GRMZM5G828820
	zma|GRMZM5G886096	GRMZM5G886096
Sorghum bicolor	sbi|Sb01g003740	Sb01g003740
	sbi|Sb01g018800	Sb01g018800
	sbi|Sb01g035310	Sb01g035310

Table S2. (continued).

	sbi|Sb01g036700	Sb01g036700
	sbi|Sb01g039760	Sb01g039760
	sbi|Sb03g011120	Sb03g011120
	sbi|Sb04g034270	Sb04g034270
	sbi|Sb05g001320	Sb05g001320
	sbi|Sb07g022600	Sb07g022600
	sbi|Sb09g001100	Sb09g001100
	sbi|Sb09g023840	Sb09g023840
	sbi|Sb10g000250	Sb10g000250
	sbi|Sb10g007000	Sb10g007000
Aquilegia coerulea	aco|Aquca_004_00514	Aquca_004_00514
	aco|Aquca_009_00137	Aquca_009_00137
	aco|Aquca_009_00930	Aquca_009_00930
	aco|Aquca_009_00931	Aquca_009_00931
	aco|Aquca_014_00932	Aquca_014_00932
	aco|Aquca_035_00058	Aquca_035_00058
	aco|Aquca_069_00049	Aquca_069_00049
Mimulus guttatus	mgu|mgv1a006235m.g	mgv1a006235m.g
	mgu|mgv1a006570m.g	mgv1a006570m.g
	mgu|mgv1a007795m.g	mgv1a007795m.g
	mgu|mgv1a008418m.g	mgv1a008418m.g
	mgu|mgv1a008674m.g	mgv1a008674m.g
	mgu|mgv1a010352m.g	mgv1a010352m.g
	mgu|mgv1a010758m.g	mgv1a010758m.g
	mgu|mgv1a022217m.g	mgv1a022217m.g
	mgu|mgv1a022478m.g	mgv1a022478m.g
	mgu|mgv1a024323m.g	mgv1a024323m.g
	mgu|mgv1a025958m.g	mgv1a025958m.g
Vitis vinifera	vvi|GSVIVG01008897001	GSVIVG01008897001
	vvi|GSVIVG01022971001	GSVIVG01022971001
	vvi|GSVIVG01022973001	GSVIVG01022973001
	vvi|GSVIVG01024920001	GSVIVG01024920001
	vvi|GSVIVG01025505001	GSVIVG01025505001
	vvi|GSVIVG01034134001	GSVIVG01034134001
	vvi|GSVIVG01037379001	GSVIVG01037379001
Eucalyptus grandis	egr|Eucgr.A01286	Eucgr.A01286
	egr|Eucgr.A02079	Eucgr.A02079
	egr|Eucgr.B03585	Eucgr.B03585
	egr|Eucgr.B03765	Eucgr.B03765
	egr|Eucgr.F04366	Eucgr.F04366
	egr|Eucgr.G02948	Eucgr.G02948
	egr|Eucgr.H00641	Eucgr.H00641
	egr|Eucgr.H00970	Eucgr.H00970
	egr|Eucgr.I02719	Eucgr.I02719
	egr|Eucgr.J00661	Eucgr.J00661
	egr|Eucgr.J00881	Eucgr.J00881
	egr|Eucgr.J01748	Eucgr.J01748
	egr|Eucgr.J02036	Eucgr.J02036
	egr|Eucgr.K01302	Eucgr.K01302
	egr|Eucgr.K01683	Eucgr.K01683
Citrus clementina	ccl|clementine0.9_012040m.g	clementine0.9_012040m.g
	ccl|clementine0.9_013216m.g	clementine0.9_013216m.g
	ccl|clementine0.9_013454m.g	clementine0.9_013454m.g
	ccl|clementine0.9_013794m.g	clementine0.9_013794m.g

Table S2. (continued).

	ccl|clementine0.9_014425m.g	clementine0.9_014425m.g
	ccl|clementine0.9_014501m.g	clementine0.9_014501m.g
	ccl|clementine0.9_016541m.g	clementine0.9_016541m.g
	ccl|clementine0.9_023238m.g	clementine0.9_023238m.g
	ccl|clementine0.9_030301m.g	clementine0.9_030301m.g
	ccl|clementine0.9_031941m.g	clementine0.9_031941m.g
Citrus sinensis	csi|orange1.1g009095m.g	orange1.1g009095m.g
	csi|orange1.1g017208m.g	orange1.1g017208m.g
	csi|orange1.1g017269m.g	orange1.1g017269m.g
	csi|orange1.1g017955m.g	orange1.1g017955m.g
	csi|orange1.1g018299m.g	orange1.1g018299m.g
	csi|orange1.1g018689m.g	orange1.1g018689m.g
	csi|orange1.1g021656m.g	orange1.1g021656m.g
	csi|orange1.1g036250m.g	orange1.1g036250m.g
	csi|orange1.1g043965m.g	orange1.1g043965m.g
	csi|orange1.1g045745m.g	orange1.1g045745m.g
Carica papaya	cpp|evm.TU.supercontig_1036.1	evm.TU.supercontig_1036.1
	cpp|evm.TU.supercontig_113.66	evm.TU.supercontig_113.66
	cpp|evm.TU.supercontig_119.24	evm.TU.supercontig_119.24
	cpp|evm.TU.supercontig_1460.1	evm.TU.supercontig_1460.1
	cpp|evm.TU.supercontig_169.13	evm.TU.supercontig_169.13
	cpp|evm.TU.supercontig_19.287	evm.TU.supercontig_19.287
	cpp|evm.TU.supercontig_26.313	evm.TU.supercontig_26.313
	cpp|evm.TU.supercontig_34.207	evm.TU.supercontig_34.207
	cpp|evm.TU.supercontig_36.37	evm.TU.supercontig_36.37
	cpp|evm.TU.supercontig_39.6	evm.TU.supercontig_39.6
	cpp|evm.TU.supercontig_51.88	evm.TU.supercontig_51.88
	cpp|evm.TU.supercontig_81.109	evm.TU.supercontig_81.109
Thellungiella halophila	tha|Thhalv10002566m.g	Thhalv10002566m.g
	tha|Thhalv10005668m.g	Thhalv10005668m.g
	tha|Thhalv10006102m.g	Thhalv10006102m.g
	tha|Thhalv10006114m.g	Thhalv10006114m.g
	tha|Thhalv10011690m.g	Thhalv10011690m.g
	tha|Thhalv10012351m.g	Thhalv10012351m.g
	tha|Thhalv10013762m.g	Thhalv10013762m.g
	tha|Thhalv10013831m.g	Thhalv10013831m.g
	tha|Thhalv10015834m.g	Thhalv10015834m.g
	tha|Thhalv10016765m.g	Thhalv10016765m.g
	tha|Thhalv10016932m.g	Thhalv10016932m.g
	tha|Thhalv10021059m.g	Thhalv10021059m.g
	tha|Thhalv10021230m.g	Thhalv10021230m.g
	tha|Thhalv10021967m.g	Thhalv10021967m.g
	tha|Thhalv10025560m.g	Thhalv10025560m.g
	tha|Thhalv10025714m.g	Thhalv10025714m.g
Brassica rapa	bsr|Bra00014	Bra00014
	bsr|Bra00020	Bra00020
	bsr|Bra00035	Bra00035
	bsr|Bra00138	Bra00138
	bsr|Bra00154	Bra00154
	bsr|Bra00176	Bra00176
	bsr|Bra00255	Bra00255
	bsr|Bra00282	Bra00282
	bsr|Bra00428	Bra00428
	bsr|Bra00458	Bra00458

Table S2. (continued).

	bsr|Bra00502	Bra00502
	bsr|Bra00670	Bra00670
	bsr|Bra00725	Bra00725
	bsr|Bra00750	Bra00750
	bsr|Bra01426	Bra01426
	bsr|Bra01910	Bra01910
	bsr|Bra01938	Bra01938
	bsr|Bra02031	Bra02031
	bsr|Bra02644	Bra02644
	bsr|Bra02743	Bra02743
	bsr|Bra02817	Bra02817
	bsr|Bra02893	Bra02893
	bsr|Bra02986	Bra02986
	bsr|Bra03013	Bra03013
	bsr|Bra03085	Bra03085
	bsr|Bra03395	Bra03395
	bsr|Bra03583	Bra03583
	bsr|Bra03710	Bra03710
	bsr|Bra03780	Bra03780
	bsr|Bra03799	Bra03799
	bsr|Bra03824	Bra03824
Capsella rubella	cru|Carubv10005120m.g	Carubv10005120m.g
	cru|Carubv10009589m.g	Carubv10009589m.g
	cru|Carubv10012340m.g	Carubv10012340m.g
	cru|Carubv10012493m.g	Carubv10012493m.g
	cru|Carubv10012728m.g	Carubv10012728m.g
	cru|Carubv10014036m.g	Carubv10014036m.g
	cru|Carubv10014141m.g	Carubv10014141m.g
	cru|Carubv10014632m.g	Carubv10014632m.g
	cru|Carubv10016862m.g	Carubv10016862m.g
	cru|Carubv10017636m.g	Carubv10017636m.g
	cru|Carubv10017698m.g	Carubv10017698m.g
	cru|Carubv10020847m.g	Carubv10020847m.g
	cru|Carubv10023378m.g	Carubv10023378m.g
	cru|Carubv10023620m.g	Carubv10023620m.g
	cru|Carubv10024842m.g	Carubv10024842m.g
	cru|Carubv10026533m.g	Carubv10026533m.g
	cru|Carubv10028114m.g	Carubv10028114m.g
Arabidopsis lyrata	aly|317879	317879
	aly|324170	324170
	aly|337633	337633
	aly|475935	475935
	aly|478724	478724
	aly|479513	479513
	aly|483145	483145
	aly|483556	483556
	aly|485035	485035
	aly|486871	486871
	aly|492175	492175
	aly|495725	495725
	aly|496089	496089
	aly|496711	496711
	aly|922802	922802
	aly|934736	934736

Table S2. (continued).

	aly|936073	936073
	aly|939042	939042
Arabidopsis thaliana	ath|BTL1|AT2G40830	AT2G40830
	ath|BTL2|AT3G56580	AT3G56580
	ath|BTL3|AT3G10815	AT3G10815
	ath|BTL4|AT5G56340	AT5G56340
	ath|BTL5|AT4G26400	AT4G26400
	ath|BTL6|AT1G55530	AT1G55530
	ath|BTL7|AT3G13430	AT3G13430
	ath|BTL8|AT3G19950	AT3G19950
	ath|BTL9|AT5G59550	AT5G59550
	ath|BTL10|AT3G46620	AT3G46620
	ath|BTL11|AT2G39720	AT2G39720
	ath|BTL12|AT5G64920	AT5G64920
	ath|BTL13|AT3G60080	AT3G60080
	ath|BTL14|AT2G44330	AT2G44330
	ath|BTL15|AT1G68180	AT1G68180
	ath|BTL16|AT5G01980	AT5G01980
	ath|BTL17|AT1G60360	AT1G60360
Prunus persica	pru|ppa006881m.g	ppa006881m.g
	pru|ppa007335m.g	ppa007335m.g
	pru|ppa007829m.g	ppa007829m.g
	pru|ppa008198m.g	ppa008198m.g
	pru|ppa008742m.g	ppa008742m.g
	pru|ppa020945m.g	ppa020945m.g
	pru|ppa024978m.g	ppa024978m.g
Malus domestica	mdm|MDP0000226252	MDP0000226252
	mdm|MDP0000368098	MDP0000368098
	mdm|MDP0000675059	MDP0000675059
	mdm|MDP0000770377	MDP0000770377
	mdm|MDP0000782661	MDP0000782661
	mdm|MDP0000919900	MDP0000919900
Cucumis sativus	cat|Cucsa.014020	Cucsa.014020
	cat|Cucsa.049930	Cucsa.049930
	cat|Cucsa.122230	Cucsa.122230
	cat|Cucsa.130800	Cucsa.130800
	cat|Cucsa.138820	Cucsa.138820
	cat|Cucsa.142510	Cucsa.142510
	cat|Cucsa.152640	Cucsa.152640
	cat|Cucsa.165190	Cucsa.165190
	cat|Cucsa.358650	Cucsa.358650
	cat|Cucsa.372780	Cucsa.372780
Glycine max	gmx|Glyma0024s00230	Glyma0024s00230
	gmx|Glyma02g07820	Glyma02g07820
	gmx|Glyma02g22760	Glyma02g22760
	gmx|Glyma02g41650	Glyma02g41650
	gmx|Glyma02g44470	Glyma02g44470
	gmx|Glyma04g43060	Glyma04g43060
	gmx|Glyma08g16830	Glyma08g16830
	gmx|Glyma08g19770	Glyma08g19770
	gmx|Glyma09g29490	Glyma09g29490
	gmx|Glyma09g40770	Glyma09g40770
	gmx|Glyma10g43160	Glyma10g43160
	gmx|Glyma10g43280	Glyma10g43280

Table S2. (continued).

	gmx|Glyma11g14580	Glyma11g14580
	gmx|Glyma11g34160	Glyma11g34160
	gmx|Glyma12g06460	Glyma12g06460
	gmx|Glyma13g04080	Glyma13g04080
	gmx|Glyma13g04100	Glyma13g04100
	gmx|Glyma13g41340	Glyma13g41340
	gmx|Glyma14g04340	Glyma14g04340
	gmx|Glyma14g07300	Glyma14g07300
	gmx|Glyma15g04080	Glyma15g04080
	gmx|Glyma15g05250	Glyma15g05250
	gmx|Glyma15g42250	Glyma15g42250
	gmx|Glyma16g26840	Glyma16g26840
	gmx|Glyma16g33900	Glyma16g33900
	gmx|Glyma18g00300	Glyma18g00300
	gmx|Glyma18g40130	Glyma18g40130
	gmx|Glyma18g45040	Glyma18g45040
	gmx|Glyma20g23550	Glyma20g23550
	gmx|Glyma20g23730	Glyma20g23730
Phaseolus vulgaris	pvu|Phvulv091000738m.g	Phvulv091000738m.g
	pvu|Phvulv091001306m.g	Phvulv091001306m.g
	pvu|Phvulv091002571m.g	Phvulv091002571m.g
	pvu|Phvulv091005741m.g	Phvulv091005741m.g
	pvu|Phvulv091005768m.g	Phvulv091005768m.g
	pvu|Phvulv091005924m.g	Phvulv091005924m.g
	pvu|Phvulv091006877m.g	Phvulv091006877m.g
	pvu|Phvulv091007434m.g	Phvulv091007434m.g
	pvu|Phvulv091011133m.g	Phvulv091011133m.g
	pvu|Phvulv091012671m.g	Phvulv091012671m.g
	pvu|Phvulv091012838m.g	Phvulv091012838m.g
	pvu|Phvulv091012846m.g	Phvulv091012846m.g
	pvu|Phvulv091014314m.g	Phvulv091014314m.g
	pvu|Phvulv091015600m.g	Phvulv091015600m.g
	pvu|Phvulv091019021m.g	Phvulv091019021m.g
	pvu|Phvulv091021650m.g	Phvulv091021650m.g
	pvu|Phvulv091029702m.g	Phvulv091029702m.g
Medicago truncatula	mtr|Medtr1g143590	Medtr1g143590
	mtr|Medtr2g007460	Medtr2g007460
	mtr|Medtr2g117130	Medtr2g117130
	mtr|Medtr3g097710	Medtr3g097710
	mtr|Medtr3g105730	Medtr3g105730
	mtr|Medtr4g084220	Medtr4g084220
	mtr|Medtr5g087700	Medtr5g087700
	mtr|Medtr5g095810	Medtr5g095810
	mtr|Medtr6g030770	Medtr6g030770
	mtr|Medtr7g080710	Medtr7g080710
Populus trichocarpa	pop|POPTR_0001s03880	POPTR_0001s03880
	pop|POPTR_0001s23810	POPTR_0001s23810
	pop|POPTR_0001s25010	POPTR_0001s25010
	pop|POPTR_0002s08630	POPTR_0002s08630
	pop|POPTR_0003s20780	POPTR_0003s20780
	pop|POPTR_0005s09270	POPTR_0005s09270
	pop|POPTR_0006s03070	POPTR_0006s03070
	pop|POPTR_0006s08810	POPTR_0006s08810
	pop|POPTR_0006s11280	POPTR_0006s11280

Table S2. (continued).

	pop|POPTR_0007s07510	POPTR_0007s07510
	pop|POPTR_0008s05830	POPTR_0008s05830
	pop|POPTR_0009s03970	POPTR_0009s03970
	pop|POPTR_0010s20890	POPTR_0010s20890
	pop|POPTR_0012s03370	POPTR_0012s03370
	pop|POPTR_0012s14640	POPTR_0012s14640
	pop|POPTR_0013s05700	POPTR_0013s05700
	pop|POPTR_0015s05600	POPTR_0015s05600
	pop|POPTR_0015s14750	POPTR_0015s14750
	pop|POPTR_0016s02910	POPTR_0016s02910
	pop|POPTR_0016s14830	POPTR_0016s14830
	pop|POPTR_0019s04790	POPTR_0019s04790
Linum usitatissimum	lus|Lus10001611.g	Lus10001611.g
	lus|Lus10002637.g	Lus10002637.g
	lus|Lus10003698.g	Lus10003698.g
	lus|Lus10004712.g	Lus10004712.g
	lus|Lus10013235.g	Lus10013235.g
	lus|Lus10013397.g	Lus10013397.g
	lus|Lus10020258.g	Lus10020258.g
	lus|Lus10022967.g	Lus10022967.g
	lus|Lus10024372.g	Lus10024372.g
	lus|Lus10025612.g	Lus10025612.g
	lus|Lus10028063.g	Lus10028063.g
	lus|Lus10030755.g	Lus10030755.g
	lus|Lus10040278.g	Lus10040278.g
Ricinus communis	rcu|27568.t000014	27568.t000014
	rcu|27732.t000004	27732.t000004
	rcu|27955.t000012	27955.t000012
	rcu|28842.t000021	28842.t000021
	rcu|29568.t000007	29568.t000007
	rcu|29751.t000117	29751.t000117
	rcu|29908.t000214	29908.t000214
	rcu|29950.t000046	29950.t000046
	rcu|30170.t000615	30170.t000615
	rcu|30226.t000059	30226.t000059
Manihot esculenta	msc|cassava4.1_004531m.g	cassava4.1_004531m.g
	msc|cassava4.1_009150m.g	cassava4.1_009150m.g
	msc|cassava4.1_009285m.g	cassava4.1_009285m.g
	msc|cassava4.1_009387m.g	cassava4.1_009387m.g
	msc|cassava4.1_009645m.g	cassava4.1_009645m.g
	msc|cassava4.1_010411m.g	cassava4.1_010411m.g
	msc|cassava4.1_010647m.g	cassava4.1_010647m.g
	msc|cassava4.1_012645m.g	cassava4.1_012645m.g
	msc|cassava4.1_013264m.g	cassava4.1_013264m.g
	msc|cassava4.1_015095m.g	cassava4.1_015095m.g
	msc|cassava4.1_022114m.g	cassava4.1_022114m.g
	msc|cassava4.1_023055m.g	cassava4.1_023055m.g
	msc|cassava4.1_027441m.g	cassava4.1_027441m.g
	msc|cassava4.1_027470m.g	cassava4.1_027470m.g
	msc|cassava4.1_033083m.g	cassava4.1_033083m.g
	msc|cassava4.1_034211m.g	cassava4.1_034211m.g
Ustilago maydis	uma|UM04048.1	EAK84973
Coprinopsis cinerea	cci|CC1G_06554	EAU92543
Cryptococcus neoformans	cnb|CNBE3220	EAL20614

Table S2. (continued).

Cryptococcus neoformans	cne|CNE03220	AAW43896
Fusarium graminearum	fgr|FG01971.1	XP_382147
Magnaporthe oryzae	mgr|MGG_06087	EHA52145
Podospora anserine	pan|PODANSg6216	XP_001909181
Sordaria macrospora	smp|SMAC_01676	CCC08126
	smp|SMAC_08801	CCC10989
Neurospora crassa	ncr|NCU05314	EAA32691
	ncr|NCU06815	EAA34420
Schizosaccharomyces pombe	spo|SPAP32A8.03c	CAC29482
Coccidioides immitis	cim|CIMG_05064	EAS34323
Neosartorya fischeri	nfi|NFIA_088500	EAW18894
Penicillium chrysogenum	pcs|Pc12g11340	CAP80761
Aspergillus fumigatus	afm|AFUA_2G13310	EAL93626
Aspergillus oryzae	aor|AOR_1_552014	XP_001821770
Aspergillus niger	ang|ANI_1_290024	XP_001399359
Aspergillus clavatus	act|ACLA_072120	EAW14179
Phaeosphaeria nodorum	pno|SNOG_05890	EAT86954
Trichoplax adhaerens	tad|TRIADDRAFT_54253	EDV26352
Schistosoma mansoni	smm|Smp_049970.4	CCD59309
Strongylocentrotus purpuratus	spu|58862	XP_002608308
Branchiostoma floridae	bfo|BRAFLDRAFT_89285	XP_002608308
Danio rerio	dre|100009648	NP_001076486
	dre|563879	XP_003200594
	dre|790928	NP_001073542
Xenopus tropicalis	xtr|100216180	NP_001135621
	xtr|448402	NP_001006735
Xenopus laevis	xla|379568	NP_001079878
	xla|432033	NP_001084974
	xla|447200	NP_001087376
Monodelphis domestica	mdo|100012646	XP_001363614
	mdo|100016676	XP_001365668
Bos Taurus	bta|507447	NP_001068782
	bta|614061	NP_001069776
Sus scrofa	ssc|100156608	XP_001925867
	ssc|100739432	XP_003481520
Ailuropoda melanoleuca	aml|100465522	XP_002924616
	aml|100476235	XP_002923552
Canis familiaris	cfa|612818	XP_850302
Rattus norvegicus	rno|314613	NP_001028874
	rno|362002	NP_001102030
Mus musculus	mmu|67845	NP_080682
	mmu|70294	NP_653111
Macaca mulatta	mcc|697067	NP_001253713
	mcc|702842	NP_001248194
Pongo abelii	pon|100431951	XP_002810371
Homo sapiens	hsa|Rabring7|BCA2	AAH64903
	hsa|RNF126	NP_919442
Pan troglodytes	ptr|457980	XP_514416
Brugia malayi	bmy|Bm1_41885	XP_001899837
Trichinella spiralis	tsp|Tsp_02330	EFV56621
Caenorhabditis elegans	cel|Y54E10BR.3	CCD72989
Caenorhabditis briggsae	cbr|CBG04193	CBG04193
Pediculus humanus	phu|Phum_PHUM114800	EEB11414
Acyrthosiphon pisum	api|100163342	XP_001949741

Table S2. (continued).

Apis mellifera	ame|550689	XP_623158
Tribolium castaneum	tca|656150	XP_975905
Anopheles gambiae	aga|AgaP_AGAP002758	EAA07852
Culex quinquefasciatus	cqu|CpipJ_CPIJ010005	EDS34037
Aedes aegypti	aag|AaeL_AAEL013965	EAT33767
Drosophila grimshawi	dgr|Dgri_GH19379	EDV93545
Drosophila virilis	dvi|Dvir_GJ10303	EDW59420
Drosophila mojavensis	dmo|Dmoj_GI22582	EDW15800
Drosophila willistoni	dwi|Dwil_GK11676	EDW80709
Drosophila pseudoobscura	dpo|Dpse_GA11309	EAL28989
Drosophila persimilis	dpe|Dper_GL12113	EDW38259
Drosophila ananassae	dan|Dana_GF16575	EDV43330
Drosophila yakuba	dya|Dyak_GE25922	EDW96600
Drosophila simulans	dsi|Dsim_GD18588	EDX13686
Drosophila sechellia	dse|Dsec_GM23778	EDW42942
Drosophila melanogaster	dme|Dmel_CG11982	AAF54321
Drosophila erecta	der|Dere_GG14428	EDV49837
