# Supplementary material for: Expansion and Diversification of BTL Ring-H2 Ubiquitin Ligases in Angiosperms: Putative Rabring7/BCA2 Orthologs
Source: PLoS One. 2013 Aug 8;8(8):e72729. doi: 10.1371/journal.pone.0072729 (PMC3738576; doi:10.1371/journal.pone.0072729)
Supplement: Table S3 — Catalog of 73 sequence LOGOs generated from 502 Rabring7/BCA2/BTLs. (PDF) [file pone.0072729.s008.pdf]

Table S3. Catalog of 73 sequence LOGOs generated from 502 Rabring7/BCA2/BTLs.

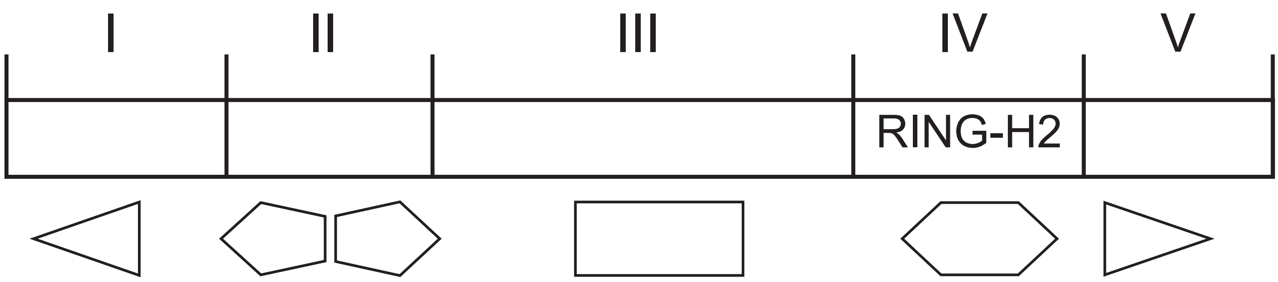

LOGO number	Symbol	Sequence
[41]	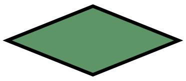	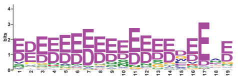
[68]	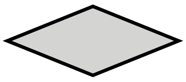	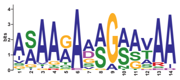
[46]	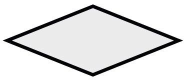	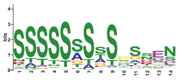
[RING-H2]	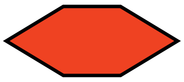	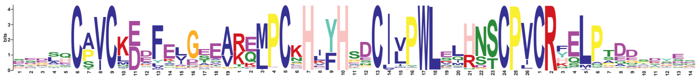
[65]	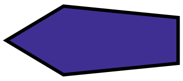	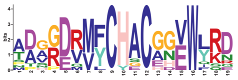
[47]	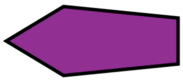	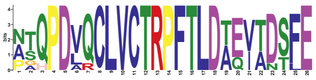
[5]	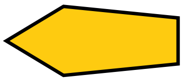	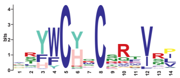
[38]	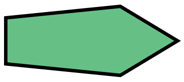	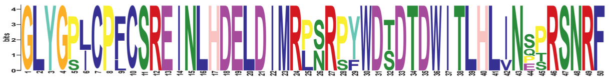
[4]	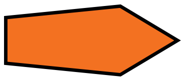	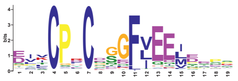
[54]	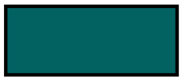	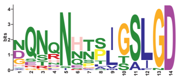
[53]	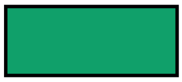	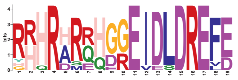
[27]	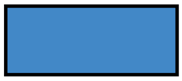	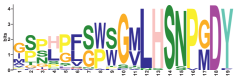
[36]	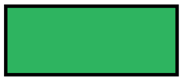	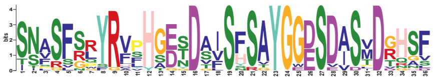
[16]	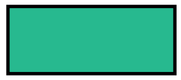	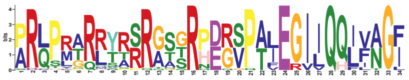

Table S3. (continued).

[70]		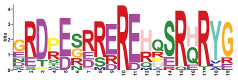
[18]		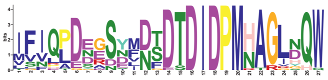
[28]		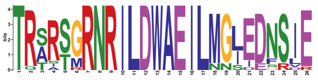
[37]		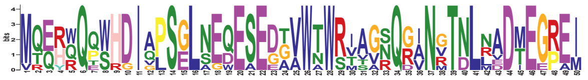
[11]		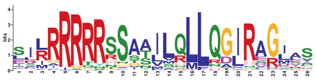
[59]		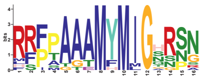
[22]		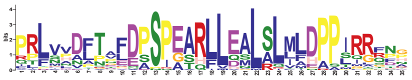
[19]		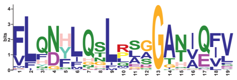
[6]		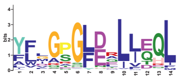
[60]		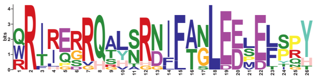
[26]		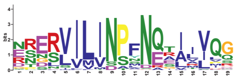
[71]		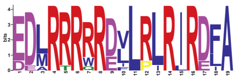
[32]		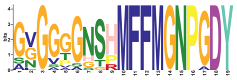
[50]		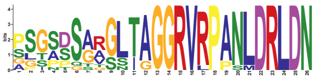
[17]		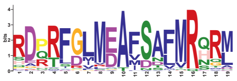
[31]		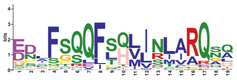
[56]		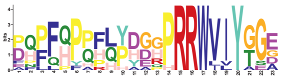
[67]		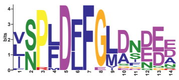
[48]		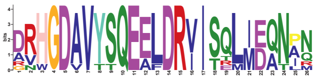

Table S3. (continued).

[2]		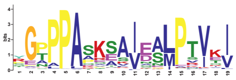
[42]		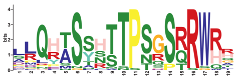
[69]		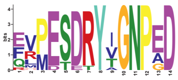
[15]		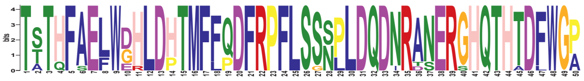
[14]		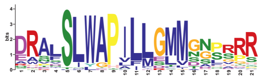
[23]		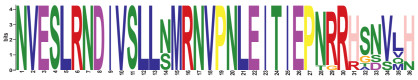
[9]		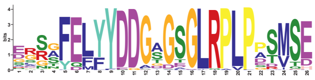
[63]		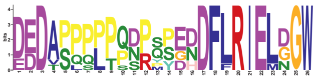
[21]		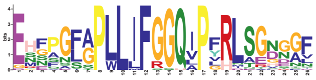
[34]		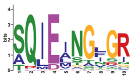
[55]		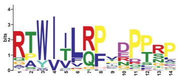
[57]		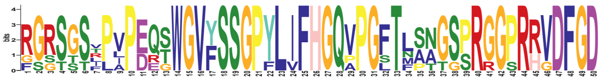
[13]		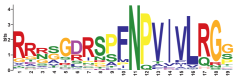
[39]		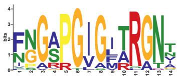
[35]		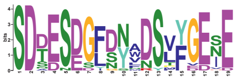
[29]		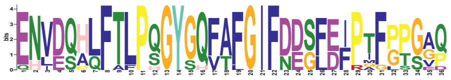
[40]		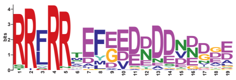
[74]		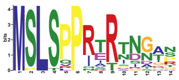
[33]		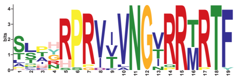

Table S3. (continued).

[30]	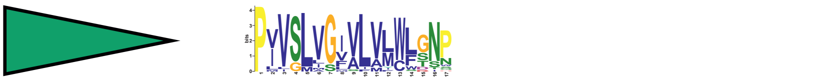
[62]	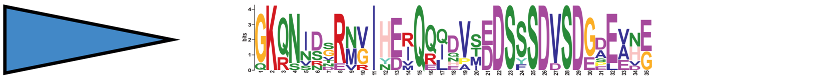
[12]	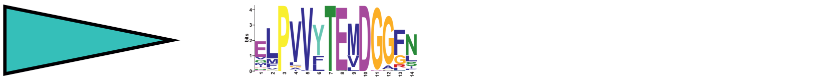
[61]	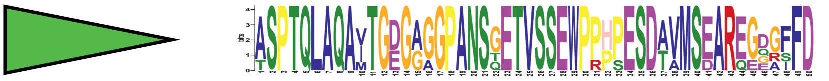
[8]	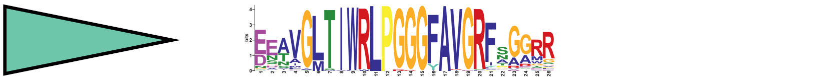
[64]	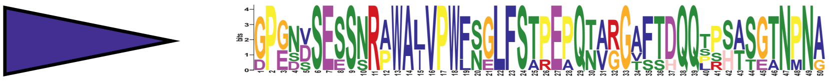
[45]	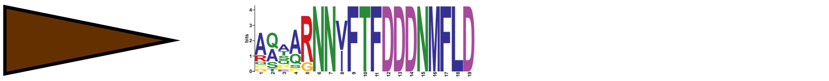
[20]	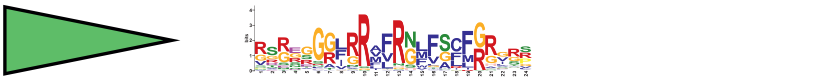
[44]	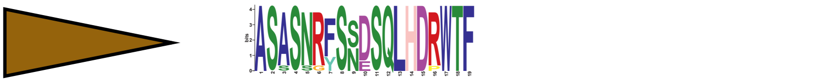
[51]	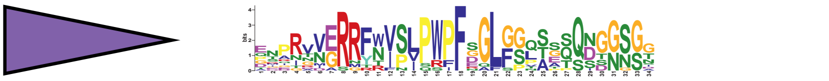
[24]	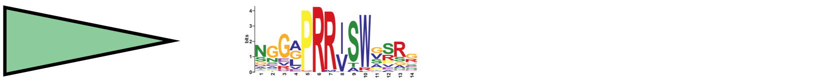
[72]	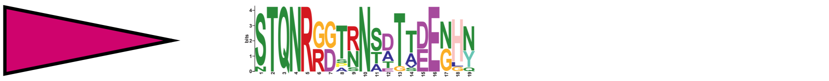
[58]	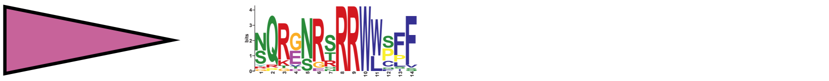
[49]	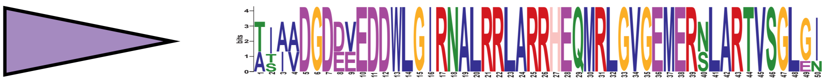
[10]	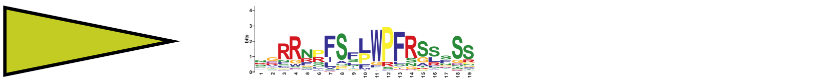
[73]	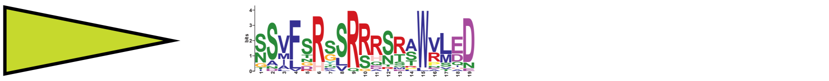
[25]	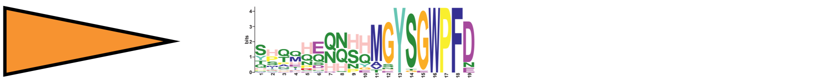
[43]	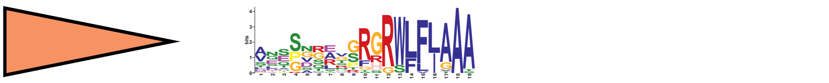
[66]	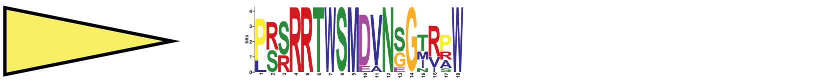
