# Supplementary material for: Expansion and Diversification of BTL Ring-H2 Ubiquitin Ligases in Angiosperms: Putative Rabring7/BCA2 Orthologs
Source: PLoS One. 2013 Aug 8;8(8):e72729. doi: 10.1371/journal.pone.0072729 (PMC3738576; doi:10.1371/journal.pone.0072729)
Supplement: Table S4 — Pairs of tandemly arrayed BTLs are shadowed in gray. A. thaliana BTLs are highlighted in yellow. (PDF) [file pone.0072729.s009.pdf]

# Table S4. Distribution of BTLs retrieved from 27 angiosperms species in 6 groups.

| | A | B | C | D | E | F |
|---|---|---|---|---|---|---|
| bdi | bdi\|Bradi1g62350<br>bdi\|Bradi2g52600 | bdi\|Bradi1g04180<br>bdi\|Bradi1g63720<br>bdi\|Bradi2g10520<br>bdi\|Bradi2g21870 | bdi\|Bradi1g66780<br>bdi\|Bradi2g04010<br>bdi\|Bradi2g39220 | bdi\|Bradi3g37800<br>bdi\|Bradi4g44277 | bdi\|Bradi1g46110<br>bdi\|Bradi3g57880 | bdi\|Bradi1g52440<br>bdi\|Bradi3g29400 |
| osa | osa\|LOC Os01g58780<br>osa\|LOC Os03g22830<br>osa\|LOC Os05g41520 | osa\|LOC Os01g16950<br>osa\|LOC Os03g20870<br>osa\|LOC Os03g59760<br>osa\|LOC Os05g40980 | osa\|LOC Os01g74040<br>osa\|LOC Os03g16480<br>osa\|LOC Os05g01940 | osa\|LOC Os08g36170<br>osa\|LOC Os11g02670<br>osa\|LOC Os12g02620 | osa\|LOC Os02g52870<br>osa\|LOC Os06g10800 | osa\|LOC Os06g01200<br>osa\|LOC Os10g34590 |
| set | set\|Si002234m.g<br>set\|Si036914m.g | set\|Si001919m.g<br>set\|Si022618m.g<br>set\|Si036169m.g<br>set\|Si036686m.g | set\|Si036971m.g | set\|Si009875m.g<br>set\|Si010014m.g<br>set\|Si013915m.g<br>set\|Si025925m.g | set\|Si008630m.g<br>set\|Si017866m.g | set\|Si036044m.g<br>set\|Si006904m.g |
| zma | zma\|AC213654.3 FG001<br>zma\|GRMZM2G164358<br>zma\|GRMZM2G176028 | zma\|GRMZM2G027120<br>zma\|GRMZM2G124441<br>zma\|GRMZM2G157246<br>zma\|GRMZM2G300589<br>zma\|GRMZM2G305264<br>zma\|GRMZM2G417125 | zma\|GRMZM2G045084<br>zma\|GRMZM2G567897<br>zma\|GRMZM5G828820 | zma\|GRMZM2G049346<br>zma\|GRMZM5G886096 | zma\|GRMZM2G021480<br>zma\|GRMZM2G142816<br>zma\|GRMZM2G473016 | zma\|GRMZM2G022175<br>zma\|GRMZM2G053210<br>zma\|GRMZM2G157855 |
| sbi | sbi\|Sb01g035310 | sbi\|Sb01g003740<br>sbi\|Sb01g036700<br>sbi\|Sb03g011120<br>sbi\|Sb09g023840 | sbi\|Sb01g039760<br>sbi\|Sb09g001100 | sbi\|Sb05g001320<br>sbi\|Sb07g022600 | sbi\|Sb04g034270<br>sbi\|Sb10g007000 | sbi\|Sb01g018800<br>sbi\|Sb10g000250 |
| aco | aco\|Aquca 014 00932 | | aco\|Aquca 009 00930<br>aco\|Aquca 009 00931 | aco\|Aquca 004 00514<br>aco\|Aquca 009 00137 | aco\|Aquca 069 00049 | aco\|Aquca 035 00058 |
| mgu | mgu\|mgv1a010352m.g | mgu\|mgv1a008674m.g | mgu\|mgv1a007795m.g | mgu\|mgv1a006570m.g<br>mgu\|mgv1a022217m.g<br>mgu\|mgv1a024323m.g | mgu\|mgv1a022478m.g<br>mgu\|mgv1a025958m.g | mgu\|mgv1a006235m.g<br>mgu\|mgv1a008418m.g |
| vvi | vvi\|GSVIVG01037379001 | vvi\|GSVIVG01025505001 | vvi\|GSVIVG01024920001<br>vvi\|GSVIVG01034134001 | | vvi\|GSVIVG01008897001<br>vvi\|GSVIVG01022971001<br>vvi\|GSVIVG01022973001 | |
| egr | egr\|Eucgr.B03765<br>egr\|Eucgr.J00661<br>egr\|Eucgr.K01683 | egr\|Eucgr.B03585<br>egr\|Eucgr.J01748 | egr\|Eucgr.A01286<br>egr\|Eucgr.G02948<br>egr\|Eucgr.J02036 | egr\|Eucgr.H00970<br>egr\|Eucgr.K01302 | egr\|Eucgr.F04366<br>egr\|Eucgr.I02719 | egr\|Eucgr.H00641<br>egr\|Eucgr.J00881 |
| ccl | ccl\|clementine0.9 016541m.g | ccl\|clementine0.9 013216m.g<br>ccl\|clementine0.9 030301m.g | ccl\|clementine0.9 012040m.g<br>ccl\|clementine0.9 013454m.g<br>ccl\|clementine0.9 014425m.g | ccl\|clementine0.9 013794m.g<br>ccl\|clementine0.9 023238m.g | ccl\|clementine0.9 031941m.g | ccl\|clementine0.9 014501m.g |
| csi | csi\|orange1.1g021656m.g | csi\|orange1.1g017208m.g | csi\|orange1.1g017269m.g | csi\|orange1.1g009095m.g | csi\|orange1.1g018299m.g | csi\|orange1.1g018689m.g |

Table S4. (continued).

| | | | | | | |
|---|---|---|---|---|---|---|
| | | csi\|orange1.1g036250m.g | csi\|orange1.1g045745m.g | csi\|orange1.1g017955m.g | | |
| | | csi\|orange1.1g043965m.g | | | | |
| cpp | cpp\|evm.TU.supercontig 39.6 | cpp\|evm.TU.supercontig 119.24 | cpp\|evm.TU.supercontig 169.13 | cpp\|evm.TU.supercontig 1460.1 | cpp\|evm.TU.supercontig 113.66 | |
| | | cpp\|evm.TU.supercontig 51.88 | cpp\|evm.TU.supercontig 19.287 | cpp\|evm.TU.supercontig 26.313 | cpp\|evm.TU.supercontig 36.37 | |
| | | | cpp\|evm.TU.supercontig 81.109 | | | |
| tha | tha\|Thhalv10006102m.g | tha\|Thhalv10012351m.g | tha\|Thhalv10002566m.g | tha\|Thhalv10005668m.g | tha\|Thhalv10025714m.g | tha\|Thhalv10011690m.g |
| | tha\|Thhalv10016932m.g | tha\|Thhalv10013831m.g | tha\|Thhalv10013762m.g | tha\|Thhalv10006114m.g | | tha\|Thhalv10021059m.g |
| | tha\|Thhalv10021967m.g | tha\|Thhalv10021230m.g | tha\|Thhalv10016765m.g | tha\|Thhalv10015834m.g | | |
| | | tha\|Thhalv10025560m.g | | | | |
| bsr | bsr\|Bra00020 | bsr\|Bra00154 | bsr\|Bra00014 | bsr\|Bra00035 | bsr\|Bra00428 | bsr\|Bra00176 |
| | bsr\|Bra00138 | bsr\|Bra00282 | bsr\|Bra00255 | bsr\|Bra00750 | bsr\|Bra02817 | bsr\|Bra03583 |
| | bsr\|Bra00458 | bsr\|Bra01910 | bsr\|Bra00502 | bsr\|Bra01426 | bsr\|Bra03395 | bsr\|Bra03824 |
| | bsr\|Bra00725 | bsr\|Bra02644 | bsr\|Bra00670 | bsr\|Bra03013 | | |
| | bsr\|Bra02986 | bsr\|Bra02743 | bsr\|Bra01938 | bsr\|Bra03780 | | |
| | | bsr\|Bra02893 | bsr\|Bra02031 | | | |
| | | bsr\|Bra03085 | | | | |
| | | bsr\|Bra03710 | | | | |
| | | bsr\|Bra03799 | | | | |
| cru | cru\|Carubv10014632m.g | cru\|Carubv10005120m.g | cru\|Carubv10016862m.g | cru\|Carubv10012728m.g | cru\|Carubv10012340m.g | cru\|Carubv10012493m.g |
| | cru\|Carubv10017636m.g | cru\|Carubv10009589m.g | cru\|Carubv10023378m.g | cru\|Carubv10017698m.g | cru\|Carubv10020847m.g | cru\|Carubv10014036m.g |
| | cru\|Carubv10023620m.g | cru\|Carubv10014141m.g | | cru\|Carubv10024842m.g | | |
| | | cru\|Carubv10026533m.g | | cru\|Carubv10028114m.g | | |
| aly | aly\|317879 | aly\|337633 | aly\|485035 | aly\|483556 | aly\|475935 | aly\|479513 |
| | aly\|324170 | aly\|478724 | aly\|496089 | aly\|486871 | aly\|922802 | |
| | aly\|483145 | aly\|492175 | aly\|934736 | aly\|496711 | | |
| | | aly\|495725 | | aly\|939042 | | |
| ath | ath\|BTL1\|AT2G40830 | ath\|BTL4\|AT5G56340 | ath\|BTL10\|AT3G46620 | ath\|BTL12\|AT5G64920 | ath\|BTL15\|AT1G68180 | ath\|BTL8\|AT3G19950 |
| | ath\|BTL2\|AT3G56580 | ath\|BTL5\|AT4G26400 | ath\|BTL11\|AT2G39720 | ath\|BTL13\|AT3G60080 | ath\|BTL17\|AT1G60360 | |
| | ath\|BTL3\|AT3G10815 | ath\|BTL6\|AT1G55530 | ath\|BTL9\|AT5G59550 | ath\|BTL14\|AT2G44330 | | |
| | | ath\|BTL7\|AT3G13430 | | ath\|BTL16\|AT5G01980 | | |
| cat | cat\|Cucsa.142510 | cat\|Cucsa.152640 | cat\|Cucsa.138820 | cat\|Cucsa.049930 | cat\|Cucsa.372780 | cat\|Cucsa.014020 |
| | cat\|Cucsa.358650 | | cat\|Cucsa.165190 | cat\|Cucsa.130800 | | cat\|Cucsa.122230 |
| pru | pru\|ppa008742m.g | pru\|ppa007335m.g | pru\|ppa006881m.g | | pru\|ppa020945m.g | |
| | | | pru\|ppa007829m.g | | pru\|ppa024978m.g | |
| | | | pru\|ppa008198m.g | | | |
| mdm | mdm\|MDP0000919900 | | mdm\|MDP0000368098 | mdm\|MDP0000675059 | | mdm\|MDP0000226252 |
| | | | mdm\|MDP0000770377 | | | mdm\|MDP0000782661 |
| mtr | mtr\|Medtr5g095810 | mtr\|Medtr3g105730 | mtr\|Medtr2g117130 | mtr\|Medtr7g080710 | mtr\|Medtr2g007460 | mtr\|Medtr1g143590 |

## Table S4. (continued).

| | | | | | | |
|---|---|---|---|---|---|---|
| | | mtr\|Medtr6g030770 | mtr\|Medtr4g084220 | | mtr\|Medtr3g097710 | |
| | | | mtr\|Medtr5g087700 | | | |
| pvu | pvu\|Phvulv091006877m.g | pvu\|Phvulv091001306m.g | pvu\|Phvulv091002571m.g | pvu\|Phvulv091000738m.g | pvu\|Phvulv091012671m.g | pvu\|Phvulv091011133m.g |
| | pvu\|Phvulv091012838m.g | | pvu\|Phvulv091005924m.g | pvu\|Phvulv091005741m.g | pvu\|Phvulv091019021m.g | pvu\|Phvulv091005768m.g |
| | pvu\|Phvulv091012846m.g | | pvu\|Phvulv091014314m.g | pvu\|Phvulv091007434m.g | | |
| | pvu\|Phvulv091021650m.g | | pvu\|Phvulv091015600m.g | | | |
| | | | pvu\|Phvulv091029702m.g | | | |
| gmx | gmx\|Glyma0024s00230 | gmx\|Glyma02g07820 | gmx\|Glyma02g41650 | gmx\|Glyma08g16830 | gmx\|Glyma04g43060 | gmx\|Glyma09g29490 |
| | gmx\|Glyma02g22760 | gmx\|Glyma16g26840 | gmx\|Glyma11g14580 | gmx\|Glyma09g40770 | gmx\|Glyma08g19770 | gmx\|Glyma10g43160 |
| | gmx\|Glyma02g44470 | gmx\|Glyma18g00300 | gmx\|Glyma11g34160 | gmx\|Glyma10g43280 | gmx\|Glyma15g05250 | gmx\|Glyma16g33900 |
| | gmx\|Glyma13g04080 | | gmx\|Glyma12g06460 | gmx\|Glyma15g42250 | | gmx\|Glyma20g23730 |
| | gmx\|Glyma13g04100 | | gmx\|Glyma13g41340 | gmx\|Glyma18g45040 | | |
| | gmx\|Glyma14g04340 | | gmx\|Glyma14g07300 | gmx\|Glyma20g23550 | | |
| | | | gmx\|Glyma15g04080 | | | |
| | | | gmx\|Glyma18g40130 | | | |
| lus | lus\|Lus10003698.g | lus\|Lus10002637.g | lus\|Lus10004712.g | | lus\|Lus10001611.g | |
| | lus\|Lus10013235.g | lus\|Lus10013397.g | lus\|Lus10024372.g | | lus\|Lus10022967.g | |
| | lus\|Lus10030755.g | lus\|Lus10020258.g | lus\|Lus10040278.g | | lus\|Lus10025612.g | |
| | | | | | lus\|Lus10028063.g | |
| pop | pop\|POPTR 0006s03070 | pop\|POPTR 0001s03880 | pop\|POPTR 0001s25010 | pop\|POPTR 0001s23810 | pop\|POPTR 0002s08630 | pop\|POPTR 0005s09270 |
| | pop\|POPTR 0016s02910 | pop\|POPTR 0003s20780 | pop\|POPTR 0006s08810 | pop\|POPTR 0006s11280 | pop\|POPTR 0012s14640 | pop\|POPTR 0012s03370 |
| | | pop\|POPTR 0013s05700 | pop\|POPTR 0008s05830 | pop\|POPTR 0016s14830 | pop\|POPTR 0015s14750 | pop\|POPTR 0007s07510 |
| | | pop\|POPTR 0019s04790 | pop\|POPTR 0009s03970 | | | pop\|POPTR 0015s05600 |
| | | | pop\|POPTR 0010s20890 | | | |
| rcu | | rcu\|28842.t000021 | rcu\|27568.t000014 | rcu\|27955.t000012 | rcu\|27732.t000004 | |
| | | rcu\|29950.t000046 | rcu\|29568.t000007 | rcu\|30170.t000615 | rcu\|29908.t000214 | |
| | | | rcu\|29751.t000117 | rcu\|30226.t000059 | | |
| msc | msc\|cassava4.1 012645m.g | msc\|cassava4.1 009285m.g | msc\|cassava4.1 009150m.g | msc\|cassava4.1 004531m.g | msc\|cassava4.1 027441m.g | msc\|cassava4.1 034211m.g |
| | | msc\|cassava4.1 009387m.g | msc\|cassava4.1 009645m.g | msc\|cassava4.1 010647m.g | msc\|cassava4.1 027470m.g | |
| | | msc\|cassava4.1 013264m.g | msc\|cassava4.1 010411m.g | msc\|cassava4.1 015095m.g | msc\|cassava4.1 033083m.g | |
| | | msc\|cassava4.1 022114m.g | msc\|cassava4.1 023055m.g | | | |

Not included: cre|Cre10.g422050 , olu|OSTLU_31341, ota|Ot04g04330, vcn|VOLCADRAFT_100265, smo|8109, smo|18753, smo|18755, smo|18758, ppp|Pp1s37_80V6, ppp|Pp1s161_109V6, ppp|Pp1s223_128V6, set|Si004415m.g, mgu|mgv1a010758m.g., gr|Eucgr. A02079, cpp|evm.TU.supercontig_1036.1, cpp|evm.TU.supercontig_34.207, and aly|936073.
