# Supplementary material for: Expansion and Diversification of BTL Ring-H2 Ubiquitin Ligases in Angiosperms: Putative Rabring7/BCA2 Orthologs
Source: PLoS One. 2013 Aug 8;8(8):e72729. doi: 10.1371/journal.pone.0072729 (PMC3738576; doi:10.1371/journal.pone.0072729)
Supplement: Table S5 — The ath|BTL4 interactors mapped to region III. (PDF) [file pone.0072729.s010.pdf]

Table S5. The ath|BTL4 interactors mapped to region III.

Arabidopsis Gene Index	Description
At1g67750	Pectate lyase family protein
At1g01800	NAD(P)-binding Rossmann-fold superfamily protein
At1g05600	Tetratricopeptide repeat (TPR)-like superfamily protein
At1g67750	Pectate lyase family protein
At1g76060	EMB1793, LYR family of Fe/S cluster biogenesis protein
At3g50500	SNF1-RELATED PROTEIN KINASE 2-2
At4g30620	Uncharacterised BCR, YbaB
At5g25757	RNA polymerase I-associated factor PAF67
At5g35630	GS2, GLN2, ATGSL1, glutamine synthetase 2
At5g43880	Protein of unknown function (DUF3741)
At5g54380	THESEUS1 THE1, protein kinase family protein
At5g63190	MA3 domain-containing protein
At5g63940	Protein kinase protein, adenine nucleotide alpha hydrolases-like domain
